# Confronting pastoralists’ knowledge of cattle breeds raised in the extensive production systems of Benin with multivariate analyses of morphological traits

**DOI:** 10.1371/journal.pone.0222756

**Published:** 2019-09-26

**Authors:** Sandrine O. Houessou, Luc Hippolyte Dossa, Rodrigue Vivien Cao Diogo, Maurice Cossi Ahozonlin, Mahamadou Dahouda, Eva Schlecht

**Affiliations:** 1 Ecole des Sciences et Techniques de Production Animale, Faculté des Sciences Agronomiques, Université d’Abomey- Calavi, Bénin; 2 Département des Sciences et Techniques de Productions Animale et Halieutique, Université de Parakou, Faculté d’Agronomie, Bénin; 3 Animal Husbandry in the Tropics and Subtropics, University of Kassel and University of Göttingen, Germany; University of Illinois, UNITED STATES

## Abstract

Cross-border seasonal livestock movements in West Africa bring into close contact several cattle breeds. In the coastal countries hosting migrating herders from the Sahel, it often affects the genetic variability and geographical distribution of traditional cattle breeds, through their indiscriminate but also intended crossbreeding with larger-framed Sahelian cattle breeds. The need to secure and effectively manage this genetic variability, in order to respond to changing production and market conditions, is widely recognized by the scientific community, livestock herders and policy-makers. This however requires a comprehensive knowledge of the breeds’ characteristics. The indigenous criteria used by pastoralists to characterize and distinguish cattle breeds remain unclear and further validation is required. This study was therefore designed to document and validate herders' knowledge on cattle breeds. From June 2015 to June 2016, 803 cattle herders participated in a phenotypic breed description in seven pastoral communities across the country. Each cattle herder was asked to name and describe morphologically the different cattle breeds in his herd. Subsequently, fifteen body measurements taken on a total of 1401 adult cattle (964 cows and 439 bulls) were submitted to multivariate analyses. Participants distinguished ten different cattle breeds kept in traditional herds according to six primary morphological traits and clearly separated zebuine from taurine breeds. These results were consistent with those of the multivariate analyses of the measured traits. However, herders’ classification approach proved to be more accurate in distinguishing breeds within the zebuine subspecies. Hence, while metric measurements and molecular genetic analyses are promising approaches to fill the knowledge gap on the diversity of local farm animal genetic resources, they should integrate livestock herders’ traditional knowledge for more precision.

## Introduction

West Africa is rich in a wide variety of cattle breeds represented by the taurine (*Bos taurus)* and Zebu subspecies [1,2]. Exposed to complex social, political and environmental processes over centuries, these genetic resources have developed under harsh conditions to which they have adapted [3]. They are appreciated for their adaptive traits which include the resistance to diseases and drought, ability to walk long distances, and capacity to survive on poor pastures [2]. Yet, this valuable diversity is increasingly threatened by genetic dilution due to changes in production systems, livestock herders' preferences for specific breeds and/or traits, market conditions and opportunities [4]. Several studies revealed admixtures among the taurine and zebu subspecies [5,6,7] as the result of the continuous genetic flow that occurs every year during seasonal cross-border livestock movements from the drier Sahelian zones in the north to the more humid zones in the south of West and East Africa. The importance of these local genetic resources for the livelihoods of livestock herders and the sustainability of the production systems in which they are raised calls for the urgent need to promote their sustainable use [2,8,9,10] and conservation [11]. The latter author has argued that biodiversity conservation and food security are two sides of the same coin.

Yet, as revealed by the review of existing literature, the first obstacle to sustainable management of local farm animal genetic resource in African livestock production systems is the insufficient knowledge on their specific features and genetic diversity [2,8]. In Benin, like in several African countries, there are virtually no inventories of these resources and thus no reliable data available. The precise identification of animal types and breeds, and an improved understanding of their values or adaptive traits are thus necessary but depend on the availability of accurate and comprehensive information on their characteristics as well as their production and marketing environments.

The Second Report on the State of the World’s Animal Genetic Resources for Food and Agriculture [12] acknowledged recent and ongoing efforts of description and characterization of livestock breeds in several West African countries. But most of these efforts tend to be fragmentary and limited either to their phenotypic [13,14,15,16], or genotypic and molecular characterization [7,17,18,19,20,21] out of the production system context, paying little attention to the local knowledge of the communities who keep them. Given its importance, livestock herders’ indigenous knowledge has been recommended to be an integral part of breed characterization [22]. This knowledge is mainly useful in quantitative morphological characterization that represents the first step in the characterization process [23,24,25] and can provide, to some extent, a reasonable representation of genetic difference among populations [26]. This is more evident in Sub-Saharan Africa, where, cattle are basic assets of cattle herders who mostly are still involved in pastoralism, which is still the dominant ruminant livestock system [27]. Hence, cattle breeds are subject of much discussion among these herders [28] who have accumulated a wealth of untapped knowledge of these farm animal genetic resources and of their production environments [29,30,31,32].

Multivariate discriminant analyses of morphological traits have been reported, in several previous studies, to be effective for a precise and objective discrimination of different populations of cattle [13,14,15,33]; goats [26,34], sheep [35,36,37], and horses [38]. Therefore, results of such analyses may represent an objective basis for comparison with herders’ indigenous knowledge. So far, however, no studies of which we are aware combine the two approaches in order to test their complementarity and validate pastoralists’ classification of cattle breeds. This study aimed to document and validate herders' knowledge of differences among cattle breeds raised in Benin with quantitative data.

## Material and methods

### Study sites

**Fig 1:**
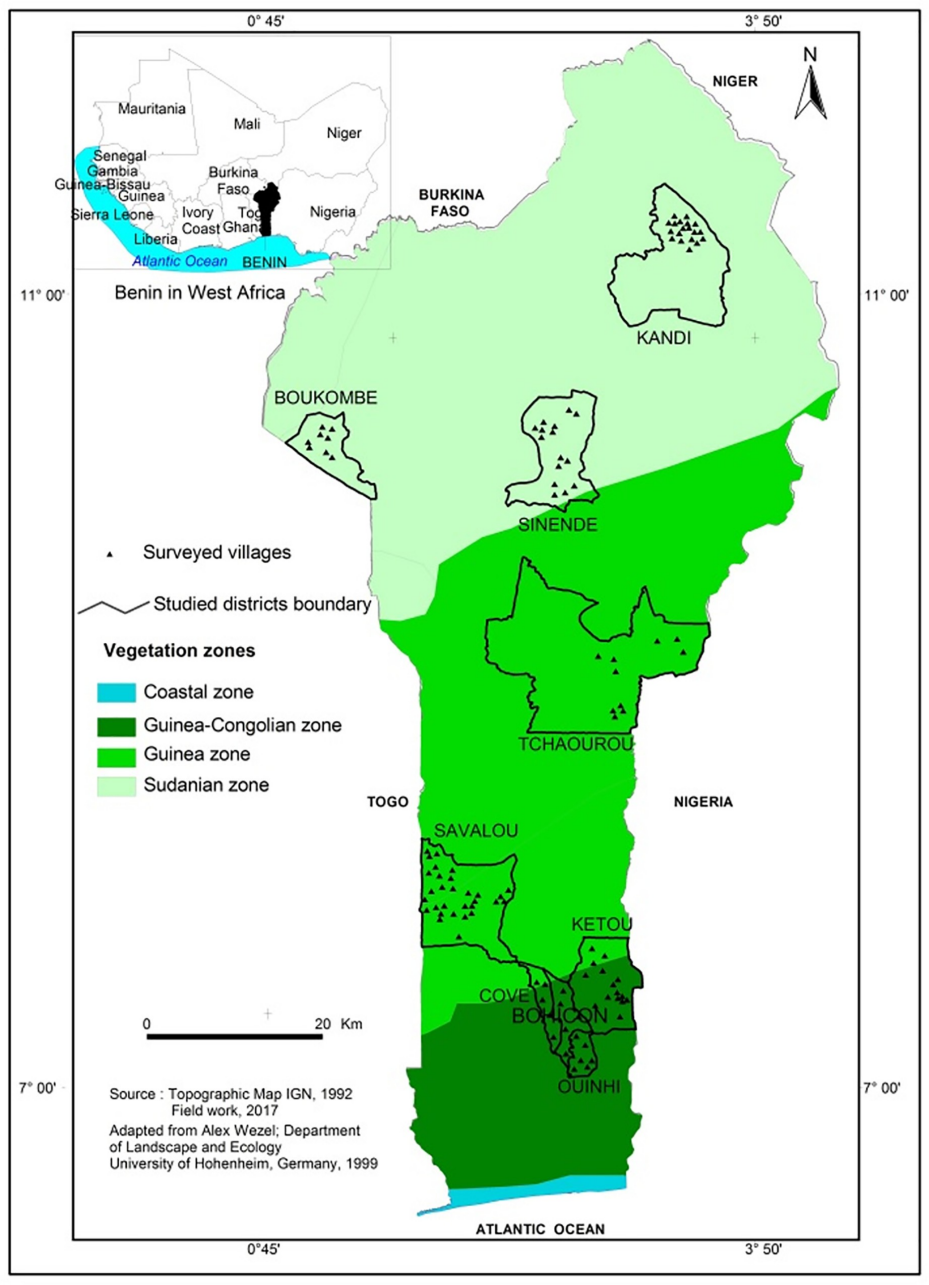
Map of Benin showing the locations of the municipalities investigated.

The study was conducted in seven (07) localities which are representative of three vegetation zones along the geographical north-south gradient in Benin (Fig 1). Ferralitic soils and a bimodal rainfall with an average annual precipitation of 1250 mm characterize the regions of Kétou and Agonli in the Guinea-Congolian zone (GCZ). In the Guinean zone (GSZ), which includes Savalou and Tchaourou, the soils are of ferruginous type and the annual precipitation averages 1150 mm with a bimodal and unimodal rainfall pattern in Savalou and Tchaourou respectively. The Sudanian zone (SZ) included Kandi, Sinendé and Boukombé. It is characterized by ferruginous soils and a bimodal rainfall pattern with an average annual precipitation of 1000 mm. These locations, whose some demographic characteristics are presented in Table 1, were selected because of the presence of relatively high numbers of resident cattle herds but also because of the important influx of seasonal and cyclical migrating herds in search of pasture and water. These migrating herds are further designed as transhumant herds.

**Table 1:** General characteristics of the study locations and number of cattle herds surveyed in Benin.

Location	Geographic coordinates	Climate ^[^^39^^]^	Area (km^2^)	Population density (people/km^2^)^[^^40^^]^	Estimates of the total cattle population (heads) ^[^^41^^]^	Cattle herds sampled (n)
**Kétou**	7° 21′ N 2° 36′ E	Sub-equatorial	1775	88	16000	120
**Agonli**	7° 13′ N 2° 20′ E	Sub-equatorial	1758	132	6170	50
**Tchaourou**	8° 53′ N 2° 36′ E	Tropical subhumid	7256	30	47000	110
**Savalou**	7° 55′ N 1° 58′ E	Tropical subhumid	2674	54	32000	120
**Kandi**	‎11° 07’N 2° 56’ E	Dry tropical	3421	52	159000	148
**Sinendé**	10° 20′ N 2° 22′ E	Dry tropical	2289	39	80000	135
**Boukombé**	10° 11′ N 1° 06′ E	Dry tropical	1 036	80	30100	120

## Data collection

### Ethical statement

The study involved taking body measurements from cattle with the consent and in the presence of the cattle herder. There is no specific legislation for body measurements and hence no approval was necessary. All the data was collected in traditional farms and the animal owners agreed to be involved in the project through the Communal Sector for Agricultural Development (SCDA) which is the decentralized institution for the management of the agricultural sector in the surveyed municipalities. All the animals included in this study were managed in accordance with the criteria for the assessment of animal welfare identified by the Welfare Quality Project (WQP) [42].

### Assessment of pastoralists’ knowledge about cattle breeds’ characteristics

First, informal interviews were organized with all actors involved in livestock production, including officers of local extension services in each of the research locations, to obtain some preliminary information about cattle production, lists of cattle camps and of the places where cattle herds and herders gather.

Individual interviews were then conducted from June 2015 to June 2016 with 803 resident cattle herders randomly selected from those who had at least 10 years of experience in cattle herding and had spent at least five (05) years in the locality. Unequal distribution of the livestock camps resulted in unbalanced samples across the studied villages (Table 1). The herders were asked to name in their local language the breeds of cattle kept in their area and to indicate the most relevant traits they used to distinguish between them. Breed was defined according to [43] as: “either a sub-specific group of domestic livestock with definable and identifiable external characteristics that enable it to be separated by visual appraisal from other similarly defined groups within the same species, or a group for which geographical and/or cultural separation from phenotypically similar groups has led to acceptance of its separate identity”. Thus, cattle breed in this study is referred to cattle of similar physical features perceived by herders as being of the same genetic origin.

### Morphological characterization

In each surveyed herd, one mature cow and, where available, one breeding bull representative of each cattle breed present and named by the herder were then randomly selected for morphometric measurements. They maturity was ascertained by the visual examination of their dentition (possession of either three or four pairs of permanent teeth). A total of 1401 adult cattle of both sexes (943 cows and 458 bulls) was measured and included in this study.

Fifteen body measurements and ten qualitative traits (Table 2) were assessed following the FAO guidelines for phenotypic characterization of animal genetic resources [25]. The qualitative traits described were sex, presence of horn, horn color, horn shape, cephalic profile, ear shape, ear orientation, body coat color and body coat color pattern, presence of hump and dewlap size. The body measurements were carried out using a measuring stick, a measuring tape or wooden caliper on animals standing on a level surface and maintained in upright posture by their respective owners.

**Table 2:** Morphological traits measured on 1401 individual cattle across seven locations in Benin.

Variable	Description
**Quantitative** (in cm)	
Height at withers (WH)	Vertical distance from the bottom of the front foot to the highest point of the shoulder between the withers
Rump height (RH)	Distance from the highest point of rump to the ground
Heart girth (HG)	Circumference of body just behind the forelegs
Body length (BL)	Distance between the horn site to tail drop
Scapula-ischial length (SIL)	Distance from tip of the shoulder to the ischial tuberosity
Face length (FAL)	Distance from between the horn site to the lower lip
Ear length (EL)	Distance from the point of attachment to the tip of the ear
Head width (HW)	Distance between the most prominent points of the zygomatic arches
Tail length (TL)	Distance from the tail drop to the tip of the tail
Hip Width (HW)	Distance between the ends of the bone of the iliac crest
Horn length (HL)	Distance from the root of the horn to its tip along the outer curvature
Hock circumference (HC)	Circumference taken just above the hock joint
Muzzle circumference (MC)	Complete distance around the outside of the mouth
Chest depth (CD)	Vertical distance from the apex of the withers to the bottom of the chest
Shoulder point width (SPW)	Distance between the right and left shoulder points
**Qualitative**	
Sex	Male, female
General aspect of the coat	Uniform, Spotted, Composed
Unique color of coat	Black, White, Dark red, Brown, Fawn
Other color of coat	White spotted black, Black spotted white, White spotted red, Red spotted white
Cephalic profile	Concave, Convex, Straight
Presence of hump	Absent, Present
Presence of horn	Absent, Present
Color of horn	Black, Brown, White, Black and Brown, Black and White, Brown and white
Horn shape	Straight, Crown, Cup, Folded back cup, Crescent, Lyre, Folded back lyre, Wheel, Spiral, Numeral three
Ear shape	Rounded, Pointed
Orientation of ear	Erected, Horizontal, Dropping

### Statistical analysis

Collected data were processed and analyzed using SAS software, version 9.2 (SAS Institute, Inc., Cary, NC, USA). Metric data were first checked for consistency. This resulted in the exclusion of the five animals named by herders as of *Djelliji* breed and one male of *Dageeji* breed, which are not included in the 1401 individual cattle.

Descriptive analyses were firstly performed to explore statistical differences among the different cattle breeds. Frequencies and chi-square (χ2) tests for independence were performed on the aforementioned qualitative traits to explore statistical differences among breeds. Similarly, the quantitative variables were subjected to analysis of variance using PROC GLM. The least square means (LSMEANS) were calculated for males and females separately, and for both together. Comparison of means between groups was performed using the Students Newman and Keuls (SNK) multiple mean comparison tests. The results were used to screen for the most useful variables for further discriminant analysis. The canonical discriminant analysis using the CANDISC procedure was performed to determine the best linear combination of the quantitative variables that would group or separate the named cattle breeds. Canonical variables that summarize between-breeds variation were generated and the pairwise squared Mahalanobis distances calculated. PROC GPLOT was used to plot the individuals onto canonical variables for visual examination of the ordering of the different breeds in the multivariate space. The ability of these canonical functions to assign each individual animal to its original group was calculated as the percentage of correct assignment to each genetic group using the DISCRIM procedure (Nearest Neighbour Discriminant Analysis). The degree of morphological similarity or divergence between the investigated cattle breeds was assessed and their classification in homogenous groups was carried out using the method of Ascending Hierarchical Clustering (AHC) according to the criterion of the jump of Ward in the PROC CLUSTER and TREE procedures. The Mahalanobis distances generated during the canonical discriminant analysis were used to construct a dendrogram using the Unweighted Pair Group Method Analysis (UPGMA). Finally, the association between the qualitative traits was investigated through a Multiple Correspondence Analysis (MCA) using PROC CORRESP.

## Results

### Herders’ perceptions of morphological characteristics of the main cattle breeds

A total of ten cattle breeds were identified from the surveyed herders’ responses and named in *Fulfulde* language, spoken by almost all of the herders, as follows: *Boboji*, Somba, *Yakanaji*, Goudali/*Bokoloji*, *Bodeeji/*Bororo, *Djelliji*, *Dageeji/Dage*, *Bargouji/Muti*, *Keteeji* and one unnamed type perceived as crossbreed.

The following morphological traits were used by herders to distinguish among different cattle breeds: body size (dwarf, small, middle, large), size of horn (absent, short, middle, long, long and white), hump size (absent, small, poorly developed, well developed), size of sheath/ umbilical fold (small, poorly developed, well developed), dewlap size (small, poorly developed, well developed), coat color (white, reddish-brown/ black, white/reddish, variable, white and black neck).

Table 3 presents the characteristics of the different cattle breeds according to the participating herders. The two taurine breeds, namely Somba and *Boboji* (S1 and S2 Figs), were described with similar characteristics (variable coat color, very small size, short horns and absence of hump).

**Table 3:** Key morphological traits used by herders (%) to classify nine cattle breeds raised in Benin.

Trait	Trait expression	Cattle breeds							
		*Yakanaji*	*Goudali*	*Bodeeji*	*Djelliji*	*Dageeji*	*Bargouji/Keteeji*	*Boboji*	Somba
**Coat color**		(n = 407)	(n = 463)	(n = 422)	(n = 84)	(n = 261)	(n = 298)	(n = 111)	(n = 100)
	White	100.0	84.9	0.0	0.0	100.0	95.6	0.0	0.0
	Reddish Brown / Black	0.0	0.0	100.0	0.0	0.0	0.0	0.0	0.0
	White/Reddish	0.0	0.0	0.0	98.8	0.0	0.0	0.0	0.0
	Variable	0.0	0.0	0.0	1.2	0.0	4.4	100.0	100.0
	White and black neck	0.0	15.1	0.0	0.0	0.0	0.0	0.0	0.0
**Body size**		(n = 477)	(n = 396)	(n = 376)	(n = 215)	(n = 217)	(n = 400)	(n = 215)	(n = 220)
	Large	95.6	100.0	99.7	96.7	0.0	0.0	0.0	0.0
	Medium	4.4	0.0	0.0	3.3	100.0	0.0	0.0	0.0
	Small	0.0	0.0	0.3	0.0	0.0	100.0	0.0	0.0
	Dwarf	0.0	0.0	0.0	0.0	0.0	0.0	100.0	100.0
**Size of horn**		(n = 527)	(n = 557)	(n = 418)	(n = 209)	(n = 251)	(n = 380)	(n = 170)	(n = 219)
	Absent	0.0	100.0	0.0	0.0	0.0	0.0	0.0	0.0
	Long	100.0	0.0	100.0	0.0	100.0	0.0	0.0	0.0
	Medium	0.0	0.0	0.0	100.0	0.0	0.0	0.0	0.0
	Short	0.0	0.0	0.0	0.0	0.0	100.0	100.0	100.0
**Size of hump**		(n = 337)	(n = 374)	(n = 242)	(n = 202)	(n = 116)	(n = 418)	(n = 138)	(n = 210)
	Absent	0.0	0.0	0.0	0.0	0.0	35.6	100.0	100.0
	Well developed	100.0	100.0	62.8	100.0	19.0	0.0	0.0	0.0
	Poorly developed	0.0	0.0	37.2	0.0	81.0	0.0	0.0	0.0
	Small	0.0	0.0	0.0	0.0	0.0	64.4	0.0	0.0
**Size of dewlap**		(n = 174)	(n = 223)	(n = 163)			(n = 120)		
	Well developed	0.0	100.0	0.0	-	-	0.0	-	-
	Poorly developed	100.0	0.0	100.0	-	-	0.0	-	-
	Small	0.0	0.0	0.0	-	-	100.0	-	-
**Size of sheath/ umbilical folds**		(n = 202)	(n = 247)	(n = 54)		(n = 44)	(n = 82)		
	Well developed	100.0	100.0	0.0	-	0.0	0.0	-	-
	Poorly developed	0.0	0.0	100.0	-	36.4	0.0	-	-
	Small	0.0	0.0	0.0	-	63.6	100.0	-	-

The five zebu breeds (*Yakanaji*, *Goudali*, *Bodeeji*, *Djelliji*, and *Dageeji)* (S3–S7 Figs) also shared many similarities. With the exception of the hornless *Goudali* and *Djelliji* whose horn was perceived by respondents as of medium size, the zebus were, in their majority, described as large-framed and long-horned animals with a well or poorly developed hump. The Bodeeji breed was further differentiated from the Yakanaji breed by its reddish brown to red dark coat color and white horns.

Moreover, the *Dageeji* breed, in contrast to others, was perceived as a medium-sized zebu with a poorly developed hump and a small sheath/ umbilical fold.

The *Bargouji* breed (S8 Fig) was described as a shorthorn taurine-like cattle, but with a larger body size than taurine, a white coat, absence of hump or presence of small hump, a small dewlap and a small sheath/ umbilical fold. The morphological characteristics of *Keteeji* cattle were not presented separately because herders perceived *Bargouji/Muti* and *Keteeji* as two morphologically close cattle breeds. They however assumed that the pure *Bargouji* is generally humpless or has only a small hump whereas *Keteeji* is always humped with a small or poorly developed hump. They also asserted that *Keteeji* is sometimes larger in size than *Bargouji*.

### Comparison of measured morphometric traits among breeds

The differences between the cattle breeds for the morphometric variables of male and female animals and their pooled data are presented in Table 4, Table 5 and S1 Table, respectively. All linear body measurements significantly varied (P<0.05) among breeds. Major morphological traits such as height at withers (WH), rump height (RH), body length (BL) and scapulo-ischial length (SIL) were significantly greater (P<0.05) for humped cattle breeds compared with humpless ones. Within breeds, they were also significantly greater (P<0.05) in bulls than in cows. The highest mean values of WH were recorded in *Bodeeji* cows followed by the *Yakanaji* cows whereas the lowest values were obtained in the *Boboji* and Somba cows. In terms of morphometric traits, these two taurine breeds presented no significant differences.

**Table 4:** Least square means (± standard error) and pairwise comparison of morphological traits measured in cows across nine cattle breeds raised in Benin.

Trait	Cattle breeds								
	*Bargouji*	*Boboji*	*Bodeeji*	*Dageeji*	*Goudali*	*Keteeji*	Crossbreed	Somba	*Yakanaji*
	(n = 206)	(n = 58)	(n = 26)	(n = 23)	(n = 22)	(n = 138)	(n = 94)	(n = 84)	(n = 312)
**MC**	40.6^c^ ± 0.37	43.1^b^ ± 0.60	46.5^a^± 0.52	44.0^b^± 0.44	40.3^c^± 1.07	46.8^a^± 0.32	43.6^b^±0.36	40.1^c^± 0.37	44.5^b^±0.21
**HW**	19.5^b^ ± 0.15	19.2^b^ ± 0.23	20.1^b^±0.18	19.2^b^± 0.28	20.4^b^± 0.57	23.5^a^±0.33	20.3^b^±0.24	17.4^c^± 0.16	20.4^b^±0.15
**FAL**	45.7^c^ ± 0.42	43.9^d^ ± 0.56	48.4^b^ ±0.21	47.6^b^^c^± 0.67	46.0^c^±0.60	46.9^a^± 0.47	46.9^b^^c^±0.36	39.9^e^± 0.41	46.8^b^^c^±0.3
**EL**	19.5^c^^d^ ± 0.22	17.4^e^ ± 0.22	20.6^c^ ± 0.26	19.0^d^± 0.23	21.9^b^± 0.63	23.4^a^ ±0.48	20.5^c^±0.27	15.4^f^± 0.13	20.7^c^±0.18
**HL**	37.0^e^ ± 0.51	28.1^f^ ± 1.46	65.8^a^ ±1.86	49.1^b^^c^± 1.74	14.1^g^± 4.10	47.7^c^ ±1.04	42.4^d^±1.51	15.8^g^± 1.46	52.8^b^±0.68
**HG**	152.8^d^ ±0.99	148.9^d^ ± 1.49	179.4^a^±1.42	169.1^b^ ±2.12	171.8^b^±2.60	159.1^c^±0.91	166.4^b^±1.15	142.5^e^± 1.42	171.4^b^±0.93
**HC**	35.6^d^ ±0.25	39.6^c^ ± 0.44	44.3^b^ ±0.60	47.4^a^ ±0.93	40.4^c^±0.86	38.5^c^±0.26	44.5^b^±0.45	35.4^d^± 0.54	43.5^b^±0.62
**TL**	99.0^a^^b^ ± 0.83	87.6^b^ ± 1.01	103.6^a^^b^± 1.06	97.8^a^^b^± 0.69	103.9^a^^b^±2.50	102.7^a^^b^±1.35	109.7^a^±9.65	91.7^a^^b^± 0.74	103.1^a^^b^±0.65
**SPW**	34.7^b^ ± 0.27	28.2^d^ ±0.44	33.3^a^± 0.76	30.4^c^± 0.42	33.9 ±1.03	36.4^a^±0.44	32.3^b^±0.42	29.0^c^^d^ ±0.43	36.6^b^±0.38
**HW**	42.7^b^ ± 0.23	41.0^c^ ±0.51	45.8^a^± 0.63	44.3^a^^b^±0.37	46.0^a^±1.01	45.1^a^±0.31	44.3^a^^b^±0.35	36.4^d^ ±0.37	46.4^a^±0.25
**CD**	63.9^a^ ±0.35	54.6^c^± 0.72	66.1^a^±0.68	64.6^a^±0.90	64.9^a^± 1.46	67.0^a^±0.51	60.8^b^±0.46	49.8^d^± 0.37	65.7^a^±0.51
**WH**	115.9^d^ ± 0.63	110.5^f^±0.83	137.8^a^± 0.85	129.1^b^± 1.32	130.3^b^ ±1.30	131.1^b^±0.53	123.4^c^±0.78	100.4^f^±0.47	131.4^b^±0.76
**RH**	117.4^d^ ±0.74	113.1^e^±0.85	136.4^a^±0.68	129.0^b^^c^±1.02	129.8^b^^c^±1.05	134.1^a^±0.61	125.4^c^±0.74	105.3^f^±0.51	132.4^b^±0.65
**BL**	115.9^d^ ± 0.63	114.6^d^±1.07	132.6^a^±0.94	124.4^b^^c^± 1.18	120.8^c^± 2.44	129.6^a^±0.85	121.7^c^±0.74	100.2^e^±0.76	128.2^b^±0.66
**SIL**	164.2^d^ ±0.75	150.4^e^±1.74	193.2^a^± 2.69	180.3^b^^c^±1.49	173.9^c^± 1.97	175.1^c^±1.25	177.0^c^± 2.22	141.1^f^±0.78	184.4^b^±1.39

^*abcdefg*^ Means with different superscript in the same row are significantly different (P ≤ 0.001), SNK’s multiple mean comparison test

MC: Muzzle circumference, HW: Head width, FAL: Face length, EL: Ear length, HL: Horn length, HG: Heart Girth, HC: Hock circumference, TL: Tail length, SPW: Shoulder point width, HW: Hip Width, CD: Chest depth, WH: Withers height, RH: Rump height, BL: Body length, SIL: Scapula-ischial length.

**Table 5:** Least square means in cm (± standard error) and pairwise comparison of morphological traits measured in bulls of nine cattle breeds raised in Benin.

Traits				Cattle breeds				
	*Bargouji*	*Boboji*	*Bodeeji*	*Goudali*	*Keteeji*	Crossbreed	Somba	*Yakanaji*
	(n = 131)	(n = 5)	(n = 10)	(n = 10)	(n = 93)	(n = 16)	(n = 109)	(n = 63)
**MC**	43,5^b^±0,31	44,8^a^^b^^c^±1,96	44,2^a^^b^^c^±0,94	45,6^a^^b^^c^±1,09	46,2^a^±0,39	47,5^a^±1,19	42,2^b^±0,34	48,0^a^±0,52
**HW**	20,1^a^^b^±0,17	19,8^a^^b^±0,20	21,6^a^^b^±0,58	22,0^a^^b^±1,01	21,8^a^^b^±0,27	23,1^a^±1,02	18,0^b^±0,17	22,5^a^±0,38
**FAL**	49,2^a^±0,28	45,8^b^±1,74	48,9^a^±0,97	48,2^a^±0,79	51,2^a^±0,30	50,0^a^±1,18	40,1^b^±0,38	50,4^a^±0,56
**EL**	19,8^a^±0,21	17,4^b^±0,60	21,0^c^±0,54	21,9^c^±0,62	21,9^c^±0,27	20,6^c^±0,52	15,9^b^±0,18	21,7^c^±0,29
**HL**	34,6^b^±0,82	28,2^b^^c^±3,77	52,8^a^±3,03	16,3^c^±5,07	42,3^a^^b^±1,06	40,8^a^^b^±4,65	16,8^c^±0,52	53,0^a^±1,79
**HG**	155,4^c^^d^±0,79	155,9^c^^d^±5,04	160,2^b^^c^±1,33	180,1^a^±3,03	162,0^b^^c^±0,97	174,9^a^^b^±3,84	144,2^d^±0,74	174,7^a^^b^±1,99
**HC**	34,6^b^±0,31	38,7^b^±0,86	46,5^a^±1,23	47,9^a^±1,20	38,2^b^±0,28	48,9^a^±0,90	37,9^b^±0,31	46,4^a^±0,70
**TL**	101,8^a^^b^±1,25	81,7^c^±2,53	106,8^a^^b^±2,14	118,4^a^±3,56	107,7^a^^b^±1,57	110,4^a^^b^±2,49	93,4^b^^c^±0,85	109,9^a^^b^±1,52
**SPW**	34,8^a^^b^±0,33	29,2^b^±0,80	33,6^a^^b^±0,88	35,4^a^^b^±1,87	36,4^a^±0,43	35,1^a^^b^±0,98	29,0^b^±0,35	36,6^a^±0,63
**HW**	42,5^a^±0,35	41,0^a^^b^±2,07	42,9^a^±1,09	44,9^a^±1,56	43,7^a^±0,41	45,5^a^±0,91	36,4^b^±0,40	47,4^a^±0,55
**CD**	62,9^b^±0,40	56,6^b^±1,40	59,0^b^±0,61	65,4^a^^b^±1,22	64,7^a^^b^±0,48	63,6^a^^b^±1,40	51,8^b^±0,48	68,5^a^±0,90
**WH**	117,0^b^±0,58	109,2^b^^c^±1,22	137,0^a^±1,41	135,1^a^±1,41	128,8^a^^b^±0,52	130,4^a^±2,09	101,8^c^±0,51	136,5^a^±0,94
**RH**	123,6^b^^c^±0,49	111,6^c^±2,87	139,7^a^±1,89	139,0^a^±1,58	133,8^a^±0,59	134,3^a^±2,00	107,0^c^±0,57	138,2^a^±0,64
**BL**	119,4^b^±0,71	115,8^b^^c^±5,26	134,1^a^±2,26	132,1^a^±4,53	123,2^b^±0,94	124,4^b^±1,92	103,2^c^±0,67	131,8^a^±1,02
**SIL**	163.8^b^±1.12	151.4^c^±1.70	198.4^a^± 0.90	182.8^b^± 1.46	173.6^a^^b^±3.15	184.8^a^± 3.10	144.7^c^±1.70	186.8^a^±1.39

^*abcd*^ Means with different letters in rows are significantly different between locations at P ≤ 0.001; SNK’s multiple mean comparison test

MC: Muzzle circumference, HW: Head width, FAL: Face length, EL: Ear length, HL: Horn length, HG: Heart Girth, HC: Hock circumference, TL: Tail length, SPW: Shoulder point width, HW: Hip Width, CD: Chest depth, WH: Withers height, RH: Rump height, BL: Body length, SIL: Scapula-ischial length

Within the recorded qualitative traits, only a few (presence of hump, shape and orientation of horns002C size of dewlap) were useful in discriminating the zebus from the taurine breeds (S2 and S3 Tables). The white color was dominant in the zebus except for the zebu *Bodeeji* which generally presented a single reddish brown coat color. Taurine and crossbreed presented variable coat colors. In addition, about half of the animals sampled as of the *Bargouji* breed had no hump, and the same observation was made for the *Keteeji* breed.

### Typology of cattle breeds

The canonical coefficients showing the contribution of each measured morphometric trait to the total variation are presented in S4 Table. The first two canonical variates together accounted for 86.26% of the total variation among breeds. The correlation between the cattle breeds and CAN1 was 0.896 and the one between the breeds and CAN2 was 0.771 and the two axes were significant (P<0.001) and sufficient to classify all individual cattle studied. The variables height at withers (WH), rump height (RH), scapulo-ischial length (SIL), horn length (HL), body length (BL), hip width (HW), heart girth (HG), chest depth (CD), ear length (EL), and hock circumference (HC) proved to be the most useful in discriminating among the nine cattle breeds investigated.

**Fig 2:**
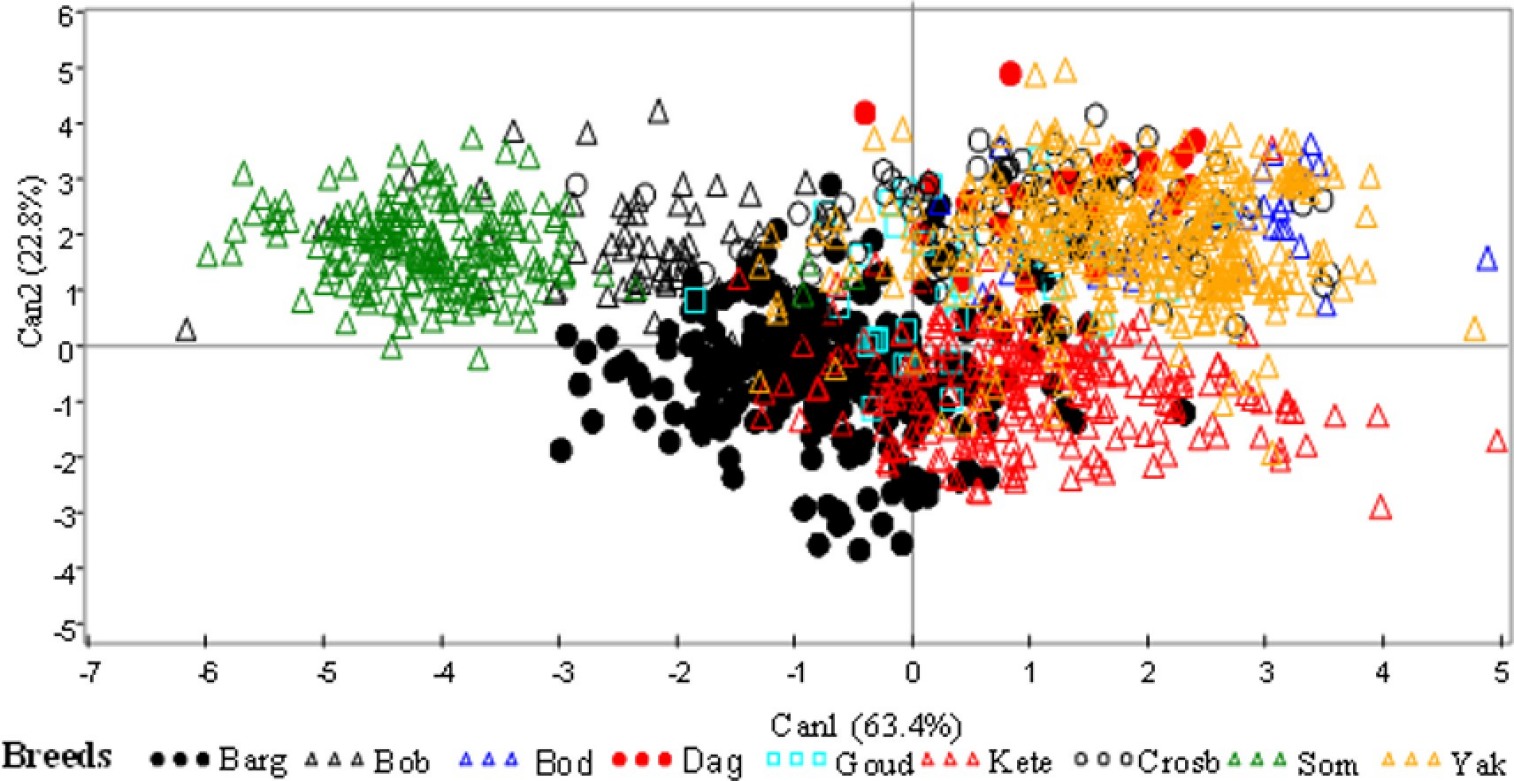
Scatterplot of 1401 individual animals on the first two canonical discriminant functions.

The plot of the centroid values of these first two canonical discriminant functions (Fig 2) shows a clear cut separation between zebu and taurine breeds. The taurine breeds of *Boboji* and Somba appeared to be the two most homogeneous groups whereas *Bargouji* animals partially overlap with the *Keteeji*. Furthermore, there was no clear cut separation among zebu breeds and also between zebus and unnamed crossbreeds.

S5 Table shows the pairwise Mahalanobis distances among the nine cattle breeds investigated. All pairwise distances between the breeds were significant (P<0.001). The greatest distance was observed between Somba and *Bodeeji* (46.87), followed by Somba and *Dageeji* (33.89) whereas the smallest was observed between *Yakanaji* and *Bodeeji* (2.54) followed by *Yakanaji* and crossbreeds (2.59).

**Fig 3:**
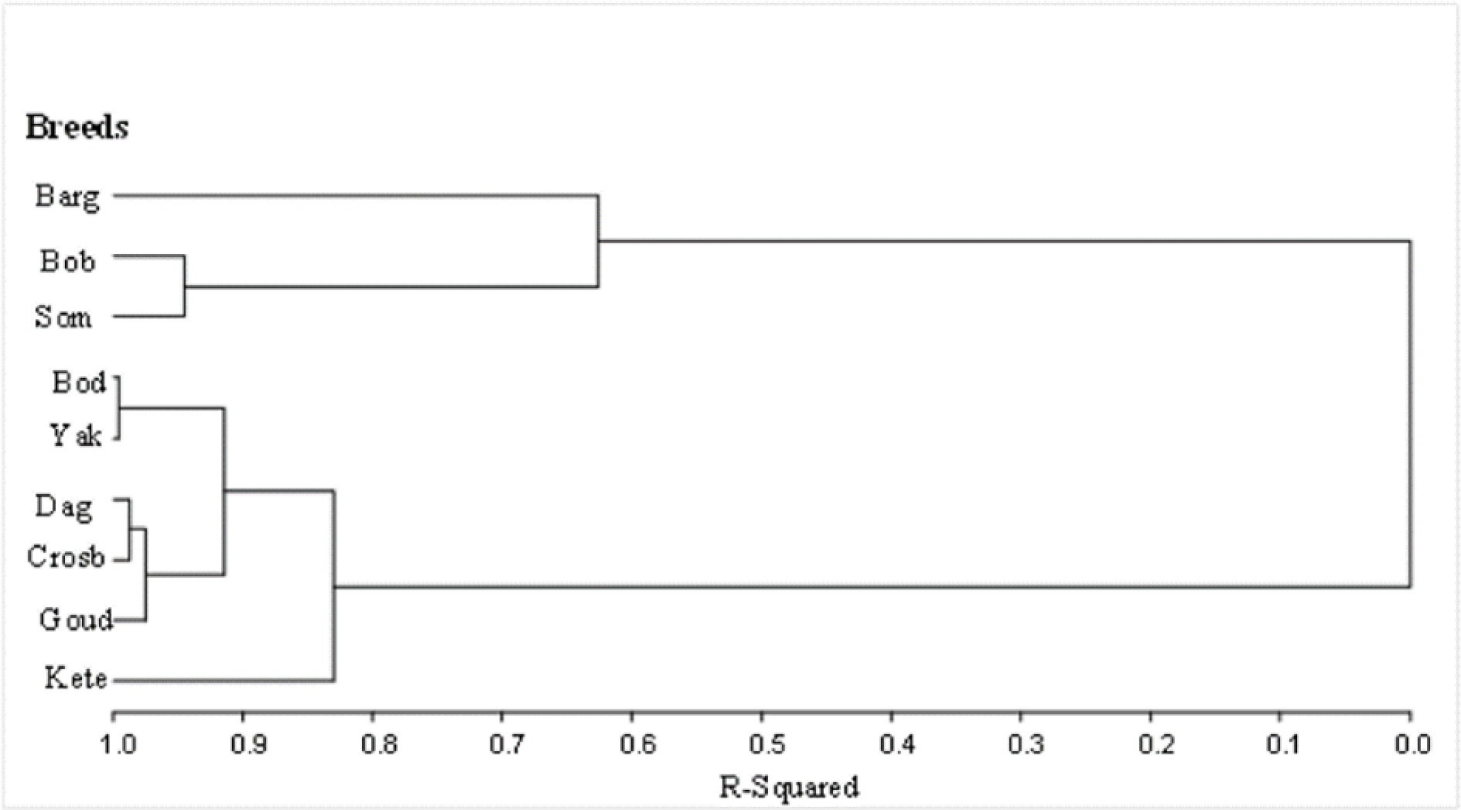
Cluster analysis (UPGMA) of cattle breeds based on Mahalanobis distances.

The dendrogram based on the distance matrix (Fig 3) shows three main clusters: Group 1 included the *Bargouji* breed which was clearly separated from zebu breeds but close to the two taurine breeds of *Somba* and *Boboji* (Cluster 2). The third cluster included all zebu breeds, the unnamed crossbreeds and the *Keteeji*.

The two discriminating functions correctly classified about 75% of the individuals into their a-priori groups (Table 6). However, it is worth noting that about half of the *Yakanaji* and crossbreed individuals were incorrectly classified whereas almost all Somba animals were successfully assigned to their original group.

**Table 6:** Percent (%) of individual cattle classified into their a-priori breeds.

Breed	Posterior probability (%)									Total
	*Bargouji*	*Boboji*	*Bodeeji*	*Dageeji*	*Goudali*	*Keteeji*	Crossbreed	Somba	*Yakanaji*	
***Bargouji***	**80.71**	1.78	0.00	1.19	0.89	11.57	1.48	0.59	1.78	337
***Boboji***	0.00	**84.13**	0.00	0.00	0.00	0.00	4.76	11.11	0.00	63
***Bodeeji***	0.00	0.00	**83.33**	2.78	0.00	0.00	2.78	0.00	11.11	36
***Dageeji***	0.00	0.00	8.33	**75.00**	0.00	0.00	8.33	0.00	8.33	24
***Goudali***	0.00	0.00	0.00	0.00	**84.38**	0.00	6.25	0.00	9.38	32
***Keteeji***	9.96	0.43	1.30	0.00	0.00	**83.55**	2.60	0.00	2.16	231
**Crossbreed**	5.45	11.82	7.27	10.00	5.45	0.00	**49.09**	0.91	10.00	110
**Somba**	0.52	3.11	0.00	0.00	0.00	0.00	0.52	**95.85**	0.00	193
***Yakanaji***	4.27	1.33	14.13	15.20	2.67	4.80	20.00	0.00	**37.60**	375
**Rate**	0.192	0.158	0.166	0.250	0.156	0.164	0.509	0.041	0.624	0.251
**Priors**	0.111	0.111	0.111	0.111	0.111	0.111	0.111	0.111	0.111	0.111

**NB**: The percentage of well classified cow is read on the first diagonal (in bold)

### Correspondence analysis of breeds’ qualitative traits

**Fig 4:**
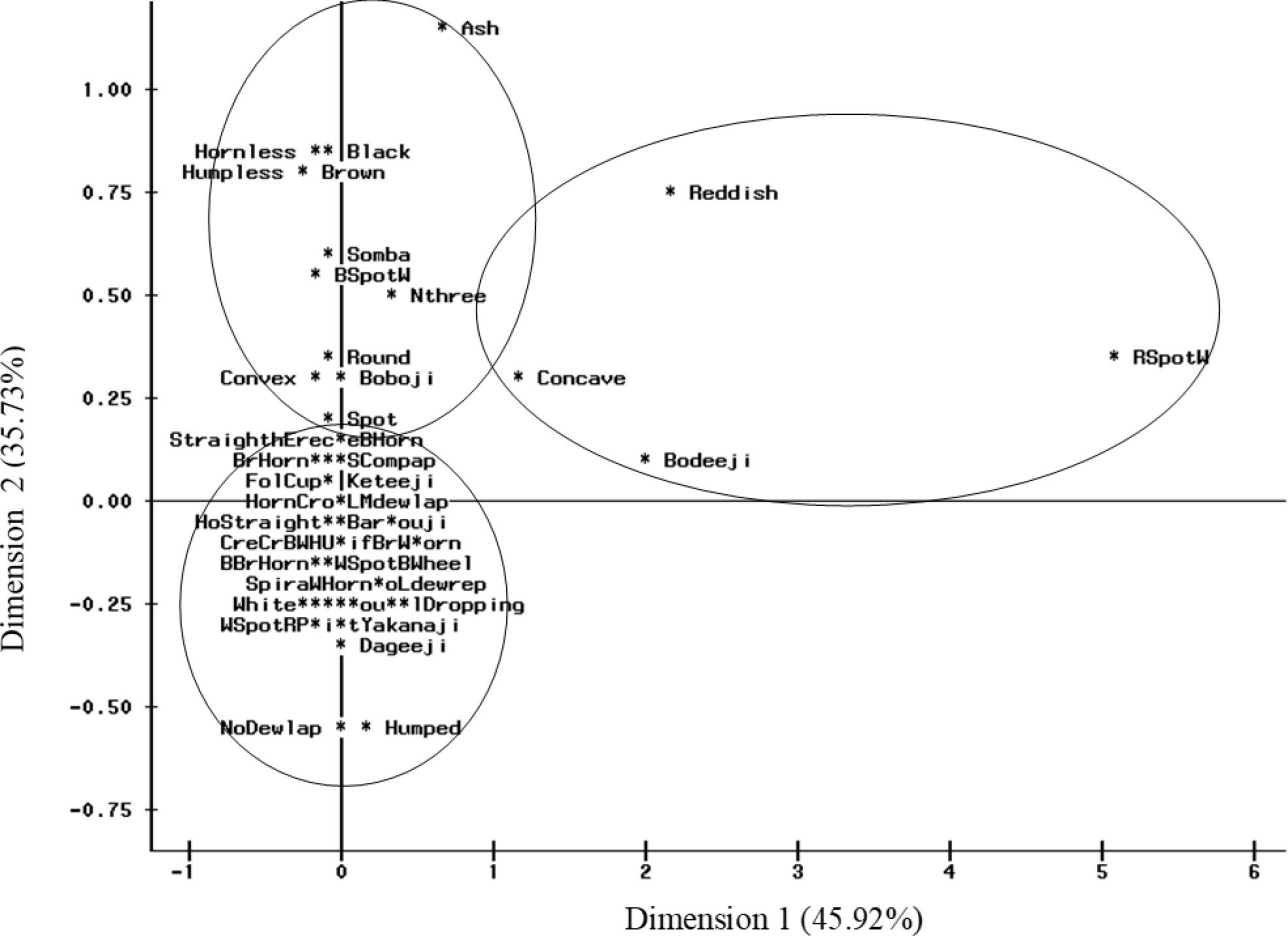
Multiple correspondence analysis of the morphological traits of cattle breeds.

The Multiple Correspondence Analysis (MCA) of the qualitative characters produced slightly different results from those obtained with the Canonical Discriminant Analysis of the quantitative traits (Fig 4). The first dimension separated the taurine breeds (Group I) and the group of *Bodeeji* breed (Group II), from a very heterogeneous group (Group III) which included the other zebu breeds, the unnamed crossbreeds, the *Keteeji* and the *Bargouji* breeds. Group I was characterized by spotted and composed aspects of their coat with dominant black, white or brown colors, cup and numeral three forms of horns and absence of hump whereas Group II was associated with a reddish or red spotted white coat color. The animals of the third group, which is the most heterogeneous, had a dominant white coat color.

## Discussion

The originality and the innovative character of our approach in the West African extensive livestock production systems lie in linking herders’ description of cattle breeds with metric measures of phenotypes. Herders’ accuracy in describing and distinguishing different cattle breeds underlines the relevance of this approach for investigating the phenotypic and genetic variability in farm animal genetic resources. As observed in this study, herders’ classification offers the advantage to be easily comparable to the quantitative classification obtained from the metric measures of phenotypes, as the discriminating criteria used by herders are in agreement with those most commonly used in morphological characterization studies in livestock [25].

There was also a considerable consistency in the local names attributed to each of the identified cattle breeds regardless of the geographic location of the herders. Cattle breeds were mainly named in *Fulfulde* language, the language of *Fulani*/*Fulbe* people, as the majority of pastoralists in West Africa belong to this ethnic group [44]. The breeds names given by cattle herders have previously been reported by many authors and match with the majority of cattle breeds commonly found in West Africa: *Yakanaji* known as *Daneeji/Akuji/Bunaji/*White Fulani [15,44], *Bokoloji/Goudali* or *Zomanta* in *Fongbe* language (the most common native language spoken in Benin) and known as Sokoto Goudali [15,44], *Bodeeji/Bororo* also called *WoDaaBe*, Red Fulani or Red Bororo [44,45], *Djelliji/Djelli/Djalli* known as Peulh Nigérien [46], *Dageeji* also called *Dage* [47], and *Bargouji*/*Bargou* commonly designated as Borgou [48]. Somba and *Keteeji* are also reported in the literature under these same names [48, 49,50,51]. *Boboji* was the only cattle name that does not appear in the existing literature. According to its traits, as described by the herders, it is a cattle of small size, humpless with shorthorns, and reported to be mainly found in the southern regions of Benin. The given characteristics are most similar to those of the shorthorn lagoon taurine cattle, commonly known as Lagunaire or Lagune [52].

The findings of this study reveal the diversity of cattle breeds traditionally raised in Benin and confirm that herders have a good knowledge of their animal genetic resources [53]. The number of breeds reported by the surveyed herders in this study is however greater than those reported in previous studies. For instance, a recent study conducted in northern Benin [53] reported only five cattle breeds (*Keteeji*, *Jaliji/Djelliji*, *Bodeeji*, *Tchiwali/Yakanaji* and Goudali) out of the total of ten recorded in the current study. However, the last authors’ study differs with respect to geographical focus. Indeed, in contrast to our study, which considered seven localities along the geographical north-south gradient in Benin, the investigation by [53] was limited to the Biosphere Reserve of W National Park, in the extreme north of the country. Likewise, the national report to FAO in 2004 [54] also mentioned the presence of eight distinct cattle breeds, omitting the *Dageeji* cattle, which has never been mentioned in previous studies. Main reasons for its neglect may include its small population size and limitation to a few herds in regions not considered in these studies as pastoral areas. Similarly, the Domestic Animal Genetic Resources Information System (DAGRIS), the web-based electronic source of information on selected indigenous farm animal genetic resource [55] reports fewer cattle breeds for Benin than the current study.

Herders’ description of cattle breeds clearly differentiated the humpless small-framed *Boboji* and Somba cattle from the humped and larger-framed zebus. This difference between zebu and taurine cattle subspecies was confirmed by the multivariate analysis performed on the recorded metric traits. Similar results have been obtained through other studies [16,56]. In the same way, the closeness of the *Boboji* and Somba breeds to each other in their physical appearance as mentioned by our respondents have been confirmed by the classical methodologies (AHC and MCA) of classifying farm animal genetic resources based on measured morphometric traits. However, the scatterplot displaying all individual animals on the canonical discriminant functions successfully separated these two breeds, with almost no overlap. This finding is consistent with the results from a previous molecular comparison of the two breeds by [56] and suggests that the morphological characterization may be appropriate and sufficient to study and compare the genetic structure in these two morphologically close breeds.

In contrast, with the exception of the *Bodeeji*, which was separated from others zebu in MCA performed with qualitative traits, the zebu breeds distinguished by herders could not be accurately separated in the multivariate analyses. This underlines the relevance of qualitative information in the morphological description of livestock. Although often considered subjective [16, 57], well-provided information on the qualitative traits of animals could be useful for supplementing quantitative data and may also help in rapidly sampling an animal breed for in-depth studies such as molecular analyses.

The difficulty for a perfect separation of zebu cattle types corroborates the challenges in identifying genetic variation patterns among West African livestock breeds from their morphology as previously reported [17,58]. These authors explain this situation by the lack of selection and high levels of gene flow due to cyclical cross-border cattle herd movements known as “transhumance” and to extensive commercial transactions of cattle on the hoof in the West African region. For these zebu breeds, further discrimination at the molecular level may be necessary.

The Mahalanobis distance obtained between breeds showed, however, a significant variation in the morphological closeness among breeds. The low values of Mahalanobis distances between *Yakanaji* and several other zebu breeds, for instance *Bodeeji* and *Dageeji*, as well as between *Yakanaji* and unnamed crossbreeds reveal a high degree of overlap in morphological characteristics among these breeds. *Yakanaji*, in fact, appeared to be the most heterogeneous zebu breed in the study area as revealed by the low percentage of individuals from this breed correctly classified in their a-priori group. The aforementioned overlap hampers the differentiation of these breeds on the exclusive basis of multivariate analyses of their morphometric traits.

The great heterogeneity of the *Yakanaji* cattle might result from their large use in crossbreeding by livestock herders [52] due to their good production performance and adaptive traits [44,59,60]. Unsupervised and indiscriminate crossbreeding with local cattle breeds, as often encountered, represents an important threat to the conservation and sustainable use of the latter.

In contrast to *Yakanaji*, the highest values of Mahalanobis distances between Goudali and the other zebu cattle breeds are consistent with the specific characteristics of this cattle breed especially its conformation and the absence of horn. Further, this breed is less used in crossbreeding in the surveyed areas. Its sensibility to trypanosomiasis [61,62], an infectious disease caused by a protozoan parasite, could explain its geographical restriction in Benin to the virtually tsetse free Northern Sudanian zone.

Interestingly, there has been a considerable inconsistency in the classification of *Bargouji* and *Keteeji* cattle breeds using multivariate techniques. While the AHC performed on the quantitative traits clearly separated the two cattle types, they were grouped together in the MCA approach using their qualitative traits. In the scatterplot (Fig 2) as well as in the dendogram (Fig 3), individuals from the *Bargouji* breed were very close to the taurine breeds (*Somba and Boboji*) in their morphological characteristics whereas *Keteeji* were grouped with zebus and unnamed crossbred. This important overlap between zebus and *Keteeji* is certainly due to the presence of hump in more than half of the individuals recorded as *Keteeji*. But herders considered the “true” *Bargouji* as a humpless animal; they also firmly identified some humped individuals as *Bargouji* and the “true” *Keteeji* as a humped animal. We can therefore argue that there exist two sub-types (one humped and one humpless) in each of the two cattle breeds. These results, in contrast to those obtained with the analysis of the qualitative traits, are in congruence with herders’ classification who exhibited a very good knowledge of the two cattle breeds. It highlights that neither qualitative nor quantitative traits alone are sufficient in breed characterization, but a good combination of both.

But, both cattle breeds of *Bargouji* and *Keteeji* show body size values intermediate between those of taurine and zebu subspecies which certainly explains the difficulty to distinguish them in previous scientific reports. [48] recognized the existence of both the *Keteeji* and the *Bargouji* (*Barguuji/* Borgou according to the author) in Benin but considered the latter as a taurine and the *Keteeji* as a crossbreed between zebu and taurine. According to [51], who reported its presence since 1918 in the Niger Valley in the Northern Sudanian zone of the country, the *Keteeji* is a “crossbreed between the zebu and the small *N’dama* cattle from the more humid south Borgou”. Yet, the *N’dama* breed, a longhorn taurine cattle native of Guinea, was introduced to Benin only in 1952 [63]. The absence of *N’dama* cattle among the cattle breeds elicited by the herders participating in the present study is consistent with the progressive disappearance of this breed in Benin, previously highlighted [54].

Also, the recent study [53] confounded *Keteeji* to *Borgu* (Borgou). In the official reports on cattle breeds from Benin and West Africa, these two cattle breeds are indiscriminately referred to as “Borgou” cattle, a “stabilized” crossbreed between *Yakanaji* and Somba [64], even though more than half of the Borgou herds were further mated with zebus [65]. One of the issues that emerge from these findings and that has already been pointed out [7,52] is the heterogeneity in many “stabilized” crossbreeds in West Africa. Hence, our results provide support for the hypothesis that *Keteeji* and *Bargouji* are two varieties of the Borgou cattle. It also confirm the necessity of combining molecular analyses, phenotypic characterization and herders’ knowledge for a more accurate differentiation of the breeds and subtypes of cattle raised in extensive African livestock production systems for their effective management and preservation. Several of them have already disappeared before being formally identified [12].

## Conclusions

The aim of this study was to validate pastoralists’ classification system and knowledge of cattle breeds with quantitative morphometric analyses. The results showed the need to associate qualitative traits and quantitative traits measurement in morphological discrimination of cattle breeds. The findings reveal that livestock herders have a good knowledge of the morphological trait characteristics of the cattle breeds raised in their herds. The multivariate analyses of the morphometric traits showed less accuracy than herders’ classification approach in discriminating most of the zebu breeds because of high variability within and among breeds. The difficulty of perfect separation of these cattle breeds, whatever the single approach of classification, suggests combining livestock herders’ traditional knowledge with phenotypic and molecular genetic approaches as an integrated tool for the appropriate characterization of farm animal genetic resources.

### Ethics approval and consent to participate

The study involved taking body measurements from cattle with the consent and in the presence of the cattle herder. There is no specific legislation for body measurements and hence no approval was necessary. All the data was collected in traditional farms and the animal owners agreed to be involved in the project through the Communal Sector for Agricultural Development (SCDA), which is the decentralized institution for the management of the agricultural sector in the surveyed municipalities. All the animals included in this study were managed in accordance with the criteria for the assessment of animal welfare identified by the Welfare Quality Project (WQP) [42].

## Supporting information

S1 File (XLSX)

S1 Table Least square means in cm (± standard error) of morphological variables for nine cattle breeds raised in Benin (pooled data for both cows and bulls). (PDF)

S2 Table Distribution (in %) of measured qualitative traits among nine cattle breeds raised in Benin. (PDF)

S3 Table Distribution (in %) of measured qualitative traits among nine cattle breeds raised in Benin (continued). (PDF)

S4 Table Canonical loadings of fifteen measured morphological traits from nine cattle breeds raised in Benin on the first two canonical variables. (PDF)

S5 Table Pairwise Squared Mahalanobis distances among nine cattle breeds raised in Benin (based on data measured from both cows and bulls). (PDF)

S1 Fig Somba cow. (TIF)

S2 Fig *Boboji*/Lagunaire cow. (TIF)

S3 Fig *Yakanaji/Bunaji*/White Fulani cow. (TIF)

S4 Fig *Goudali/ Bokoloji* /Sokoto Gudali cow. (TIF)

S5 Fig *Bodeeji/ Bororo/* Red Bororo cow. (TIF)

S6 Fig *Djelliji/Djalli*/Peulh Nigérien bull. (TIF)

S7 Fig *Dageeji/Dage* cow. (TIF)

S8 Fig *Bargouji / Keteeji*/Borgou cow. (TIF)

## References

[1] Belemsaga D, Lombo Y, Thevenon S, Sylla S. Inventory Analysis of West African Cattle Breeds In: Makkar HP, Viljoen GJ. (eds) Applications of Gene-Based Technologies for Improving Animal Production and Health in Developing Countries. Springer, Dordrecht; 2005 10.1007/1-4020-3312-5_15

[2] Mwai O, Hanotte O, Kwon YJ, Cho S. African indigenous cattle: unique genetic resources in a rapidly changing world. Asian Australas J. Anim. Sci. 2015; 28(7), 911–921. 10.5713/ajas.15.0002R PMC4478499 26104394

[3] Dueppen SA. Cattle in the West African savanna: evidence from 1st millennium CE Kirikongo, Burkina Faso. J. Archaeol. Sci. 2012; 39(1), 92–101. 10.1016/j.jas.2011.09.005

[4] Hanotte O, Dessie T, Kempe S. Time to Tap Africa’s Livestock Genomes. Science. 2010; 328, 1640 10.1126/science.1186254 20576875

[5] Hanotte O, Bradley DG, Ochieng JW, Verjee Y, Hill EW, Rege JEO. African pastoralism: genetic imprints of origins and migrations. Science. 2002; 296(5566), 336–339. 10.1126/science.1069878 11951043

[6] Freeman AR, Meghen CM, Mac Hugh DE, Loftus RT, Achukwi MD, Bado A, et al Admixture and diversity in West African cattle populations. Mol. Ecol. 2004; 13(11), 3477–3487. 10.1111/j.1365-294X.2004.02311.x 15488005

[7] Flori L, Thevenon S, Dayo G K, Senou M, Sylla S, Berthier D, et al Adaptive admixture in the West African bovine hybrid zone: insight from the Borgou population. Mol. Ecol. 2014; 23, 3241–3257. 10.1111/mec.12816 24888437

[8] Hoffmann I. Climate change and the characterization, breeding and conservation of animal genetic resources. Anim. Genet. 2010; 41(s1), 32–46. 10.1111/j.1365-2052.2010.02043.x 20500754

[9] Rege JEO, Marshall K, Notenbaert A, Ojango JMK, Okeyo AM. Pro-poor animal improvement and breeding–what can science do? Lives. Sci. 2011; 136, 15–28. 10.1016/j.livsci.2010.09.003

[10] Bruford MW, Ginja C, Hoffmann I, Joost S, Orozco-terWengel P, Alberto FJ, et al Prospects and challenges for the conservation of farm animal genomic resources, 2015–2025. Front. Genet. 2015; 6, 314 10.3389/fgene.2015.00314 PMC4612686 26539210

[11] Sunderland TC. Food security: why is biodiversity important?. Int. Forest. Rev. 2011; 13(3), 265–274. 10.1505/146554811798293908

[12] FAO. The second report on the state of the world's animal genetic resources for food and agriculture, edited by Scherf BD, Pilling D. FAO Commission on Genetic Resources for Food and Agriculture Assessments. Rome, Italy; 2015. [cited 2018 Nov 12] available from: http://www.fao.org/3/a-i4787e.pdf

[13] Sokouri DP, Loukou NE, Yapi-Gnaoré CV, Mondeil F, Gnangbe F. Caractérisation phénotypique des bovins à viande (Bos taurus et Bos indicus) au centre (Bouaké) et au nord (Korhogo) de la Côte d’Ivoire. AGRI. 2007; 40, 43–53. 10.1017/S1014233900002182

[14] N'goran KE, Yapi Gnaoré CV, Fantodji TA, N'goran A. Caractérisation phénotypique et performances des Vaches laitières de trois régions de Côte d'Ivoire. Arch. Zootec. 2008; 57 (220), 415–426. [cited 2018 Jun 17] available from: http://www.redalyc.org/articulo.oa?id=49515034003

[15] Yakubu A, Idahor KO, Haruna H, Wheto M, Amusan S. Multivariate analysis of phenotypic differentiation in bunaji and Sokoto gudali cattle. Acta. Agric. Slov. 2010; 96 (2), 75–80. 10.2478/v10014-010-0018-9

[16] Traoré A, Koudandé DO, Fernandez I, Soudré A, Granda V, Alvarez I, et al Geographical assessment of body measurements and qualitative traits in West African cattle. Trop. Anim. Health Prod. 2015; 47(8), 1505–1513. 10.1007/s11250-015-0891-7 26224600

[17] Álvarez I, Traoré A, Fernández I, Lecomte T, Soudré A, Kaboré A, et al Assessing introgression of Sahelian zebu genes into native *Bos taurus* breeds in Burkina Faso. Mol. Biol. Rep. 2014; 41, 3745–3754. 10.1007/s11033-014-3239-x 24532141

[18] Ndiaye NP, Sow A, Dayo GK, Ndiaye S, Sawadogo GJ, Sembène M. Genetic diversity and phylogenetic relationships in local cattle breeds of Senegal based on autosomal microsatellite markers. Vet world. 2015; 8(8), 994 10.14202/vetworld.2015.994-1005 27047188 PMC4774767

[19] Álvarez I, Pérez Pardal L, Traoré A, Koudandé DO, Fernández I, Soudré A, et al Differences in genetic structure assessed using Y‐chromosome and mitochondrial DNA markers do not shape the contributions to diversity in African sires. J Anim Breed Genet. 2017; 134(5), 393–404. 10.1111/jbg.12278 28464302

[20] Grema M, Traoré A, Issa M, Hamani M, Abdou M, Soudré A, et al Short tandem repeat (STR) based genetic diversity and relationship of indigenous Niger cattle. Arch Tierzucht. 2017a; 60(4), 399–408.

[21] Kim SJ, Ka S, Ha JW, Kim J, Yoo D, Kim K, et al Cattle genome-wide analysis reveals genetic signatures in trypanotolerant N’Dama. BMC genomics. 2017; 18(1), 371 10.1186/s12864-017-3742-2 28499406 PMC5427609

[22] Jabbar MA, Swallow BM, Rege JEO. Incorporation of farmer knowledge and preferences in designing breeding policy and conservation strategy for domestic animals. Outlook Agr. 1999; 28(4), 239–243. 10.1007/s11250-005-4253-8

[23] Delgado JV, Barba C, Camacho ME, Sereno FTPS, Martinez A, Vega-Pla JL. Livestock characterisation in Spain. AGRI. 2001; 29, 7–18. 10.1017/S1014233900005162

[24] Lanari MR, Taddeo H, Domingo E, Centeno MP, Gallo L. Phenotypic differentiation of exterior traits in local Criollo goat population in Patagonia (Argentina). Archiv. Tierzucht. Dummerstorf. 2003; 46, 347–356. 10.5194/aab-46-347-2003

[25] FAO. Phenotypic Characterization of Animal Genetic Resources. Animal Production and Health Guidelines 2012a; N° 11, FAO, Rome, Italy [cited 2018 Jun 12] available from: http://www.fao.org/3/i2686e/i2686e00.pdf

[26] Dossa LH, Wollny C, Gauly M. Spatial variation in goat populations from Benin as revealed by multivariate analysis of morphological traits. Small Rumin. Res. 2007; 73, 150–159. 10.1016/j.smallrumres.2007.01.003

[27] Ayantunde A A, Duncan AJ, van Wijk MT, Thorne P. Role of herbivores in sustainable agriculture in Sub-Saharan Africa. Animal, 2018; 12(s2), 199–209. 10.1017/S175173111800174X 30139396

[28] Boutrais J. The Fulani and cattle breeds: crossbreeding and heritage strategies. Africa 2007; 77(1), 18–36. 10.3366/afr.2007.77.1.18

[29] Krätli S. Cattle breeding, complexity and mobility in a structurally unpredictable environment: the WoDaaBe herders of Niger. Nomadic Peoples. 2008; 12(1), 11–41. 10.3167/np.2008.120102

[30] Holloway L, Morris C, Gilna B, Gibbs D. Choosing and rejecting cattle and sheep: changing discourses and practices of (de) selection in pedigree livestock breeding. Agr. Hum. Values. 2011; 28(4), 533–547. 10.1007/s10460-010-9298-2

[31] Kugonza DR, Nabasirye M, Hanotte O, Mpairwe D, Okeyo AM. Pastoralists' indigenous selection criteria and other breeding practices of the long-horned Ankole cattle in Uganda. Trop. Anim. Health Prod. 2012; 44(3), 557–565. 10.1007/s11250-011-9935-9 21805229

[32] Brown T. The N'Dama Dilemma: Ethnogenetics and Small Ruminant Breed Dynamics in the Tsetse Zone, the Gambia. Outlook Agri. 2015; 44(3), 173–178. 10.5367/oa.2015.0210

[33] Yakubu A, Ogah DM, Idahor KO. Principal component analysis of the morphostructural indices of White Fulani cattle. Trakia J. Sci. 2009; 7, 67–73. [cited 2018 Jun 12] available from: http://tru.uni-sz.bg/tsj/vol7no2_2009/new_yakubu.pdf

[34] Okpeku M, Yakubu A, Peters S, Ozoje M, Ikeobi C, Adebambo O, et al Application of multivariate principal component analysis to morphological characterization of indigenous goats in Southern Nigeria. Acta Agric. Slov. 2011; 98(2), 101 10.2478/v10014-011-0026-4

[35] Traoré A, Tamboura HH, Kaboré A, Royo LJ, Fernández I, Álvarez I, et al Multivariate characterization of morphological traits in Burkina Faso sheep. Small Rumin. Res. 2008; 80, 62–67. 10.1016/j.smallrumres.2008.09.011

[36] Yakubu A, Akinyemi MO. An evaluation of sexual size dimorphism in Uda sheep using multifactorial discriminant analysis. Acta. Agr. Scand. A-An. 2010; 60(2), 74–78. 10.1080/09064702.2010.502242

[37] Birteeb PT, Peters SO, Yakubu A, Adeleke MA, Ozoje MO. Multivariate characterisation of the phenotypic traits of Djallonke and Sahel sheep in Northern Ghana. Trop. Anim. Health Prod. 2012; 45(1), 267–274. 10.1007/s11250-012-0211-4 22710941

[38] Kefena E, Dessie T, Han JL, Kurtu MY, Rosenbom S, Beja-Pereira A. Morphological diversities and ecozones of Ethiopian horse populations. Anim. Genet. Resour. 2012; 50, 1–12. 10.1017/S2078633612000021

[39] Akoègninou A, Van der Burg WJ, Van Der Masen LJG. Flore analytique du Bénin. Wageningen: éditions Backhuys Publishers; 2006.

[40] INSAE (Institut National de la Statistique et de l'Analyse Economique). RGPH4 : Que retenir des effectifs de population en 2013; 2015 [cited 2018 Nov 17] available from: https://www.insae-bj.org/images/docs/insae-statistiques/demographiques/population/Resultats%20definitifs%20RGPH4.pdf

[41] FAOSTAT, 2017. Country STAT-Benin/Production/ Répartition des effectifs d'animaux vivants par année, Niveau administratif 2. [cited 2018 Nov 30] available from: http://193.43.36.162/home.aspx?c=BEN&ta=053SPD135&tr=21

[42] Vapnek J, Chapman M S. Legislative and regulatory options for animal welfare. FAO Legislative Studies No. 104. 2010. [cited 2019 Apr 19] available from: http://www.fao.org/3/i1907e/i1907e01.pdf

[43] Galal S, Boyazoglu J, Hammond K, Leitch H, Scherf B D, Galal S. et al (2001). Preparation of the first report on the state of the world’s animal genetic resources: Guidelines for the development of country reports Food and Agriculture Organization of the United Nations (FAO), Rome, Italy [cited 2019 Jul 14] available from: http://www.fao.org/3/a-al767e.pdf

[44] Blench R. Traditional livestock breeds: Geographical distribution and dynamics in relation to the ecology of West Africa (Vol. 67) London, UK: Overseas Development Institute 1999 [cited 2018 Dec 15] available from: https://www.odi.org/resources/docs/2766

[45] Boutrais J. Zébus, mouches tsé-tsé et techniciens en Adamaoua camerounais. In Robineau C. (Ed. SC.), Les terrains du développement; approches pluri-disciplinaires des économies du Sud, Paris, Orstom, Didactiques, 1992; 235–252. [cited 2018 Dec 04] available from: http://horizon.documentation.ird.fr/exl-doc/pleins_textes/divers07/37475.pdf

[46] Belli P, Turini J, Harouna A, Garba IA, Pistocchini E, Zecchini M. Critères de sélection des bovins laitiers par les éleveurs autour de Niamey au Niger. Rev. Élev. méd. vét. pays trop. 2008; 61(1), 51–56.

[47] Eruola AO, Sholademi AA, Ufoegbune GC, Makinde AA, Iposu SO. Interactive effects of season, breed and grazing location on lactation yield in a tropical wet-and–dry climate of Nigeria. World J. Agric. Sci. 2013; 1(11), 318–322. http://wsrjournals.org/journal/wjas

[48] Djedjebi TS. Pastoralistes et la ville au Bénin: Livelihoods en questionnement Doctorate thesis. University of Leiden, The Netherlands; 2009.

[49] Dossa LH, Vanvanhossou FUS. The indigenous Somba cattle of the hilly Atacora region in North-West Benin: threats and opportunities for its sustainable use. Trop. Anim. Health Prod. 2016; 48(2), 349–359. 10.1007/s11250-015-0958-5 26590609

[50] Hall SJ, Gnaho LK, Meghen C. Une enquête sur la race bovine Somba au Bénin. Rev. élev. méd. vét. pays trop. 1995; 48(1), 77–83.

[51] Van Driel A. Sharing a Valley The changing relations between agriculturalists and pastoralists in the Niger Valley of Benin. Research Report no 64 Leiden: African Studies Centre, Leiden University, Leiden, The Netherlands; 2002.

[52] Aboagye GS, Tawah CL, Rege JEO. Shorthorn cattle of West and Central Africa. III. Physical, adaptative and special genetic characteristics. World Anim. Rev. 1994; 78: 22–32. [cited 2018 Nov 14] available from: www.fao.org/docrep/T1300T/t1300t0a.htm

[53] Tamou C, de Boer IJ, Ripoll-Bosch R, Oosting SJ. Understanding roles and functions of cattle breeds for pastoralists in Benin. Lives. Sci. 2018; 210, 129–136. 10.1016/j.livsci.2018.02.013

[54] DE-MAEP (Direction de l’Elevage). Rapport annuel 2003. MAEP, Cotonou Bénin; 2004. [cited 2018 Jan 17] available from: http://www.fao.org/AG/againfo/programmes/en/genetics/documents/Interlaken/countryreports/Benin.pdf

[55] DAGRIS (Domestic Animal Genetic Resources Information System). Breeds in Benin; 2018. [cited 2018 Jul 18] available from: http://dagris.info/countries/152/breeds

[56] Moazami-Goudarzi K, Belemsaga DMA, Ceriotti G, Laloë D, Fagbohoun F, Kouagou N’ T, et al Caractérisation de la race bovine Somba à l’aide de marqueurs moléculaires. Rev. élev. méd. vét. pays trop. 2001; 54 (2), 129–138. https://agritrop.cirad.fr/489759/1/ID489759.pdf

[57] Desta TT, Ayalew W, Hedge BP. Breed and trait preferences of Sheko cattle keeper in southern Ethiopia. Trop. Anim. Health Prod. 2011; 43, 851–856. 10.1007/s11250-010-9772-2 21181495

[58] Traoré A, Álvarez I, Fernández I, Pérez-Pardal L, Kaboré A, Ouédraogo-Sanou GMS, et al Ascertaining gene flow patterns in livestock populations of developing countries: a case study in Burkina Faso goat. BMC Genet. 2012; 13(1), 35 10.1186/1471-2156-13-35 22564289 PMC3413537

[59] Alphonsus C, Akpa GN, Barje PP, Finangwai HI, Adamu BD. Comparative evaluation of linear udder and body conformation traits of bunaji and Friesian x bunaji cows. World J. Life Sci. Med. 2012; 2 (4), 134–140. http://rrpjournals.org/wjlsmr/en_wjlsmr_vol_2_iss_4_pg_134_140.pdf

[60] Pelican M. Friendship among pastoral fulbe in northwest Cameroon. Afr. Study Monogr. 2012; 33(3). 165–188. 10.14989/159427

[61] Ogunsanmi AO, Ikede BO, Akpavie SO. Effects of management, season, vegetation zone and breed on the prevalence of bovine trypanosomiasis in South Western Nigeria. Isr. J. Vet. Med. 2000; 55(2), 1−7. 10.4236/oalib.1101521

[62] Talabi AO, Otesile EB, Joshua RA, Oladosu LA. Clinical observations on three Nigerian Zebu cattle breeds following experimental Trypanosoma congolense infection. Bull. Anim. Hlth. Prod. Afr. 2012; 60 (2), 187−192.

[63] Youssao AKI, Ahissou A, Toure Z. Introduction de la race bovine N’Dama à la Ferme Elevage de l’Okpara au Bénin. Quelques performances zootechniques. Anim. Genet. Resour. 2000; 27, 17–25. 10.1017/S1014233900001255

[64] Porter V. A world dictionary of livestock breeds, types and varieties 5th ed. CABI Publishing Wallingford 2002.

[65] Porter V, Alderson L, Hall S, Sponenberg DP. Mason’s World Encyclopedia of Livestock Breeds and Breeding. 1st ed. CABI Publishing Wallingford; 2016.

