# Supplementary material for: Confronting pastoralists’ knowledge of cattle breeds raised in the extensive production systems of Benin with multivariate analyses of morphological traits
Source: PLoS One. 2019 Sep 26;14(9):e0222756. doi: 10.1371/journal.pone.0222756 (PMC6762103; doi:10.1371/journal.pone.0222756)
Supplement: S1 Table — (PDF) [file pone.0222756.s002.pdf]

S1_Table. Least square means in cm (± standard error) of morphological variables for nine cattle breeds raised in Benin (pooled data for both cows and bulls).

Trait	Cattle breeds								
	Bargouji (n=337)	Boboji (n=63)	Bodeeji (n=36)	Dageeji (n=24)	Goudali (n=32)	Keteeji (n=231)	Crossbreed (n=110)	Somba (n=193)	Yakanaji (n=375)
MC	41.7^{de} ± 0.27	43.2^{cd}±0.32	45.8^{ab}± 0.21	44.2^{bc}± 0.50	41.9^{de}±0.28	46.5^{a}±1.51	44.2^{bc}±0.65	41.2^{e}±0.32	45.1^{ab}±0.30
HW	19.7^{bc}± 0.11	19.2^{c}±0.16	20.5^{b}±0.10	19.3^{c}± 0.21	20.9^{b}±0.05	22.8^{a}±0.66	20.7^{b}±0.18	17.7^{d}±0.16	20.8^{b}±0.15
FAL	47.1^{b}±0.59	44.0^{c}±0.41	48.5^{b}±0.21	47.6^{b}± 0.59	46.7^{b}±0.42	51.0^{a}±0.47	47.4^{b}±0.41	40.0^{d}± 0.41	47.4^{b}±0.3
EL	19.7^{d}±0.25	17.4^{e}±0.13	20.7^{c}± 0.17	19.0^{d}± 0.25	21.9^{b}±0.18	22.8^{a}±0.48	20.5^{c}±0.29	15.6^{f}±0.13	20.9^{c}±0.11
HL	36.1^{d}± 0.49	28.2^{e}±1.46	62.2^{a}±1.29	49.5^{b}± 0.49	14.7^{f}±1.93	45.5^{c}±2.72	42.1^{c}±3.03	16.4^{f}±1.46	52.8^{b}±0.58
HG	153.8^{d}±1.72	153.1^{d}±1.42	174.1^{a}±0.77	169.3^{ab}±1.72	174.4^{a}±1.15	160.2^{c}±2.81	167.6^{b}±2.39	143.4^{e}±1.42	171.9^{ab}±0.65
HC	35.2^{f}±0.45	39.5^{d}±0.54	44.9^{b}±0.76	47.5^{a}±0.45	42.7^{c}±0.95	38.3^{d}±1.30	45.2^{b}±1.27	36.8^{e}±0.54	43.9^{bc}±0.62
TL	100.1^{ab}± 0.87	87.1^{c}±0.74	104.5^{ab}±0.85	97.6^{abc}± 0.87	108.4^{a}± 0.57	104.7^{ab}±1.91	109.8^{a}±1.81	92.6^{bc}±0.74	104.2^{ab}±0.52
SPW	34.8^{b}±0.39	28.2^{d}±0.43	33.3^{b}±0.52	30.5^{c}± 0.39	34.3^{b}±0.42	37.9^{a}±0.84	32.7^{b}±0.70	28.8^{d}±0.43	34.5^{b}±0.24
HW	42.6^{c}±0.35	40.3^{d}±0.37	45.0^{ab}±0.52	44.3^{b}± 0.35	45.7^{ab}±0.25	44.5^{b}±1.80	44.4^{b}±0.52	36.5^{e}±0.37	46.3^{a}±0.18
CD	63.5^{a}±0.44	54.8^{c}±0.89	64.1^{a}±0.67	64.7^{a}±0.44	65.1^{a}± 0.4	66.1^{a}±1.16	61.2^{b}±1.06	50.9^{d}±0.89	66.2^{a}±0.51
WH	116.4^{c}±1.13	110.2^{d}±0.76	132.7^{a}± 0.47	129.1^{a}± 1.13	131.8^{a}±0.93	130.1^{a}±0.97	124.4^{b}±1.76	101.1^{e}±0.36	132.3^{a}±0.76
RH	119.8^{c}±1.15	113.6^{d}±0.76	133.0^{a}±0.39	128.8^{b}±1.15	132.7^{a}±0.94	130.7^{a}±1.00	128.4^{b}±1.75	106.9^{e}±0.76	133.3^{a}±0.37
BL	117.3^{d}±1.30	114.8^{d}±1.26	134.0^{a}±0.96	124.3^{bc}±1.30	124.3^{bc}±0.64	127.0^{b}±3.10	122.1^{c}±1.58	101.9^{e}±1.26	127.1^{b}±0.66
SIL	164.1^{e}±1.12	152.3^{f}±1.70	194.6^{a}± 0.90	180.4^{c}±1.12	174.2^{cd}±1.46	174.5^{d}±3.15	177.1^{cd}± 3.1	143.1^{g}±1.70	184.8^{b}±1.39

^{abc} Means with different letters in rows are significantly different at P ≤ 0.001; SNK's multiple mean comparison test;

MC: Muzzle circumference, HW: Head width, FAL: Face length, EL: Ear length, HL: Horn length, HG: Heart Girth, HC: Hock circumference, TL: Tail length, SPW: Shoulder point width, HW: Hip Width, CD: Chest depth, WH: Withers height, RH: Rump height, BL: Body length, SIL: Scapula-ischial length
