# Supplementary material for: Confronting pastoralists’ knowledge of cattle breeds raised in the extensive production systems of Benin with multivariate analyses of morphological traits
Source: PLoS One. 2019 Sep 26;14(9):e0222756. doi: 10.1371/journal.pone.0222756 (PMC6762103; doi:10.1371/journal.pone.0222756)
Supplement: S2 Table — (PDF) [file pone.0222756.s003.pdf]

S2_table. Distribution (in %) of measured qualitative traits among nine cattle breeds raised in Benin

Qualitative trait	Definition	Breeds									Total
		Bargouji (n=337)	Boboji (n=63)	Bodeeji (n=36)	Dageeji (n=24)	Goudali (n=32)	Keteeji (n=231)	Crossbreed (n=110)	Somba (n=193)	Yakanaji (n=375)	
General aspect of the coat											
	Uniform	67.7	54.0	73.1	87.5	75.0	58.9	70.0	62.2	71.2	66.5
	Spotted	8.6	27.0	3.8	8.3	0.0	9.1	3.6	13.0	10.4	9.9
	Composed	23.7	19.0	23.1	4.2	25.0	32.0	26.4	24.9	18.4	23.6
Unique color of coat											
	Black	6.1	17.6	5.5	0.0	4.2	14.0	11.7	43.3	6.4	12.9
	White	91.7	76.5	10.5	100.0	95.8	81.6	77.9	28.3	90.6	78.6
	Dark red	0.4	5.9	68.4	0.0	0.0	2.2	7.8	10.8	0.7	4.3
	Brown	1.3	0.0	0.0	0.0	0.0	0.7	1.3	3.3	0.4	1.1
	Fawn	0.4	0.0	15.8	0.0	0.0	1.5	1.3	14.2	1.9	3.1
Other color of coat											
	White spotted black	64.0	61.9	0.0	100.0	100.0	71.0	73.1	46.2	71.7	67.3
	Black spotted white	17.3	28.6	0.0	0.0	0.0	24.2	15.4	50.0	14.1	20.5
	White spotted red	17.3	4.8	0.0	0.0	0.0	4.8	11.5	3.8	14.1	10.9
	Red spotted white	1.3	4.8	100.0	0.0	0.0	0.0	0.0	0.0	0.0	1.3
Cephalic profile											
	Concave	0.3	4.8	15.4	4.2	0.0	0.0	1.8	3.6	2.4	1.9
	Convex	4.4	7.9	0.0	0.0	3.1	10.8	14.5	13.5	5.9	7.9
	Straight	95.3	87.3	84.6	95.8	96.9	89.2	83.6	82.9	91.7	90.2
Presence of hump											
	Absent	45.3	100.0	0.0	0.0	0.0	40.3	45.5	100.0	0.0	39.7
	Present	54.7	0.0	100.0	100.0	100.0	59.7	54.5	0.0	100.0	60.3
Presence of horn											
	Absent	0.0	0.0	0.0	0.0	9.4	0.0	0.9	2.6	0.0	0.6
	Present	100.0	100.0	100.0	100.0	90.6	100.0	99.1	97.6	100.0	99.4
Color of horn											
	Black	27.2	38.1	40.0	37.5	36.7	29.6	33.9	51.9	32.8	34.1
	Brown	8.0	14.3	4.0	8.3	16.7	9.1	9.2	10.1	7.1	8.7
	White	15.1	9.5	12.0	33.3	13.3	18.3	8.3	7.4	17.8	14.7
	Black and brown	8.9	3.2	4.0	0.0	3.3	6.5	2.8	2.1	4.6	5.3
	Black and white	28.7	30.2	28.0	16.7	30.0	21.3	39.4	26.5	33.9	29.3
	Brown and white	4.4	4.8	12.0	4.2	0.0	5.7	3.7	1.1	2.2	3.6
	Black, brown and white	7.7	0.0	0.0	0.0	0.0	9.6	2.8	1.1	1.6	4.3

^{abc} Means with different letters in rows are significantly different at $P \leq 0.001$; (Chi-square test)
