# Supplementary material for: Confronting pastoralists’ knowledge of cattle breeds raised in the extensive production systems of Benin with multivariate analyses of morphological traits
Source: PLoS One. 2019 Sep 26;14(9):e0222756. doi: 10.1371/journal.pone.0222756 (PMC6762103; doi:10.1371/journal.pone.0222756)
Supplement: S3 Table — (PDF) [file pone.0222756.s004.pdf]

S3_table. Distribution (in %) of measured qualitative traits among nine cattle breeds raised in Benin (continued).

Qualitative trait	Definition	Breeds									Total
		Bargouji (n=337)	Boboji (n=63)	Bodeeji (n=36)	Dageeji (n=24)	Goudali (n=32)	Keteeji (n=231)	Crossbreed (n=110)	Somba (n=193)	Yakanaji (n=375)	
Horn shape											
	Straight	22.8	12.7	16.0	20.8	16.7	17.8	11.0	19.6	10.1	16.4
	Crown	2.1	6.3	4.0	4.2	3.3	2.6	3.7	3.7	5.5	3.7
	Cup	20.4	22.2	12.0	8.3	23.3	32.6	26.6	21.2	14.8	21.3
	Folded back cup	7.4	0.0	0.0	0.0	3.3	7.4	5.5	5.3	2.7	5.0
	Crescent	20.4	22.2	16.0	20.8	20.0	10.4	22.9	20.1	27.6	20.8
	Lyre	20.4	27.0	28.0	33.3	33.3	26.5	20.2	24.3	28.4	25.0
	Folded back lyre	4.4	9.5	16.0	12.5	0.0	0.9	9.2	3.2	9.3	5.8
	Wheel	0.6	0.0	4.0	0.0	0.0	0.9	0.0	0.5	0.8	0.7
	Spiral	0.3	0.0	0.0	0.0	0.0	0.9	0.0	0.0	0.3	0.3
	Numeral three	1.2	0.0	4.0	0.0	0.0	0.0	0.9	2.1	0.5	0.9
Ear shape											
	Rounded	46.2	41.3	19.2	25.0	40.6	57.6	24.5	72.0	22.1	42.2
	Pointed	53.8	58.7	80.8	75.0	59.4	42.4	75.5	28.0	77.9	57.8
Orientation of ear											
	Erected	41.4	34.9	19.2	45.8	46.9	12.1	20.0	45.6	29.1	31.6
	Horizontal	57.4	63.5	73.1	54.2	46.9	86.6	79.1	52.3	66.9	66.1
	Dropping	1.2	1.6	7.7	0.0	6.2	1.3	0.9	2.1	4.0	2.3
Dewlap size											
	Absent	5.0	0.0	3.8	4.2	3.1	0.9	0.9	0.0	4.3	2.8
	Small	47.6	30.2	26.9	16.7	37.5	43.3	40.0	51.8	38.0	42.3
	Medium	39.6	58.7	38.5	75.0	40.6	49.8	40.0	39.9	39.6	42.8
	Large	7.7	11.1	30.8	4.2	18.8	6.1	19.1	8.3	18.2	12.0

[abc] Means with different letters in rows are significantly different at $P \leq 0.001$; (Chi-square test)
