# Supplementary material for: Confronting pastoralists’ knowledge of cattle breeds raised in the extensive production systems of Benin with multivariate analyses of morphological traits
Source: PLoS One. 2019 Sep 26;14(9):e0222756. doi: 10.1371/journal.pone.0222756 (PMC6762103; doi:10.1371/journal.pone.0222756)
Supplement: S4 Table — (PDF) [file pone.0222756.s005.pdf]

S4_table. Canonical loadings of fifteen measured morphological traits from nine cattle breeds raised in Benin on the first two canonical variables

**Trait**	Canonical variate	
	Can1	Can2
MC	0.421	0.084
HW	0.395	0.436
FAL	0.495	0.356
EL	**0.576**	0.415
HL	**0.817**	-0.029
HG	**0.660**	-0.324
HC	**0.536**	**-0.536**
TL	0.148	0.007
SPW	0.381	**0.562**
HW	**0.670**	0.039
CD	**0.659**	0.307
WH	**0.869**	0.047
RH	**0.847**	0.158
BL	**0.753**	0.152
SIL	**0.826**	-0.114
Eigenvalue	3.986	1.435
Canonical correlation	0.896	0.771
P level of significance	0.001	0.001
Variance accounted for %	0.634	0.228

MC: Muzzle circumference, HW: Head width, FAL: Face length, EL: Ear length, HL: Horn length, HG: Heart Girth, HC: Hock circumference, TL: Tail length, SPW: Shoulder point width, HW: Hip Width, CD: Chest depth, WH: Withers height, RH: Rump height, BL: Body length, SIL: Scapula-ischial length
