# Supplementary material for: Confronting pastoralists’ knowledge of cattle breeds raised in the extensive production systems of Benin with multivariate analyses of morphological traits
Source: PLoS One. 2019 Sep 26;14(9):e0222756. doi: 10.1371/journal.pone.0222756 (PMC6762103; doi:10.1371/journal.pone.0222756)
Supplement: S5 Table — (PDF) [file pone.0222756.s006.pdf]

S5_Table. Pairwise Squared Mahalanobis distances among nine cattle breeds raised in Benin (based on data measured from both cows and bulls)

Cattle type	Bargouji	Boboji	Bodeeji	Dageeji	Goudali	Keteeji	Crossbreed	Somba	Yakanaji
Bargouji	0								
Boboji	9.33	0							
Bodeeji	20.02	27.32	0						
Dageeji	16.28	17.29	5.30	0					
Goudali	17.79	22.31	27.48	21.66	0				
Keteeji	6.29	19.63	14.53	16.28	17.30	0			
Crossbreed	9.61	10.86	7.35	3.93	11.98	9.31	0		
Somba	16.93	5.76	46.87	33.90	32.86	32.10	22.94	0	
Yakanaji	12.27	18.92	2.55	2.90	16.90	8.66	2.60	35.24	0
