# Supplementary figures and images for: Confronting pastoralists’ knowledge of cattle breeds raised in the extensive production systems of Benin with multivariate analyses of morphological traits
Source: PLoS One. 2019 Sep 26;14(9):e0222756. doi: 10.1371/journal.pone.0222756 (PMC6762103; doi:10.1371/journal.pone.0222756)

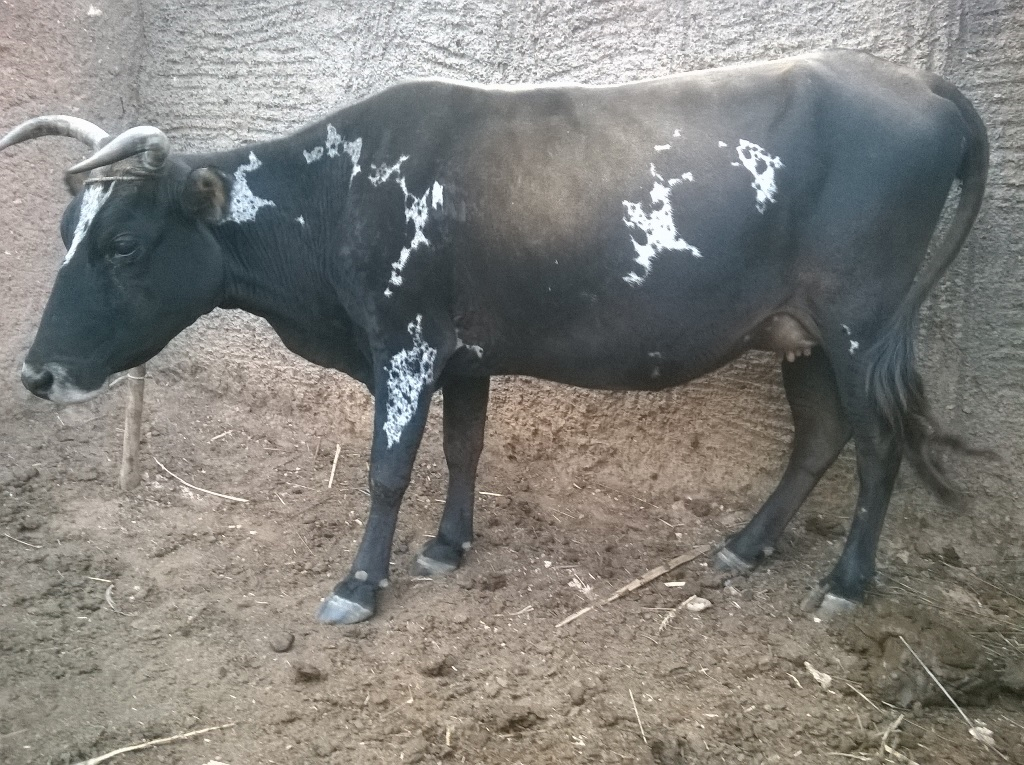

Supplement: S1 Fig — (TIF) [file pone.0222756.s007.tif]

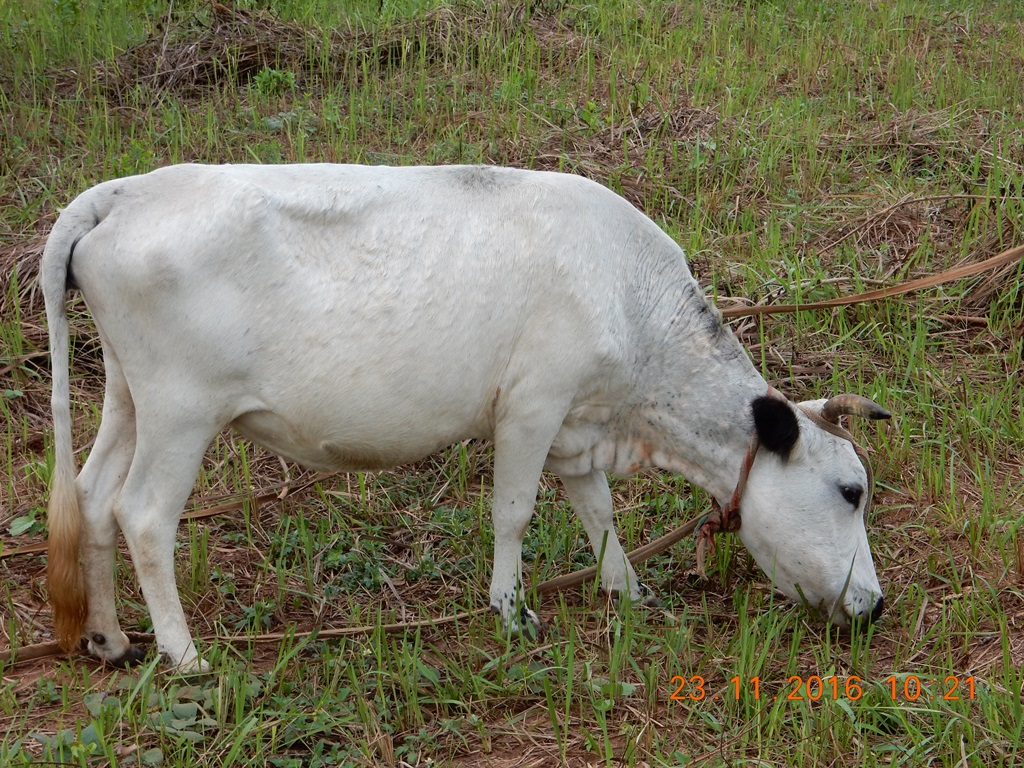

Supplement: S2 Fig — (TIF) [file pone.0222756.s008.tif]

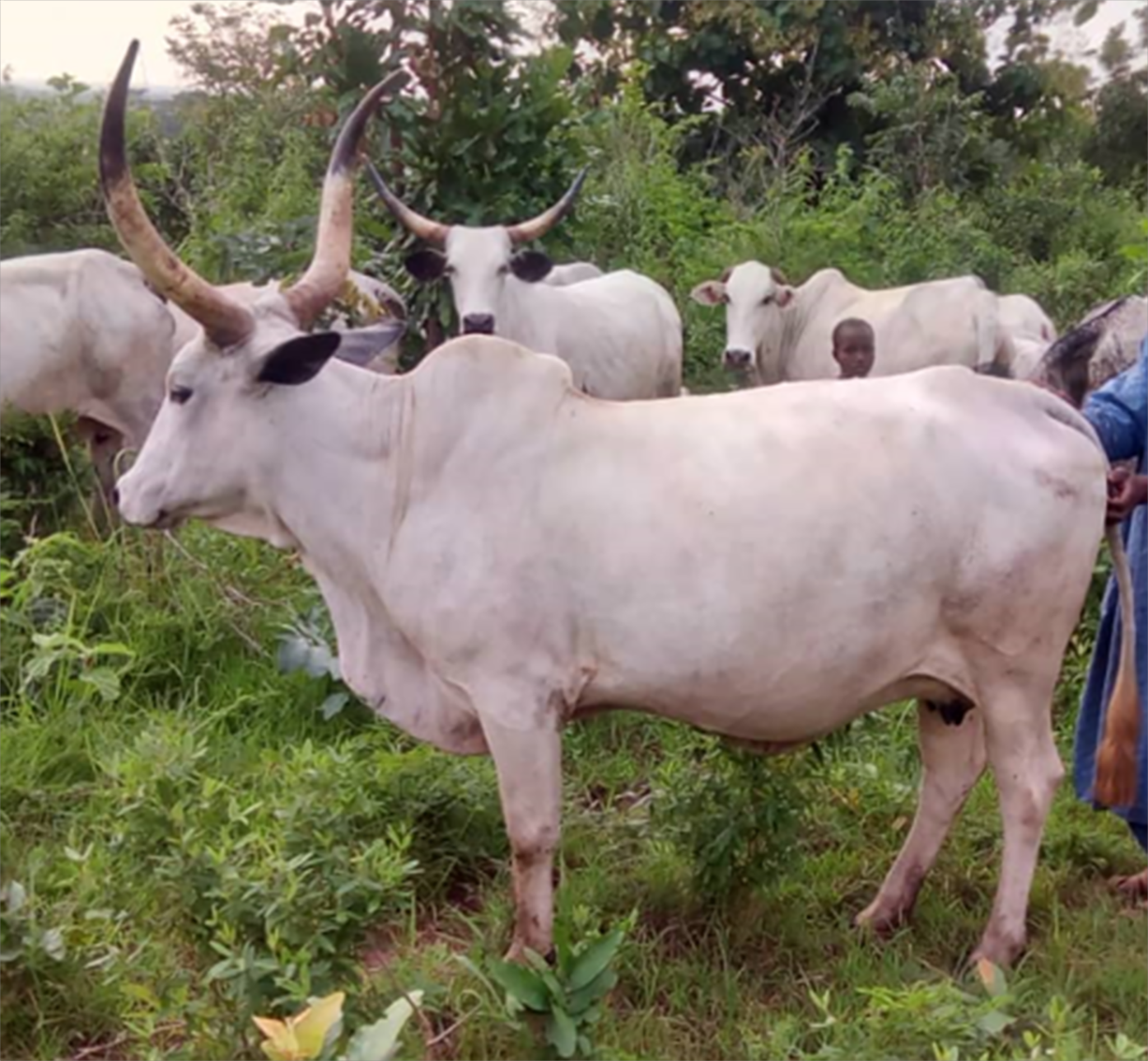

Supplement: S3 Fig — (TIF) [file pone.0222756.s009.tif]

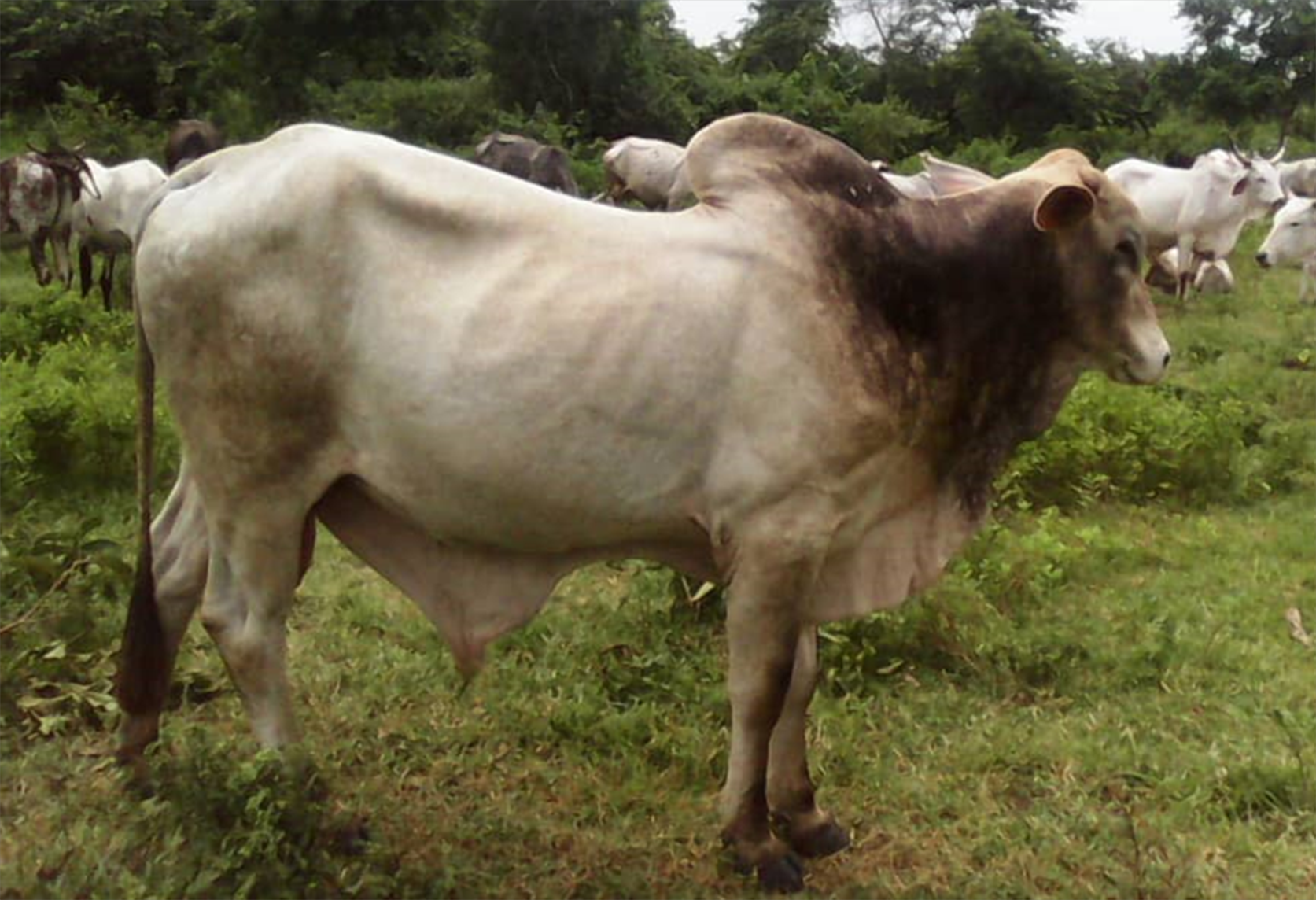

Supplement: S4 Fig — (TIF) [file pone.0222756.s010.tif]

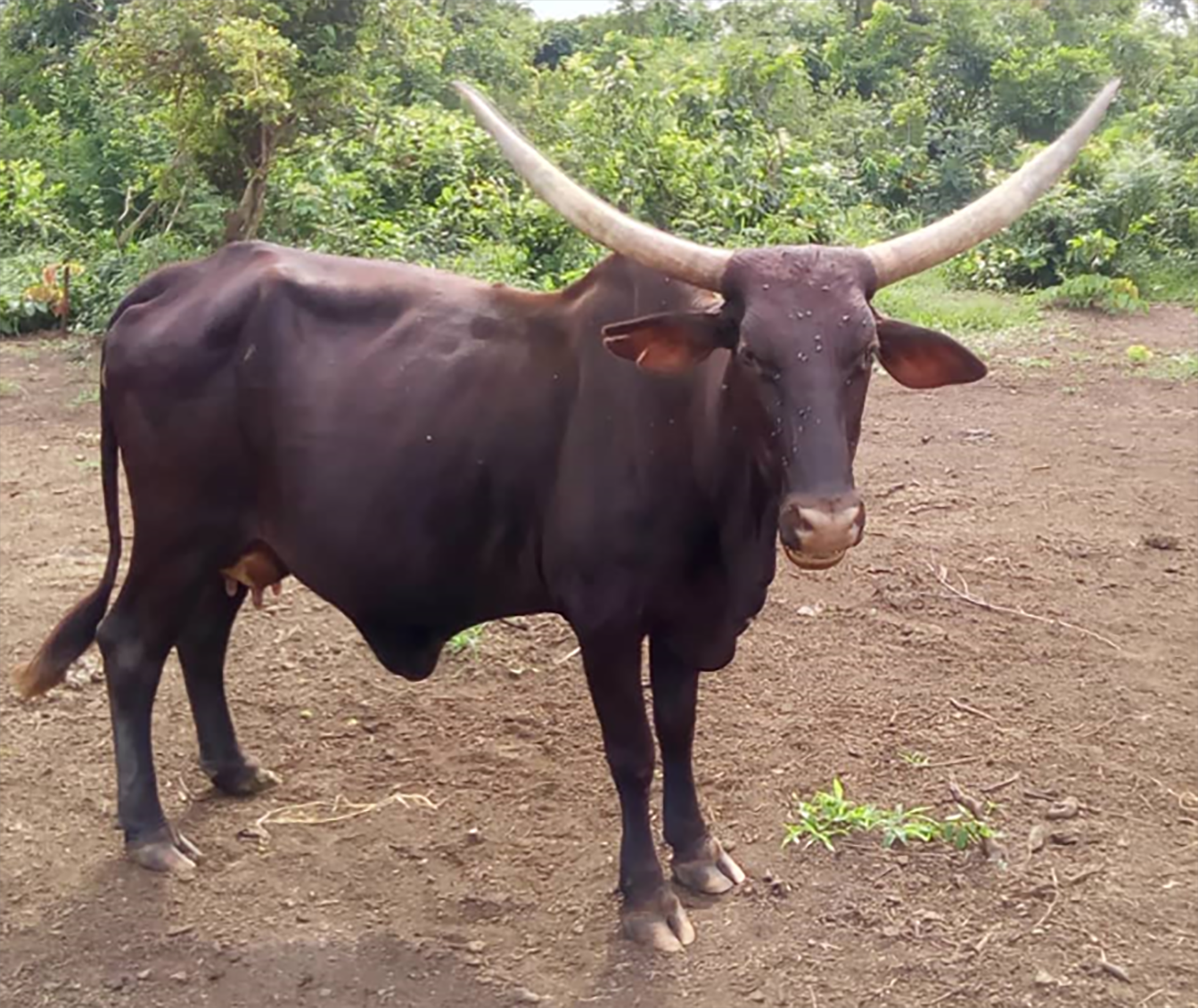

Supplement: S5 Fig — (TIF) [file pone.0222756.s011.tif]

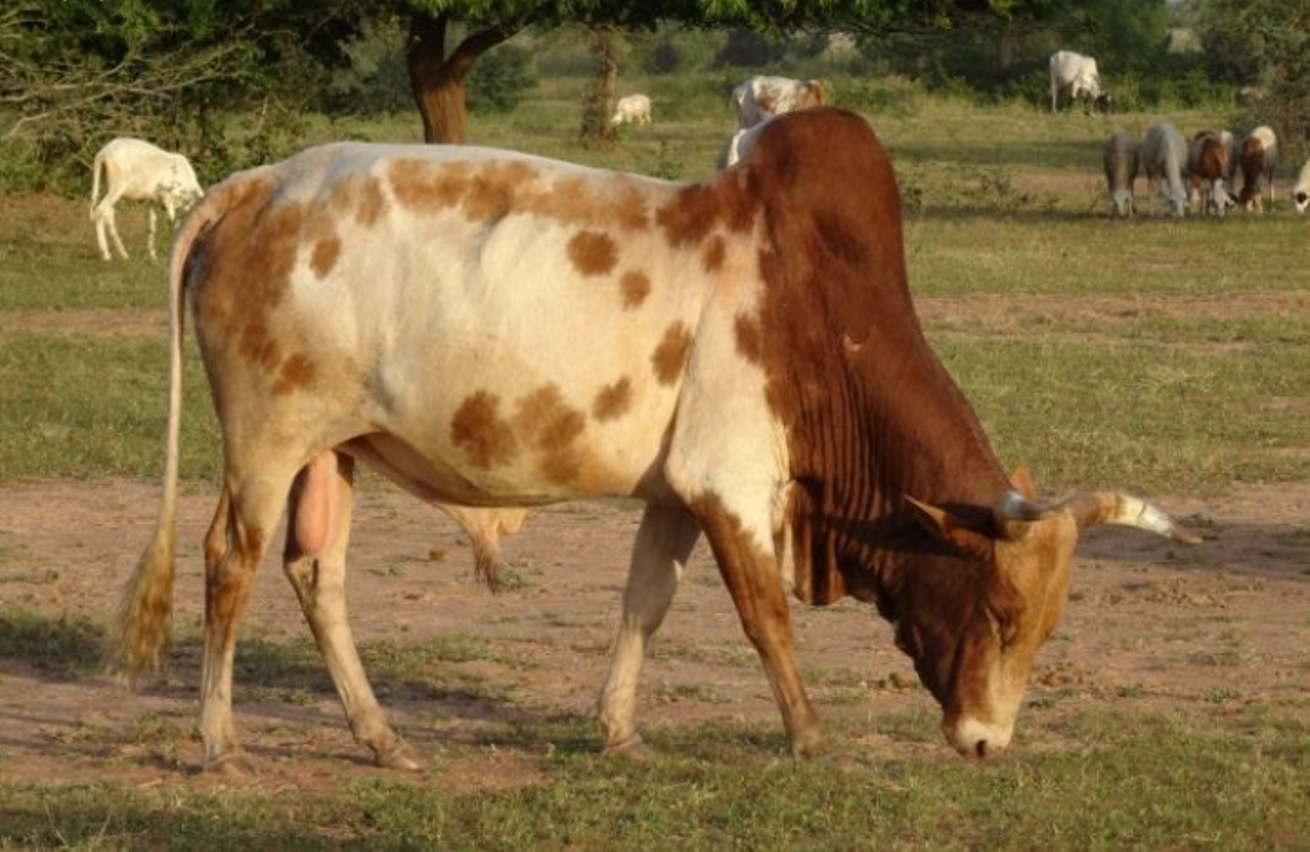

Supplement: S6 Fig — (TIF) [file pone.0222756.s012.tif]

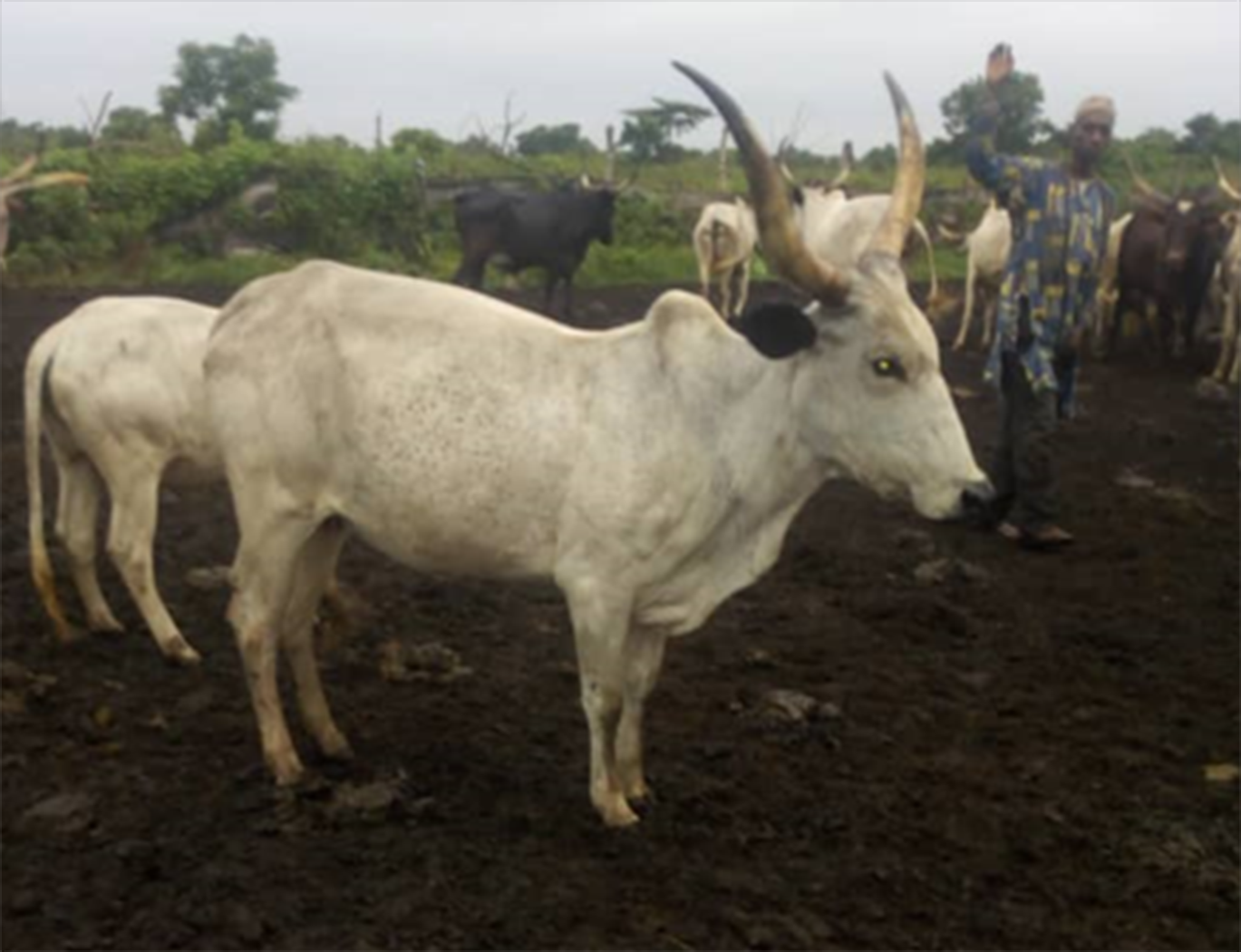

Supplement: S7 Fig — (TIF) [file pone.0222756.s013.tif]

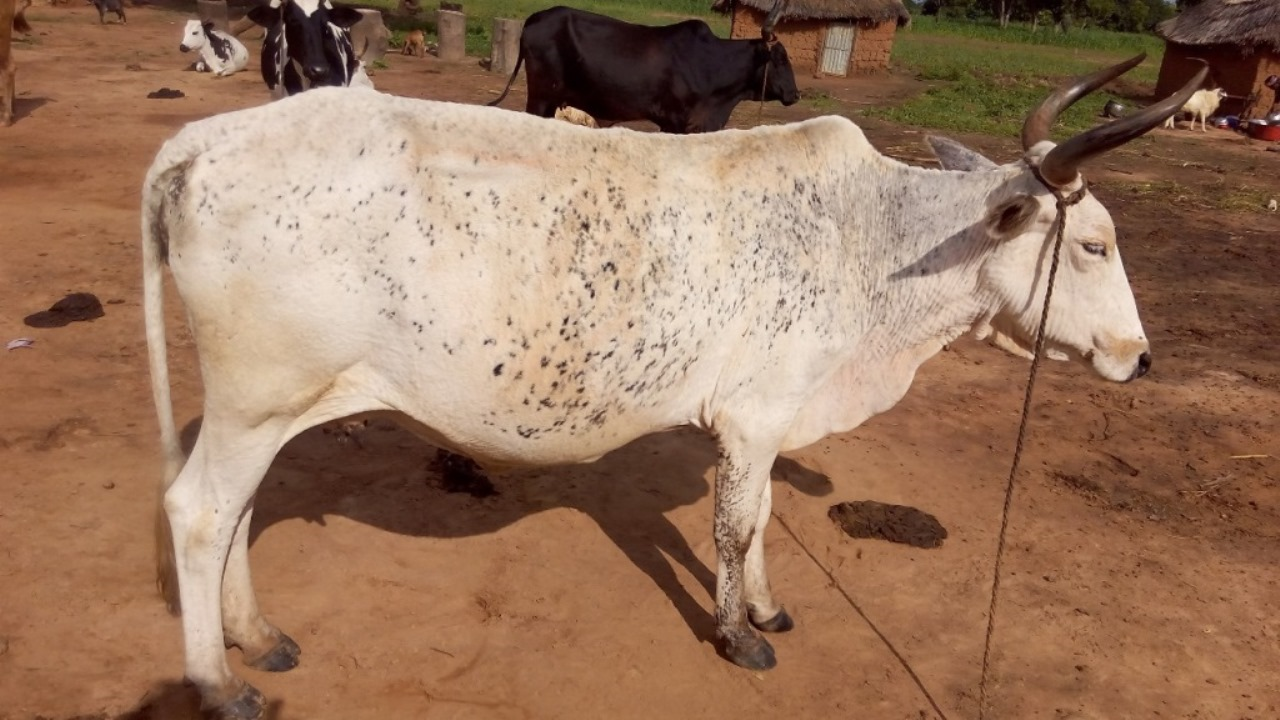

Supplement: S8 Fig — (TIF) [file pone.0222756.s014.tif]
