# Supplementary material for: Confronting pastoralists’ knowledge of cattle breeds raised in the extensive production systems of Benin with multivariate analyses of morphological traits
Source: PLoS One. 2019 Sep 26;14(9):e0222756. doi: 10.1371/journal.pone.0222756 (PMC6762103; doi:10.1371/journal.pone.0222756)
Supplement: Attachment — Submitted filename: PONE-D-19-14824_reviewer.pdf [file pone.0222756.s015.pdf]

PLOS ONE

Confronting pastoralists’ knowledge of cattle breeds raised in the extensive production systems of Benin with multivariate analyses of morphological traits

--Manuscript Draft--

Manuscript Number:	PONE-D-19-14824
Article Type:	Research Article
Full Title:	Confronting pastoralists’ knowledge of cattle breeds raised in the extensive production systems of Benin with multivariate analyses of morphological traits
Short Title:	Pastoralists’ knowledge and multivariate analyses for cattle breed characterization
Corresponding Author:	Luc Hippolyte Dossa<br>Universite d'Abomey-Calavi<br>Cotonou, BENIN
Keywords:	Livestock production systems; pastoral systems; cattle farming; cattle breeds; breed characterization; morphological traits; phenotype; multivariate analysis; farmers' traditional knowledge; farm animal genetic resources; local breeds
Abstract:	Cross-border seasonal livestock movements in West Africa bring into close contact several cattle breeds. In the coastal countries hosting migrating herders from the Sahel, it often affects the genetic variability and geographical distribution of traditional cattle breeds, through their indiscriminate but also intended crossbreeding with larger-framed Sahelian cattle breeds. The need to secure and effectively manage this genetic variability, in order to respond to changing production and market conditions, is widely recognized by the scientific community, livestock keepers and policy-makers. This however requires a comprehensive knowledge of the breeds’ characteristics- The indigenous criteria used by pastoralists to characterize and distinguish cattle breeds remain unclear and further validation is required. This study was therefore designed to document and validate herders' knowledge on cattle breeds. From June 2015 to June 2016, 803 cattle herders participated in a phenotypic breed description in seven pastoral communities across the country. Each cattle herder was asked to name and describe morphologically the different cattle breeds in his herd. Subsequently, fifteen body measurements taken on a total of 1401 adult cattle (964 cows and 439 bulls) were submitted to multivariate analyses. Participants distinguished ten different cattle breeds kept in traditional herds according to six primary morphological traits and clearly separated zebuine from taurine breeds. These results were consistent with those of the multivariate analyses of the measured traits. However, herders’ classification approach proved to be more accurate in distinguishing breeds within the zebuine subspecies. Hence, while metric measurements and molecular genetic analyses are promising approaches to fill the knowledge gap on the diversity of local farm animal genetic resources, they should integrate livestock keepers’ traditional knowledge for more precision.
Order of Authors:	Sandrine O. Houessou
	Luc Hippolyte Dossa
	Rodrigue Vivien Cao Diogo
	Maurice Cossi Ahozonlin
	Mahamadou Dahouda
	Eva Schlecht
Additional Information:	
Question	Response
**Financial Disclosure**<br><br>Enter a financial disclosure statement that describes the sources of funding for the	1) Initials of the authors who received each award: LHD<br>2) Grant number: Az 89 367<br>3) The full name of the funder: Volkswagen Foundation, Germany (DE)<br>4) URL: https://www.volkswagenstiftung.de/

Powered by Editorial Manager® and ProduXion Manager® from Aries Systems Corporation

work included in this submission. Review the submission guidelines for detailed requirements. View published research articles from *PLOS ONE* for specific examples.<br><br>This statement is required for submission and **will appear in the published article** if the submission is accepted. Please make sure it is accurate.<br><br>**Unfunded studies**<br>Enter: *The author(s) received no specific funding for this work.*<br><br>**Funded studies**<br>Enter a statement with the following details:<br>• Initials of the authors who received each award<br>• Grant numbers awarded to each author<br>• The full name of each funder<br>• URL of each funder website<br>• Did the sponsors or funders play any role in the study design, data collection and analysis, decision to publish, or preparation of the manuscript?<br>• **NO** - Include this sentence at the end of your statement: *The funders had no role in study design, data collection and analysis, decision to publish, or preparation of the manuscript.*<br>• **YES** - Specify the role(s) played.<br><br>* typeset	5) The funders had no role in study design, data collection and analysis, decision to publish, or preparation of the manuscript.
**Competing Interests**<br><br>Use the instructions below to enter a competing interest statement for this submission. On behalf of all authors, disclose any competing interests that could be perceived to bias this work—acknowledging all financial support and any other relevant financial or non-financial competing interests.<br><br>This statement **will appear in the published article** if the submission is accepted. Please make sure it is accurate. View published research articles from *PLOS ONE* for specific examples.	The authors have declared that no competing interests exist.

**NO authors have competing interests**<br><br>Enter: *The authors have declared that no competing interests exist.*<br><br>**Authors with competing interests**<br><br>Enter competing interest details beginning with this statement:<br><br>*I have read the journal's policy and the authors of this manuscript have the following competing interests: [insert competing interests here]*<br><br>* typeset	
**Ethics Statement**<br><br>Enter an ethics statement for this submission. This statement is required if the study involved:<br><br>• Human participants<br>• Human specimens or tissue<br>• Vertebrate animals or cephalopods<br>• Vertebrate embryos or tissues<br>• Field research<br><br>Write "N/A" if the submission does not require an ethics statement.<br><br>General guidance is provided below. Consult the submission guidelines for detailed instructions. **Make sure that all information entered here is included in the Methods section of the manuscript.**	N/A

**Format for specific study types**<br><br>**Human Subject Research (involving human participants and/or tissue)**<br>• Give the name of the institutional review board or ethics committee that approved the study<br>• Include the approval number and/or a statement indicating approval of this research<br>• Indicate the form of consent obtained (written/oral) or the reason that consent was not obtained (e.g. the data were analyzed anonymously)<br><br>**Animal Research (involving vertebrate animals, embryos or tissues)**<br>• Provide the name of the Institutional Animal Care and Use Committee (IACUC) or other relevant ethics board that reviewed the study protocol, and indicate whether they approved this research or granted a formal waiver of ethical approval<br>• Include an approval number if one was obtained<br>• If the study involved *non-human primates*, add *additional details* about animal welfare and steps taken to ameliorate suffering<br>• If anesthesia, euthanasia, or any kind of animal sacrifice is part of the study, include briefly which substances and/or methods were applied<br><br>**Field Research**<br><br>Include the following details if this study involves the collection of plant, animal, or other materials from a natural setting:<br>• Field permit number<br>• Name of the institution or relevant body that granted permission	
**Data Availability**<br><br>Authors are required to make all data underlying the findings described fully available, without restriction, and from the time of publication. PLOS allows rare exceptions to address legal and ethical concerns. See the PLOS Data Policy and FAQ for detailed information.	Yes - all data are fully available without restriction

A Data Availability Statement describing where the data can be found is required at submission. Your answers to this question constitute the Data Availability Statement and **will be published in the article**, if accepted.<br><br>**Important:** Stating 'data available on request from the author' is not sufficient. If your data are only available upon request, select 'No' for the first question and explain your exceptional situation in the text box.<br><br>Do the authors confirm that all data underlying the findings described in their manuscript are fully available without restriction?	
**Describe where the data may be found in full sentences. If you are copying our sample text, replace any instances of XXX with the appropriate details.**<br><br>• If the data are **held or will be held in a public repository**, include URLs, accession numbers or DOIs. If this information will only be available after acceptance, indicate this by ticking the box below. For example: *All XXX files are available from the XXX database (accession number(s) XXX, XXX.*).<br>• If the data are all contained **within the manuscript and/or Supporting Information files**, enter the following: *All relevant data are within the manuscript and its Supporting Information files.*<br>• If neither of these applies but you are able to provide **details of access elsewhere**, with or without limitations, please do so. For example:<br><br>*Data cannot be shared publicly because of [XXX]. Data are available from the XXX Institutional Data Access / Ethics Committee (contact via XXX) for researchers who meet the criteria for access to confidential data.*<br><br>*The data underlying the results presented in the study are available from (include the name of the third party*	All relevant data are within the manuscript and its Supporting Information files.

and contact information or URL).<br>• This text is appropriate if the data are owned by a third party and authors do not have permission to share the data.<br><br>* typeset	
Additional data availability information:	

Manuscript

Click here to access/download;Manuscript;MS pastoralists breed characterization.docx

Confronting pastoralists' knowledge of cattle breeds raised in the extensive production systems of Benin with multivariate analyses of morphological traits

Sandrine O. Houessou [1], Luc Hippolyte Dossa[1*], Rodrigue Vivien Cao Diogo[2], Maurice Cossi Ahozonlin[1], Mahamadou Dahouda[1], Eva Schlecht[3]

[1]Ecole des Sciences et Techniques de Production Animale, Faculté des Sciences Agronomiques, Université d'Abomey- Calavi, 03 BP 2819 Cotonou Jéricho, Bénin

[2]Département des Sciences et Techniques de Productions Animale et Halieutique, Université de Parakou, Faculté d'Agronomie, BP 123 Parakou, Bénin

[3]Animal Husbandry in the Tropics and Subtropics, University of Kassel and University of Göttingen, Steinstrasse 19, 37213 Witzenhausen, Germany

*Corresponding author: hippolyte.dossa@fsa.uac.bj; dolhip@yahoo.com

Abstract

Cross-border seasonal livestock movements in West Africa bring into close contact several cattle breeds. In the coastal countries hosting migrating herders from the Sahel, it often affects the genetic variability and geographical distribution of traditional cattle breeds, through their indiscriminate but also intended crossbreeding with larger-framed Sahelian cattle breeds. The need to secure and effectively manage this genetic variability, in order to respond to changing production and market conditions, is widely recognized by the scientific community, livestock keepers and policy-makers. This however requires a comprehensive knowledge of the breeds' characteristics- The indigenous criteria used by pastoralists to characterize and distinguish cattle breeds remain unclear and further validation is required. This study was therefore

designed to document and validate herders' knowledge on cattle breeds. From June 2015 to June 2016, 803 cattle herders participated in a phenotypic breed description in seven pastoral communities across the country. Each cattle herder was asked to name and describe morphologically the different cattle breeds in his herd. Subsequently, fifteen body measurements taken on a total of 1401 adult cattle (964 cows and 439 bulls) were submitted to multivariate analyses. Participants distinguished ten different cattle breeds kept in traditional herds according to six primary morphological traits and clearly separated zebuine from taurine breeds. These results were consistent with those of the multivariate analyses of the measured traits. However, herders' classification approach proved to be more accurate in distinguishing breeds within the zebuine subspecies. Hence, while metric measurements and molecular genetic analyses are promising approaches to fill the knowledge gap on the diversity of local farm animal genetic resources, they should integrate livestock keepers' traditional knowledge for more precision.

Keywords: animal genetic resource, bovine, breed characterization, integrative approach, local knowledge, morphology.

Introduction

West Africa is rich in a wide variety of cattle breeds represented by the taurine or *Bos taurus* and Zebu or *Bos indicus* subspecies [1,2]. Exposed to complex social, political and environmental processes over centuries, these genetic resources have developed under harsh conditions to which they have adapted [3]. They are mostly characterized by various genetic adaptive and performance traits, namely, their resistance to diseases and drought, their ability to walk long distances, their capacity to survive on poor pastures, and their high fertility [2].

Yet, this rich diversity is increasingly threatened by genetic dilution due to changes in production systems, livestock keepers' preferences for specific breeds and/or traits, market conditions and opportunities [4]. Several studies revealed admixtures among the taurine and zebu subspecies [5,6,7] as the result of the continuous genetic flow that occurs every year during seasonal cross-border livestock movements from the drier Sahelian zones in the north to the more humid zones in the south of West and East Africa. The importance of these local genetic resources for the livelihoods of livestock keepers and the sustainability of the production systems in which they are raised calls for the urgent need to promote their sustainable use [8,9,10,2] and conservation [11]. The latter author has argued that biodiversity conservation and food security are two sides of the same coin.

Yet, as revealed by the review of existing literature, the first obstacle to sustainable management of local farm animal genetic resource in West African livestock production systems is the insufficient knowledge on their specific features and genetic diversity. In Benin, like in several African countries, there are virtually no inventories of these resources and thus no reliable data available. The precise identification of animal types and breeds, and an improved understanding of their values or adaptive traits are thus necessary but depend on the availability of accurate and comprehensive information on their characteristics as well as their production and marketing environments.

The Second Report on the State of the World's Animal Genetic Resources for Food and Agriculture [12] acknowledged recent and ongoing efforts of description and characterization of livestock breeds in several West African countries. But most of these efforts tend to be fragmentary and limited to their phenotypic [13,14,15,16], genotypic and molecular characterization [7,17,18,19,20,21] out of the production system context, paying little attention to the local knowledge of the communities who keep them.

Indeed, the characterization of livestock breeds can be performed through well-designed phenotypic, genetic and molecular studies that include pertinent and well thought-out analysis and interpretation of quantitative data. Quantitative morphological characterization represents the first step in the characterization process [22,23,24]. The comparison of morphological characteristics can provide, to some extent, a reasonable representation of genetic difference among populations [25]. Multivariate discriminant analyses have been reported, in several previous studies, to be effective for a precise and objective discrimination of different populations of cattle [13,14,26,15]; goats [25,27], sheep [28,29,30], and horses [31]. But according to [32], mere biological breed characterization may not be adequate. These authors argued that livestock keepers' indigenous knowledge should be an integral part of breed characterization.

Pastoralism, the use of extensive grazing on rangelands for livestock production, which includes nomadism, transhumance and agro-pastoralism [33], is still the dominant ruminant livestock system in Sub-Saharan Africa [34]. Cattle are the basic assets of the pastoralists, and cattle breeds are the subject of much discussion among herders [35] who have accumulated a wealth of untapped knowledge of these farm animal genetic resources and of their production environments [36,37,38,39]. The need to secure and effectively manage the farm animal genetic resources in order to respond to changing production and market conditions is widely recognized by the scientific community, farmers and policy-makers [8,40,41]. Hence, the genetic variability within and among these farm animal genetic resources, together with the related traditional knowledge need to be documented and valorized for their effective management through policy interventions [32,42]. [42] further argues that in traditional livestock production systems, where breed association records are absent and/or molecular studies are not affordable, this traditional knowledge likely provides the best preliminary

information available about breed identity that can serve as a valuable starting point for understanding variation in key phenotypic traits.

There have been recent quantitative morphological characterizations of cattle breeds in West Africa [16,43,44,45] and in Benin [46]. Also, very few studies [47,48] have investigated West African pastoralists' local knowledge of breeds and breeding practices. So far, however, no single study exists which combines the two approaches with the aim to test their complementarity and validate pastoralists' classification of cattle breeds. The aim of this study was to test the hypothesis that pastoralists' classification system is more accurate in differentiating cattle breeds than quantitative morphological analyses.

Material and methods

Study sites

The study was conducted in seven (07) localities along the geographical north-south gradient in Benin (Fig. 1). These locations, whose climatic and demographic characteristics are presented in Table 1, were selected because of the presence of relatively high numbers of resident cattle herds but also because of the important influx of seasonal and cyclical migrating herds in search of pasture and water. These migrating herds are further designed as transhumant herds.

Fig. 1 ***Map of Benin showing the locations of the municipalities investigated.*** [*Nearly here*]

Table 1. General characteristics of the study locations and number of cattle herds surveyed in Benin

Location	Geographic coordinates	Region	Agro-ecological zone [(a)]	Annual rainfall (mm) [(a)]	Rainy season [(a)]	Climate [(a)]	Soil type [(a)]	Area (km²)	Population density (people/km²)[(b)]	Estimated total cattle population (heads) [(c)]	Cattle herds sampled (n)
Kétou	7° 21′ N 2° 36′ E	South	GCZ	900-1300	Bimodal	Sub-equatorial	Ferralitic	1775	88	16000	120
Agonli	7° 13′ N 2° 20′ E	South	GCZ		Bimodal			1758	132	6170	50
Tchaourou	8° 53′ N 2° 36′ E	Centre	GSZ	1100-1200	Unimodal	Tropical subhumid	Ferruginous	7256	30	47000	110
Savalou	7° 55′ N 1° 58′ E	Centre	GSZ	1100	Bimodal			2674	54	32000	120
Kandi	11° 07’N 2° 56’ E	North-East	SZ	900-1000	Unimodal	Dry tropical	Ferruginous	3421	52	159000	148
Sinendé	10° 20′ N 2° 22′ E	North-East	SZ	1000-1050	Unimodal			2289	39	80000	135
Boukombé	10° 11′ N 1° 06′ E	North-West	SZ	1000-1200	Unimodal			1 036	80	30100	120

[(a)]Akoègninou et al. (2006) : GCZ=Guinea-Congolian Zone, GSZ=Guinea Sudanian Zone, SZ=Sudanian Zone; [(b)]INSAE (2015) ; [(c)] FAOSTAT (2017)

Data collection

Ethical statement

The study involved taking body measurements from cattle with the consent and in the presence of the cattle farmer. There is no specific legislation for body measurements and hence no approval was necessary. All the data was collected in traditional farms and the animal owners agreed to be involved in the project through the Regional Action Centre for Rural Development (CARDER) which is the decentralized institution for the management of the agricultural sector in the surveyed municipalities. Furthermore, the study did not involve any aversive animal handling, any anesthesia, euthanasia, or kind of animal sacrifice. All the animals included in this study were managed in accordance with the criteria for the assessment of animal welfare identified by the Welfare Quality Project (WQP) reported by [73].

Assessment of pastoralists' knowledge about cattle breeds' characteristics

First, informal interviews and group discussions were organized in each of the research locations with officers of local extension services responsible for livestock production, and with other key resource persons to: i) obtain more data and information about cattle production and preliminary information about the different cattle breeds kept in the area; ii) identify villages for the breed survey, and iii) obtain lists of cattle camps and the places where cattle herds and herders gather.

Subsequently, these camps and places were visited and meetings were held with cattle herders, to explain the objectives of the study, obtain their informed consent to participate in the survey, and arrange with them the adequate time for conducting interviews. Herders' participation in the survey was on a voluntary basis. Individual interviews were then conducted with a total of 803 cattle herders distributed across the seven locations (Table 1). The herders were asked to name in their local language the breeds of cattle kept in their area and to indicate the most

relevant traits they used to distinguish between them. When and where necessary, the interviews were carried out with the help of translators.

Morphological characterization

In each surveyed herd, one mature cow and, where available, one breeding bull representative of each cattle breed present and named by the herder were then randomly selected for morphometric measurements. They maturity was ascertained by the visual examination of their dentition (possession of either three or four pairs of permanent teeth). A total of 1401 adult cattle of both sexes (943 cows and 458 bulls) was measured and included in this study.

Fifteen body measurements and ten qualitative traits (Table 2) were assessed following the FAO guidelines for phenotypic characterization of animal genetic resources [24]. The qualitative traits described were sex, presence of horn, horn color, horn shape, cephalic profile, ear shape, ear orientation, body coat color and body coat color pattern, presence of hump and dewlap size. The body measurements were carried out using a measuring stick, a measuring tape or wooden caliper on animals standing on a level surface and maintained in upright posture by their respective owners.

Table 2. Morphological traits measured on 1401 individual cattle across seven locations in Benin

Variable	Description
Quantitative (in cm)	
Height at withers (WH)	Vertical distance from the bottom of the front foot to the highest point of the
Rump height (RH)	Distance from the highest point of rump to the ground
Heart girth (HG)	Circumference of body just behind the forelegs
Body length (BL)	Distance between the horn site to tail drop
Scapula-ischial length (SIL)	Distance from tip of the shoulder to the ischial tuberosity
Face length(FAL)	Distance from between the horn site to the lower lip
Ear length (EL)	Distance from the point of attachment to the tip of the ear
Head width (HW)	Distance between the most prominent points of the zygomatic arches
Tail length (TL)	Distance from the tail drop to the tip of the tail
Hip Width (HW)	Distance between the ends of the bone of the iliac crest
Horn length (HL)	Distance from the root of the horn to its tip along the outer curvature
Hock circumference (HC)	Circumference taken just above the hock joint
Muzzle circumference (MC)	Complete distance around the outside of the mouth
Chest depth (CD)	Vertical distance from the apex of the withers to the bottom of the chest
Shoulder point width (SPW)	Distance between the right and left shoulder points
Qualitative	
Sex	Male, female
General aspect of the coat	Uniform, Spotted, Composed
Unique color of coat	Black, White, Dark red, Brown, Fawn
Other color of coat	White spotted black, Black spotted white, White spotted red, Red spotted white
Cephalic profile	Concave, Convex, Straight
Presence of hump	Absent, Present
Presence of horn	Absent, Present
Color of horn	Black, Brown, White, Black and Brown, Black and White, Brown and white
Horn shape	Straight, Crown, Cup, Folded back cup, Crescent, Lyre, Folded back lyre, Wheel, Spiral, Numeral three
Ear shape	Rounded, Pointed
Orientation of ear	Erected, Horizontal, Dropping

Statistical analysis

Collected data were processed and analyzed using SAS Software Version 9.2 [49]. Metric data were first checked for consistency. This resulted in the exclusion of the five animals named by herders as of *Djelliji* breed and one male of *Dageeji* breed.

Descriptive analyses were firstly performed to explore statistical differences among the different cattle breeds. Frequencies and chi-square ($\chi 2$) tests for independence were performed on the aforementioned qualitative traits to explore statistical differences among breeds. Similarly, the quantitative variables were subjected to analysis of variance using PROC GLM. The least square means (LSMEANS) were calculated for males and females separately, and for both together. Comparison of means between groups was performed using the Students Newman and Keuls (SNK) multiple mean comparison tests. The results were used to screen for the most useful variables for further discriminant analysis. The canonical discriminant analysis using the CANDISC procedure was performed to determine the best linear combination of the quantitative variables that would group or separate the named cattle breeds. Canonical variables that summarize between-breeds variation were generated and the pairwise squared Mahalanobis distances calculated. PROC GPLOT was used to plot the individuals onto canonical variables for visual examination of the ordering of the different breeds in the multivariate space. The ability of these canonical functions to assign each individual animal to its original group was calculated as the percentage of correct assignment to each genetic group using the DISCRIM procedure (Nearest Neighbour Discriminant Analysis). The degree of morphological similarity or divergence between the investigated cattle breeds was assessed and their classification in homogenous groups was carried out using the method of Ascending Hierarchical Clustering (AHC) according to the criterion of the jump of Ward in the PROC CLUSTER and TREE procedures. The Mahalanobis distances generated during the canonical discriminant analysis were used to construct a dendrogram using the Unweighted Pair Group Method Analysis

(UPGMA). Finally, the association between the qualitative traits was investigated through a Multiple Correspondence Analysis (MCA) using PROC CORRESP.

Results

Herders' perceptions of morphological characteristics of the main cattle breeds

A total of ten cattle breeds were identified from the surveyed herders' responses and named as follows: *Boboji*, Somba, *Yakanaji*, Gudali or *Bokoloji*, *Bodeeji* or Bororo, *Djelliji*, *Dageeji/Dage*, *Bargouji/Muti*, *Keteeji* and one unnamed type perceived as crossbreed

The following morphological traits were used by herders to distinguish among different cattle breeds: body size (dwarf, small, middle, large), size of horn (absent, short, middle, long, long and white), hump size (absent, small, poorly developed, well developed), size of sheath/ umbilical fold (small, poorly developed, well developed), dewlap size (small, poorly developed, well developed), coat color (white, reddish-brown/ black, white/reddish, variable, white and black neck).

Table 3 presents the characteristics of the different cattle breeds according to the participating herders. The two taurine breeds, namely Somba and *Boboji*, were described with similar characteristics (variable coat color, very small size, short horns and absence of hump).

The five zebu breeds (*Yakanaji*, *Gudali*, *Bodeeji*, *Djelliji*, and *Dageeji)* also shared many similarities. With the exception of the hornless *Gudali* and *Djelliji* whose horn was perceived by respondents as of medium size, the zebus were, in their majority, described as large-framed and long-horned animals with a well or poorly developed hump. The Bodeeji breed was further

differentiated from the Yakanaji breed by its reddish brown to red dark coat color and white horns.

Table 3. Key morphological traits used by farmers (%) to classify nine cattle breeds raised in Benin

Trait	Trait expression	Cattle breeds							
		Yakanaji	*Goudali*	*Bodeeji*	*Djelliji*	*Dageeji*	*Bargouji*	*Boboji*	Somba
Coat color		(n=407)	(n=463)	(n=422)	(n=84)	(n=261)	(n=298)	(n=111)	(n=100)
	White	100.0	84.9	0.0	0.0	100.0	95.6	0.0	0.0
	Reddish Brown / Black	0.0	0.0	100.0	0.0	0.0	0.0	0.0	0.0
	White/Reddish	0.0	0.0	0.0	98.8	0.0	0.0	0.0	0.0
	Variable	0.0	0.0	0.0	1.2	0.0	4.4	100.0	100.0
	White and black neck	0.0	15.1	0.0	0.0	0.0	0.0	0.0	0.0
Body size		(n=477)	(n=396)	(n=376)	(n=215)	(n=217)	(n=400)	(n=215)	(n=220)
	Large	95.6	100.0	99.7	96.7	0.0	0.0	0.0	0.0
	Medium	4.4	0.0	0.0	3.3	100.0	0.0	0.0	0.0
	Small	0.0	0.0	0.3	0.0	0.0	100.0	0.0	0.0
	Dwarf	0.0	0.0	0.0	0.0	0.0	0.0	100.0	100.0
Size of horn		(n=527)	(n=557)	(n=418)	(n=209)	(n=251)	(n=380)	(n=170)	(n=219)
	Absent	0.0	100.0	0.0	0.0	0.0	0.0	0.0	0.0
	Long	100.0	0.0	100.0	0.0	100.0	0.0	0.0	0.0
	Medium	0.0	0.0	0.0	100.0	0.0	0.0	0.0	0.0
	Short	0.0	0.0	0.0	0.0	0.0	100.0	100.0	100.0
Size of hump		(n=337)	(n=374)	(n=242)	(n=202)	(n=116)	(n=418)	(n=138)	(n=210)
	Absent	0.0	0.0	0.0	0.0	0.0	35.6	100.0	100.0
	Well developed	100.0	100.0	62.8	100.0	19.0	0.0	0.0	0.0
	Poorly developed	0.0	0.0	37.2	0.0	81.0	0.0	0.0	0.0
	Small	0.0	0.0	0.0	0.0	0.0	64.4	0.0	0.0
Size of dewlap		(n=174)	(n=223)	(n=163)			(n=120)		
	Well developed	0.0	100.0	0.0	-	-	0.0	-	-
	Poorly developed	100.0	0.0	100.0	-	-	0.0	-	-
	Small	0.0	0.0	0.0	-	-	100.0	-	-
Size of sheath/ umbilical folds		(n=202)	(n=247)	(n=54)		(n=44)	(n=82)		
	Well developed	100.0	100.0	0.0	-	0.0	0.0	-	-
	Poorly developed	0.0	0.0	100.0	-	36.4	0.0	-	-
	Small	0.0	0.0	0.0	-	63.6	100.0	-	-

Moreover, the *Dageeji* breed, in contrast to others, was perceived as a medium-sized zebu with a poorly developed hump and a small sheath/ umbilical fold.

The *Bargouji* breed was described as a shorthorn taurine-like cattle, but with a larger body size than taurine, a white coat, absence of hump or presence of small hump, a small dewlap and a small sheath/ umbilical fold. The morphological characteristics of *Keteeji* cattle were not presented separately because herders perceived *Bargouji/Muti* and *Keteeji* as two morphologically close cattle breeds. They however assumed that the pure *Bargouji* is generally humpless or has only a small hump whereas *Keteeji* is always humped with a small or poorly developed hump. They also asserted that *Keteeji* is sometimes larger in size than *Bargouji*.

Comparison of measured morphometric traits among breeds

The differences between the cattle breeds for the morphometric variables of male and female animals and their pooled data are presented in Table 4, Table 5 and S1_Table, respectively. All linear body measurements significantly varied ($P<0.05$) among breeds. Major morphological traits such as height at withers (WH), rump height (RH), body length (BL) and scapulo-ischial length (SIL) were significantly greater ($P<0.05$) for humped cattle breeds compared with humpless ones. Within breeds, they were also significantly greater ($P<0.05$) in bulls than in cows. The highest mean values of WH were recorded in *Bodeeji* cows followed by the *Yakanaji* cows whereas the lowest values were obtained in the *Boboji* and Somba cows. In terms of morphometric traits, these two taurine breeds presented no significant differences.

Table 4**.** Least square means (± standard error) and pairwise comparison of morphological traits measured in cows across nine cattle breeds raised in Benin

**Trait**	Cattle breeds								
	Bargouji (n=206)	*Boboji* (n=58)	*Bodeeji* (n=26)	*Dageeji* (n=23)	*Goudali* (n=22)	*Keteeji* (n=138)	Crossbreed (n=94)	Somba (n=84)	*Yakanaji* (n=312)
MC	40.6^{c} ± 0.37	43.1^{b} ± 0.60	46.5^{a}± 0.52	44.0^{b}± 0.44	40.3^{c}± 1.07	46.8^{a}± 0.32	43.6^{b}±0.36	40.1^{c}± 0.37	44.5^{b}±0.21
HW	19.5^{b} ± 0.15	19.2^{b} ± 0.23	20.1^{b}±0.18	19.2^{b}± 0.28	20.4^{b}± 0.57	23.5^{a}±0.33	20.3^{b}±0.24	17.4^{c}± 0.16	20.4^{b}±0.15
FAL	45.7^{c} ± 0.42	43.9^{d} ± 0.56	48.4^{b} ±0.21	47.6^{bc}± 0.67	46.0^{c}±0.60	46.9^{a}± 0.47	46.9^{bc}±0.36	39.9^{e}± 0.41	46.8^{bc}±0.3
EL	19.5^{cd} ± 0.22	17.4^{e} ± 0.22	20.6^{c} ± 0.26	19.0^{d}± 0.23	21.9^{b}± 0.63	23.4^{a} ±0.48	20.5^{c}±0.27	15.4^{f}± 0.13	20.7^{c}±0.18
HL	37.0^{e} ± 0.51	28.1^{f} ± 1.46	65.8^{a} ±1.86	49.1^{bc}± 1.74	14.1^{g}± 4.10	47.7^{c} ±1.04	42.4^{d}±1.51	15.8^{g}± 1.46	52.8^{b}±0.68
HG	152.8^{d} ±0.99	148.9^{d} ± 1.49	179.4^{a}±1.42	169.1^{b} ±2.12	171.8^{b}±2.60	159.1^{c}±0.91	166.4^{b}±1.15	142.5^{e}± 1.42	171.4^{b}±0.93
HC	35.6^{d} ±0.25	39.6^{c} ± 0.44	44.3^{b} ±0.60	47.4^{a} ±0.93	40.4^{c}±0.86	38.5^{c}±0.26	44.5^{b}±0.45	35.4^{d}± 0.54	43.5^{b}±0.62
TL	99.0^{ab} ± 0.83	87.6^{b} ± 1.01	103.6^{ab}± 1.06	97.8^{ab}± 0.69	103.9^{ab}±2.50	102.7^{ab}±1.35	109.7^{a}±9.65	91.7^{ab}± 0.74	103.1^{ab}±0.65
SPW	34.7^{b} ± 0.27	28.2^{d} ±0.44	33.3^{a}± 0.76	30.4^{c}± 0.42	33.9 ±1.03	36.4^{a}±0.44	32.3^{b}±0.42	29.0^{cd} ±0.43	36.6^{b}±0.38
HW	42.7^{b} ± 0.23	41.0^{c} ±0.51	45.8^{a}± 0.63	44.3^{ab}±0.37	46.0^{a}±1.01	45.1^{a}±0.31	44.3^{ab}±0.35	36.4^{d} ±0.37	46.4^{a}±0.25
CD	63.9^{a} ±0.35	54.6^{c}± 0.72	66.1^{a}±0.68	64.6^{a}±0.90	64.9^{a}± 1.46	67.0^{a}±0.51	60.8^{b}±0.46	49.8^{d}± 0.37	65.7^{a}±0.51
WH	115.9^{d} ± 0.63	110.5^{f}±0.83	137.8^{a}± 0.85	129.1^{b}± 1.32	130.3^{b} ±1.30	131.1^{b}±0.53	123.4^{c}±0.78	100.4^{f}±0.47	131.4^{b}±0.76
RH	117.4^{d} ±0.74	113.1^{e}±0.85	136.4^{a}±0.68	129.0^{bc}±1.02	129.8^{bc}±1.05	134.1^{a}±0.61	125.4^{c}±0.74	105.3^{f}±0.51	132.4^{b}±0.65
BL	115.9^{d} ± 0.63	114.6^{d}±1.07	132.6^{a}±0.94	124.4^{bc}± 1.18	120.8^{c}± 2.44	129.6^{a}±0.85	121.7^{c}±0.74	100.2^{e}±0.76	128.2^{b}±0.66
SIL	164.2^{d} ±0.75	150.4^{e}±1.74	193.2^{a}± 2.69	180.3^{bc}±1.49	173.9^{c}± 1.97	175.1^{c}±1.25	177.0^{c}± 2.22	141.1^{f}±0.78	184.4^{b}±1.39

^{a,b,c,d} Means with different superscript in the same row are significantly different ($P \leq 0.001$), SNK's multiple mean comparison test

MC: Muzzle circumference, HW: Head width, FAL: Face length, EL: Ear length, HL: Horn length, HG: Heart Girth, HC: Hock circumference, TL: Tail length, SPW: Shoulder point width, HW: Hip Width, CD: Chest depth, WH: Withers height, RH: Rump height, BL: Body length, SIL: Scapula-ischial length.

Table 5. Least square means in cm (± standard error) and pairwise comparison of morphological traits measured in bulls of nine cattle breeds raised in Benin

**Traits**	Cattle breeds							
	Bargouji (n=131)	*Boboji* (n=5)	*Bodeeji* (n=10)	*Goudali* (n=10)	*Keteeji* (n=93)	Crossbreed (n=16)	Somba (n=109)	*Yakanaji* (n=63)
MC	43,5^{b}±0,31	44,8^{abc}±1,96	44,2^{abc}±0,94	45,6^{abc}±1,09	46,2^{a}±0,39	47,5^{a}±1,19	42,2^{b}±0,34	48,0^{a}±0,52
HW	20,1^{ab}±0,17	19,8^{ab}±0,20	21,6^{ab}±0,58	22,0^{ab}±1,01	21,8^{ab}±0,27	23,1^{a}±1,02	18,0^{b}±0,17	22,5^{a}±0,38
FAL	49,2^{a}±0,28	45,8^{b}±1,74	48,9^{a}±0,97	48,2^{a}±0,79	51,2^{a}±0,30	50,0^{a}±1,18	40,1^{b}±0,38	50,4^{a}±0,56
EL	19,8^{a}±0,21	17,4^{b}±0,60	21,0^{c}±0,54	21,9^{c}±0,62	21,9^{c}±0,27	20,6^{c}±0,52	15,9^{b}±0,18	21,7^{c}±0,29
HL	34,6^{b}±0,82	28,2^{bc}±3,77	52,8^{a}±3,03	16,3^{c}±5,07	42,3^{ab}±1,06	40,8^{ab}±4,65	16,8^{c}±0,52	53,0^{a}±1,79
HG	155,4^{cd}±0,79	155,9^{cd}±5,04	160,2^{bc}±1,33	180,1^{a}±3,03	162,0^{bc}±0,97	174,9^{ab}±3,84	144,2^{d}±0,74	174,7^{ab}±1,99
HC	34,6^{b}±0,31	38,7^{b}±0,86	46,5^{a}±1,23	47,9^{a}±1,20	38,2^{b}±0,28	48,9^{a}±0,90	37,9^{b}±0,31	46,4^{a}±0,70
TL	101,8^{ab}±1,25	81,7^{c}±2,53	106,8^{ab}±2,14	118,4^{a}±3,56	107,7^{ab}±1,57	110,4^{ab}±2,49	93,4^{bc}±0,85	109,9^{ab}±1,52
SPW	34,8^{ab}±0,33	29,2^{b}±0,80	33,6^{ab}±0,88	35,4^{ab}±1,87	36,4^{a}±0,43	35,1^{ab}±0,98	29,0^{b}±0,35	36,6^{a}±0,63
HW	42,5^{a}±0,35	41,0^{ab}±2,07	42,9^{a}±1,09	44,9^{a}±1,56	43,7^{a}±0,41	45,5^{a}±0,91	36,4^{b}±0,40	47,4^{a}±0,55
CD	62,9^{b}±0,40	56,6^{b}±1,40	59,0^{b}±0,61	65,4^{ab}±1,22	64,7^{ab}±0,48	63,6^{ab}±1,40	51,8^{b}±0,48	68,5^{a}±0,90
WH	117,0^{b}±0,58	109,2^{bc}±1,22	137,0^{a}±1,41	135,1^{a}±1,41	128,8^{ab}±0,52	130,4^{a}±2,09	101,8^{c}±0,51	136,5^{a}±0,94
RH	123,6^{bc}±0,49	111,6^{c}±2,87	139,7^{a}±1,89	139,0^{a}±1,58	133,8^{a}±0,59	134,3^{a}±2,00	107,0^{c}±0,57	138,2^{a}±0,64
BL	119,4^{b}±0,71	115,8^{bc}±5,26	134,1^{a}±2,26	132,1^{a}±4,53	123,2^{b}±0,94	124,4^{b}±1,92	103,2^{c}±0,67	131,8^{a}±1,02
SIL	163.8^{b}±1.12	151.4^{c}±1.70	198.4^{a}± 0.90	182.8^{b}± 1.46	173.6^{ab}±3.15	184.8^{a}± 3.10	144.7^{c}±1.70	186.8^{a}±1.39

^{abc} Means with different letters in rows are significantly different between locations at P ≤ 0.001; SNK's multiple mean comparison test;

MC: Muzzle circumference, HW: Head width, FAL: Face length, EL: Ear length, HL: Horn length, HG: Heart Girth, HC: Hock circumference, TL: Tail length, SPW: Shoulder point width, HW: Hip Width, CD: Chest depth, WH: Withers height, RH: Rump height, BL: Body length, SIL: Scapula-ischial length

Within the recorded qualitative traits, only a few (presence of hump, shape and orientation of horns, size of dewlap) were useful in discriminating the zebus from the taurine breeds (S2_Table and S3_Table). The white color was dominant in the zebus except for the zebu *Bodeeji* which generally presented a single reddish brown coat color. Taurine and crossbreed presented variable coat colors. In addition, about half of the animals sampled as of the *Bargouji* breed had no hump, and the same observation was made for the *Keteeji* breed.

Typology of cattle breeds

The canonical coefficients showing the contribution of each measured morphometric trait to the total variation are presented in S4_Table. The first two canonical variates together accounted for 86.26% of the total variation among breeds. The correlation between the cattle breeds and CAN1 was 0.896 and the one between the breeds and CAN2 was 0.771 and the two axes were significant ($P<0.001$) and sufficient to classify all individual cattle studied. The variables height at withers (WH), rump height (RH), scapulo-ischial length (SIL, horn length (HL), body length (BL), hip width (HW), heart girth (HG), chest depth (CD), ear length (EL), and hock circumference (HC) proved to be the most useful in discriminating among the nine cattle breeds investigated.

The plot of the centroid values of these first two canonical discriminant functions (Fig. 2) shows a clear cut separation between zebu and taurine breeds. The taurine breeds of *Boboji* and Somba appeared to be the two most homogeneous groups whereas *Bargouji* animals partially overlap with the *Keteeji*. Furthermore, there was no clear cut separation among zebu breeds and also between zebus and unnamed crossbreeds.

S5_Table shows the pairwise Mahalanobis distances among the nine cattle breeds investigated. All pairwise distances between the breeds were significant ($P<0.001$). The greatest distance was observed between Somba and *Bodeeji* (46.87), followed by Somba and

Fig. 2 ***Scatterplot of 1401 individual animals on the first two canonical discriminant functions*** [*Nearly here*]

Dageeji (33.89) whereas the smallest was observed between *Yakanaji* and *Bodeeji* (2.54) followed by *Yakanaji* and crossbreeds (2.59).

The dendrogram based on the distance matrix (Fig. 3) shows three main clusters: Group 1 included the *Bargouji* breed which was clearly separated from zebu breeds but close to the two taurine breeds of *Somba* and *Boboji* (Cluster 2). The third cluster included all zebu breeds, the unnamed crossbreeds and the *Keteeji*.

Fig. 3 ***Cluster analysis (UPGMA) of cattle breeds based on Mahalanobis distances*** [*Nearly here*]

The two discriminating functions correctly classified about 75 % of the individuals into their a-priori groups (Table 6). However, it is worth noting that about half of the *Yakanaji* and crossbreed individuals were incorrectly classified whereas almost all Somba animals were successfully assigned to their original group.

# Correspondence analysis of breeds' qualitative traits

The Multiple Correspondence Analysis (MCA) of the qualitative characters produced slightly different results from those obtained with the Canonical Discriminant Analysis of the quantitative traits (Fig. 4). The first dimension separated the taurine breeds (Group I) and the group of *Bodeeji* breed (Group II), from a very heterogeneous group (Group III) which included the other zebu breeds, the unnamed crossbreeds, the *Keteeji* and the *Bargouji* breeds. Group I was characterized by spotted and composed aspects of their coat with dominant black, white or

brown colors, cup and numeral three forms of horns and absence of hump whereas Group II was associated with a reddish or red spotted white coat colour. The animals of the third group, which is the most heterogeneous, had a dominant white coat colour.

Fig. 4 ***Multiple correspondence analysis of the morphological traits of cattle breeds*** [*Nearly here*]

Table 6. Percent (%) of individual cattle classified into their a-priori breeds

Breed	Posterior probability (%)									Total
	Bargouji	*Boboji*	*Bodeeji*	*Dageeji*	*Goudali*	*Keteeji*	Crossbreed	Somba	*Yakanaji*	
Bargouji	**80.71**	1.78	0.00	1.19	0.89	11.57	1.48	0.59	1.78	337
Boboji	0.00	**84.13**	0.00	0.00	0.00	0.00	4.76	11.11	0.00	63
Bodeeji	0.00	0.00	**83.33**	2.78	0.00	0.00	2.78	0.00	11.11	36
Dageeji	0.00	0.00	8.33	**75.00**	0.00	0.00	8.33	0.00	8.33	24
Goudali	0.00	0.00	0.00	0.00	**84.38**	0.00	6.25	0.00	9.38	32
Keteeji	9.96	0.43	1.30	0.00	0.00	**83.55**	2.60	0.00	2.16	231
Crossbreed	5.45	11.82	7.27	10.00	5.45	0.00	**49.09**	0.91	10.00	110
Somba	0.52	3.11	0.00	0.00	0.00	0.00	0.52	**95.85**	0.00	193
Yakanaji	4.27	1.33	14.13	15.20	2.67	4.80	20.00	0.00	**37.60**	375
Rate	0.192	0.158	0.166	0.250	0.156	0.164	0.509	0.041	0.624	0.251
Priors	0.111	0.111	0.111	0.111	0.111	0.111	0.111	0.111	0.111	0.111

NB: The percentage of well classified cow is read on the first diagonal (in bold)

Discussion

The originality and the innovative character of our approach in the West African extensive livestock production systems lie in linking herders' description of cattle breeds with metric measures of phenotypes. Herders' accuracy in describing and distinguishing different cattle breeds underlines the relevance of this approach for investigating the phenotypic and genetic variability in farm animal genetic resources. As observed in this study, herders' classification offers the advantage to be easily comparable to the quantitative classification obtained from the metric measures of phenotypes, as the discriminating criteria used by herders are in agreement with those most commonly used in morphological characterization studies in livestock [24].

There was also a considerable consistency in the local names attributed to each of the identified cattle breeds regardless of the geographic location of the herders. Cattle breeds were mainly named in *Fulfulde*, the language of *Fulani* or *Fulbe* people, as the majority of pastoralists in West Africa belong to this ethnic group [50]. The breeds names given by cattle herders have previously been reported by many authors and match with the majority of cattle breeds commonly found in West Africa: *Yakanaji* known as *Daneeji/Akuji/Bunaji* or White Fulani [15,50], *Bokoloji/Goudali* or *Zomanta* in Fongbe (the most common native language spoken in Benin) and known as Sokoto Gudali [15,50], *Bodeeji/Bororo* also called *WoDaaBe*, Red Fulani or Red Bororo [50,51], *Djelliji/Djelli* or *Djalli* known as Peulh Nigérien [52], *Dageeji* also called *Dage* [53], and *Bargouji*/*Bargou* commonly designated as Borgou [54]. Somba and *Keteeji* are also reported in the literature under these same names [46,54,55,56]. *Boboji* was the only cattle name that does not appear in the existing literature. According to its traits, as described by the herders, it is a cattle of small size, humpless with shorthorns, and reported to be mainly found in the southern regions of Benin. The given characteristics are most similar to those of the shorthorn lagoon taurine cattle, commonly known as Lagunaire or Lagune [57].

The findings of this study reveal the diversity of cattle breeds traditionally raised in Benin and confirm that herders have a good knowledge of their animal genetic resources [48]. The number of breeds reported by the surveyed herders in this study is however greater than those reported in previous studies. For instance, a recent study conducted by [48] reported only five cattle breeds (*Keteeji*, *Jaliji/Djelliji*, *Bodeeji*, *Tchiwali/Yakanaji* and Gudali) out of the total of ten recorded in the current study. However, their study differs with respect to geographical focus. Indeed, in contrast to our study which considered seven localities along the geographical north-south gradient in Benin, the investigation by [48] was limited to the Biosphere Reserve of W National Park, in the extreme north of the country. Likewise, the national report to FAO in 2004 [58] also mentioned the presence of eight distinct cattle breeds, omitting the *Dageeji* cattle, which has never been mentioned in previous studies. Main reasons for its neglect may include its small population size and limitation to a few herds in regions not considered in these studies as pastoral areas. Similarly, the Domestic Animal Genetic Resources Information System (DAGRIS), the web-based electronic source of information on selected indigenous farm animal genetic resource [59] reports fewer cattle breeds for Benin than the current study.

Herders' description of cattle breeds clearly differentiated the humpless small-framed *Boboji* and Somba cattle from the humped and larger-framed zebus. This difference between zebu and taurine cattle subspecies was confirmed by the multivariate analysis performed on the recorded metric traits. Similar results have been obtained by [16,60]. In the same way, the closeness of the *Boboji* and Somba breeds to each other in their physical appearance as mentioned by our respondents have been confirmed by the classical methodologies (AHC and MCA) of classifying farm animal genetic resources based on measured morphometric traits. However, the scatterplot displaying all individual animals on the canonical discriminant functions successfully separated these two breeds, with almost no overlap. This finding is consistent with the results from a previous molecular comparison of the two breeds by [60] and suggests that

the morphological characterization may be appropriate and sufficient to study and compare the genetic structure in these two morphologically close breeds.

In contrast, with the exception of the *Bodeeji*, which was separated from others zebu in MCA performed with qualitative traits, the zebu breeds distinguished by herders could not be accurately separated in the multivariate analyses. This underlines the relevance of qualitative information in the morphological description of livestock. Although often considered subjective [16, 61], well-provided information on the qualitative traits of animals could be useful for supplementing quantitative data and may also help in rapidly sampling an animal breed for in-depth studies such as molecular analyses.

The difficulty for a perfect separation of zebu cattle types corroborates the challenges in identifying genetic variation patterns among West African livestock breeds from their morphology as previously reported by [17,62]. These authors explain this situation by the lack of selection and high levels of gene flow due to cyclical cross-border cattle herd movements known as "transhumance" and to extensive commercial transactions of cattle on the hoof in the West African region. For these zebu breeds, further discrimination at the molecular level may be necessary.

The Mahalanobis distance obtained between breeds showed, however, a significant variation in the morphological closeness among breeds. The low values of Mahalanobis distances between *Yakanaji* and several other zebu breeds, for instance *Bodeeji* and *Dageeji*, as well as between *Yakanaji* and unnamed crossbreeds reveal a high degree of overlap in morphological characteristics among these breeds. *Yakanaji*, in fact, appeared to be the most heterogeneous zebu breed in the study area as revealed by the low percentage of individuals from this breed correctly classified in their a-priori group. The aforementioned overlap hampers the

differentiation of these breeds on the exclusive basis of multivariate analyses of their morphometric traits.

The great heterogeneity of the *Yakanaji* cattle might result from their large use in crossbreeding by livestock keepers [57] due to their good production performance and adaptive traits [50,63,64]. Unsupervised and indiscriminate crossbreeding with local cattle breeds, as often encountered, represents an important threat to the conservation and sustainable use of the latter.

In contrast to *Yakanaji*, the highest values of Mahalanobis distances between Gudali and the other zebu cattle breeds are consistent with the specific characteristics of this cattle breed especially its conformation and the absence of horn. Further, this breed is less used in crossbreeding in the surveyed areas. Its sensibility to trypanosomiasis [65,66], an infectious disease caused by a protozoan parasite, could explain its geographical restriction in Benin to the virtually tsetse free Northern Sudanian zone.

Interestingly, there has been a considerable inconsistency in the classification of *Bargouji* and *Keteeji* cattle breeds using multivariate techniques. While the AHC performed on the quantitative traits clearly separated the two cattle types, they were grouped together in the MCA approach using their qualitative traits. In the scatterplot (Fig. 2) as well as in the dendogram (Fig. 3), individuals from the *Bargouji* breed were very close to the taurine breeds (*Somba and Boboji*) in their morphological characteristics whereas *Keteeji* were grouped with zebus and unnamed crossbred. This important overlap between zebus and *Keteeji* is certainly due to the presence of hump in more than half of the individuals recorded as *Keteeji*. But herders considered the “true” *Bargouji* as a humpless animal; they also firmly identified some humped individuals as *Bargouji* and the “true” *Keteeji* as a humped animal. We can therefore argue that there exist two sub-types (one humped and one humpless) in each of the two cattle breeds. These results, in contrast to those obtained with the analysis of the qualitative traits, are in

congruence with herders' classification who exhibited a very good knowledge of the two cattle breeds. It highlights that neither qualitative nor quantitative traits alone are sufficient in breed characterization, but a good combination of both.

But, both cattle breeds of *Bargouji* and *Keteeji* show body size values intermediate between those of taurine and zebu subspecies which certainly explains the difficulty to distinguish them in previous scientific reports. [54] recognized the existence of both the *Keteeji* and the *Bargouji* (*Barguuji or* Borgou according to the author) in Benin but considered the latter as a taurine and the *Keteeji* as a crossbreed between zebu and taurine. According to [56], who reported its presence since 1918 in the Niger Valley in the Northern Sudanian zone of the country, the *Keteeji* is a "crossbreed between the zebu and the small *N'dama* cattle from the more humid south Borgou". Yet, the *N'dama* breed, a longhorn taurine cattle native of Guinea, was introduced to Benin only in 1952 [67]. The absence of *N'dama* cattle among the cattle breeds elicited by the herders participating in the present study is consistent with the progressive disappearance of this breed in Benin, previously highlighted by [58].

Also, the recent study by [48] confounded *Keteeji* to *Borgu* (Borgou). In the official reports on cattle breeds from Benin and West Africa, these two cattle breeds are indiscriminately referred to as "Borgou" cattle, a "stabilized" crossbreed between *Yakanaji* and Somba [68], even though more than half of the Borgou herds were further mated with zebus [69]. One of the issues that emerge from these findings and that has already been pointed out by [7,57] is the heterogeneity in many "stabilized" crossbreeds in West Africa. Hence, our results provide support for the hypothesis that *Keteeji* and *Bargouji* are two varieties of the Borgou cattle. It also confirm the necessity of combining molecular analyses, phenotypic characterization and herders' knowledge for a more accurate differentiation of the breeds and subtypes of cattle raised in extensive African livestock production systems for their effective management and preservation. Several of them have already disappeared before being formally identified [12].

Conclusions

The aim of this study was to validate pastoralists' classification system and knowledge of cattle breeds with quantitative morphometric analyses. The results showed the need to associate qualitative traits and quantitative traits measurement in morphological discrimination of cattle breeds. The findings reveal that livestock keepers have a good knowledge of the morphological trait characteristics of the cattle breeds raised in their herds. The multivariate analyses of the morphometric traits showed less accuracy than herders' classification approach in discriminating most of the zebu breeds because of high variability within and among breeds. The difficulty of perfect separation of these cattle breeds, whatever the single approach of classification, suggests combining livestock keepers' traditional knowledge with phenotypic and molecular genetic approaches as an integrated tool for the appropriate characterization of farm animal genetic resources.

Ethics approval and consent to participate

The study involved taking body measurements from cattle with the consent and in the presence of the cattle farmer. There is no specific legislation for body measurements and hence no approval was necessary. All the data was collected in traditional farms and the animal owners agreed to be involved in the project through the Regional Action Centre for Rural Development (CARDER) which is the decentralized institution for the management of the agricultural sector in the surveyed municipalities. Furthermore, the study did not involve any aversive animal handling, any anesthesia, euthanasia, or kind of animal sacrifice. All the animals included in this study were managed in accordance with the criteria for the assessment of animal welfare identified by the Welfare Quality Project (WQP) reported by [73].

Data Availability

All relevant data are within the paper and its Supporting Information files.

Competing interests

The authors have no competing interests to declare

Funding

This study was financially supported by the VolkswagenStiftung, Hannover, Germany, through the grant Az 89 367.

Author Contributions

Conceptualization: Sandrine O. Houessou, Luc Hippolyte Dossa, Rodrigue Vivien Cao Diogo, Eva Schlecht

Data curation &Formal analysis: Sandrine O. Houessou, Maurice Cossi Ahozonlin, Luc Hippolyte Dossa

Funding acquisition: Luc Hippolyte Dossa

Investigation: Sandrine O. Houessou.

Methodology: Sandrine O. Houessou, Luc Hippolyte Dossa, Mahamadou Dahouda

Writing ± original draft: Sandrine O. Houessou, Luc Hippolyte Dossa.

Writing ± review & editing: Rodrigue Vivien Cao Diogo, Mahamadou Dahouda, Eva Schlecht.

Acknowledgments

The authors express their sincere gratitude to all participating herders for their valuable help and cooperation during the field work.

References

1. Belemsaga D, Lombo Y, Thevenon S, Sylla S. Inventory Analysis of West African Cattle Breeds. In: Makkar HP, Viljoen GJ. (eds) Applications of Gene-Based Technologies for Improving Animal Production and Health in Developing Countries. Springer, Dordrecht; 2005. https://doi.org/10.1007/1-4020-3312-5_15
2. Mwai O, Hanotte O, Kwon YJ, Cho S. African indigenous cattle: unique genetic resources in a rapidly changing world. Asian Australas J. Anim. Sci. 2015; 28(7), 911-921. https://doi.org/10.5713/ajas.15.0002R
3. Dueppen SA. Cattle in the West African savanna: evidence from 1st millennium CE Kirikongo, Burkina Faso. J. Archaeol. Sci. 2012; 39(1), 92-101. https://doi.org/10.1016/j.jas.2011.09.005
4. Hanotte O, Dessie T, Kempe S. Time to Tap Africa's Livestock Genomes. Science. 2010; 328, 1640. http://dx.doi.org/10.1126/science.1186254
5. Hanotte O, Bradley DG, Ochieng JW, Verjee Y, Hill EW, Rege JEO. African pastoralism: genetic imprints of origins and migrations. Science. 2002; 296(5566), 336-339. http://dx.doi.org/10.1126/science.1069878
6. Freeman AR, Meghen CM, Mac Hugh DE, Loftus RT, Achukwi MD, Bado A, et al. Admixture and diversity in West African cattle populations. Mol. Ecol. 2004; 13(11), 3477-3487. https://doi.org/10.1111/j.1365-294X.2004.02311.x

7. Flori L, Thevenon S, Dayo G K, Senou M, Sylla S, Berthier D, et al. Adaptive admixture in the West African bovine hybrid zone: insight from the Borgou population. Mol. Ecol. 2014; 23, 3241–3257. https://doi.org/10.1111/mec.12816

8. Hoffmann I. Climate change and the characterization, breeding and conservation of animal genetic resources. Anim. Genet. 2010; 41(s1), 32-46. http://dx.doi.org/10.1111/j.1365-2052.2010.02043.x

9. Rege JEO, Marshall K, Notenbaert A, Ojango JMK, Okeyo AM. Pro-poor animal improvement and breeding – what can science do? Lives. Sci. 2011; 136, 15-28. https://doi.org/10.1016/j.livsci.2010.09.003

10. Bruford MW, Ginja C, Hoffmann I, Joost S, Orozco-terWengel P, Alberto FJ, et al. Prospects and challenges for the conservation of farm animal genomic resources, 2015-2025. Front. Genet. 2015; 6, 314. https://doi.org/10.3389/fgene.2015.00314

11. Sunderland TC. Food security: why is biodiversity important?. Int. Forest. Rev. 2011; 13(3), 265-274. https://doi.org/10.1505/146554811798293908

12. FAO. The second report on the state of the world's animal genetic resources for food and agriculture, edited by Scherf BD, Pilling D. FAO Commission on Genetic Resources for Food and Agriculture Assessments. Rome, Italy; 2015. [cited 2018 Nov 12] available from: http://www.fao.org/3/a-i4787e.pdf

13. Sokouri DP, Loukou NE, Yapi-Gnaoré CV, Mondeil F, Gnangbe F. Caractérisation phénotypique des bovins à viande (*Bos taurus* et *Bos indicus*) au centre (Bouaké) et au nord (Korhogo) de la Côte d'Ivoire. AGRI. 2007 ; 40, 43-53. https://doi.org/10.1017/S1014233900002182

14. N'goran KE, Yapi Gnaoré CV, Fantodji TA, N'goran A. Caractérisation phénotypique et performances des Vaches laitières de trois régions de Côte d'Ivoire. Arch. Zootec. 2008 ;

57 (220), 415-426. [cited 2018 Jun 17] available from: http://www.redalyc.org/articulo.oa?id=49515034003

15. Yakubu A, Idahor KO, Haruna H, Wheto M, Amusan S. Multivariate analysis of phenotypic differentiation in bunaji and Sokoto gudali cattle. Acta. Agric. Slov. 2010; 96 (2), 75–80. https://doi.org/10.2478/v10014-010-0018-9

16. Traoré A, Koudandé DO, Fernandez I, Soudré A, Granda V, Alvarez I, et al. Geographical assessment of body measurements and qualitative traits in West African cattle. Trop. Anim. Health Prod. 2015; 47(8), 1505-1513. http://dx.doi.org/10.1007/s11250-015-0891-7

17. Álvarez I, Traoré A, Fernández I, Lecomte T, Soudré A, Kaboré A, et al. Assessing introgression of Sahelian zebu genes into native *Bos taurus* breeds in Burkina Faso. Mol. Biol. Rep. 2014; 41, 3745–3754. https://doi.org/10.1007/s11033-014-3239-x

18. Ndiaye NP, Sow A, Dayo GK, Ndiaye S, Sawadogo GJ, Sembène M. Genetic diversity and phylogenetic relationships in local cattle breeds of Senegal based on autosomal microsatellite markers. Vet world. 2015; 8(8), 994. doi: 10.14202/vetworld.2015.994-1005

19. Álvarez I, Pérez Pardal L, Traoré A, Koudandé DO, Fernández I, Soudré A, et al. Differences in genetic structure assessed using Y-chromosome and mitochondrial DNA markers do not shape the contributions to diversity in African sires. J Anim Breed Genet. 2017; 134(5), 393-404. https://doi.org/10.1111/jbg.12278

20. Grema M, Traoré A, Issa M, Hamani M, Abdou M, Soudré A, et al. Short tandem repeat (STR) based genetic diversity and relationship of indigenous Niger cattle. Arch Tierzucht. 2017a ; 60(4), 399-408.

21. Kim SJ, Ka S, Ha JW, Kim J, Yoo D, Kim K, et al. Cattle genome-wide analysis reveals genetic signatures in trypanotolerant N'Dama. BMC genomics. 2017; 18(1), 371. https://doi.org/10.1186/s12864-017-3742-2

22. Delgado JV, Barba C, Camacho ME, Sereno FTPS, Martinez A, Vega-Pla JL. Livestock characterisation in Spain. AGRI. 2001; 29, 7–18. https://doi.org/10.1017/S1014233900005162

23. Lanari MR, Taddeo H, Domingo E, Centeno MP, Gallo L. Phenotypic differentiation of exterior traits in local Criollo goat population in Patagonia (Argentina). Archiv. Tierzucht. Dummerstorf. 2003; 46, 347–356. https://doi.org/10.5194/aab-46-347-2003

24. FAO. Phenotypic Characterization of Animal Genetic Resources. Animal Production and Health Guidelines 2012a; N° 11, FAO, Rome, Italy. [cited 2018 Jun 12] available from: http://www.fao.org/3/i2686e/i2686e00.pdf

25. Dossa LH, Wollny C, Gauly M. Spatial variation in goat populations from Benin as revealed by multivariate analysis of morphological traits. Small Rumin. Res. 2007; 73, 150–159. https://doi.org/10.1016/j.smallrumres.2007.01.003

26. Yakubu A, Ogah DM, Idahor KO. Principal component analysis of the morphostructural indices of White Fulani cattle. Trakia J. Sci. 2009; 7, 67-73. [cited 2018 Jun 12] available from: http://tru.uni-sz.bg/tsj/vol7no2_2009/new_yakubu.pdf

27. Okpeku M, Yakubu A, Peters S, Ozoje M, Ikeobi C, Adebambo O, et al. Application of multivariate principal component analysis to morphological characterization of indigenous goats in Southern Nigeria. Acta Agric. Slov. 2011; 98(2), 101. https://doi.org/10.2478/v10014-011-0026-4

28. Traoré A, Tamboura HH, Kaboré A, Royo LJ, Fernández I, Álvarez I, et al. Multivariate characterization of morphological traits in Burkina Faso sheep. Small Rumin. Res. 2008; 80, 62–67. https://doi.org/10.1016/j.smallrumres.2008.09.011

29. Yakubu A, Akinyemi MO. An evaluation of sexual size dimorphism in Uda sheep using multifactorial discriminant analysis. Acta. Agr. Scand. A-An. 2010; 60(2), 74-78. https://doi.org/10.1080/09064702.2010.502242

30. Birteeb PT, Peters SO, Yakubu A, Adeleke MA, Ozoje MO. Multivariate characterisation of the phenotypic traits of Djallonke and Sahel sheep in Northern Ghana. Trop. Anim. Health Prod. 2012; 45(1), 267-274. https://doi.org/10.1007/s11250-012-0211-4

31. Kefena E, Dessie T, Han JL, Kurtu MY, Rosenbom S, Beja-Pereira A. Morphological diversities and ecozones of Ethiopian horse populations. Anim. Genet. Resour. 2012; 50, 1-12. https://doi.org/10.1017/S2078633612000021

32. Jabbar MA, Swallow BM, Rege JEO. Incorporation of farmer knowledge and preferences in designing breeding policy and conservation strategy for domestic animals. Outlook Agr. 1999; 28(4), 239-243. http://dx.doi.org/10.1007/s11250-005-4253-8

33. Blench R. 'You can't go home again'. Pastoralism in the new millennium. London. Overseas Development Institute; 2001. [cited 2018 Dec 12] available from: https://www.odi.org/resources/docs/6329

34. Ayantunde A A, Duncan AJ, van Wijk MT, Thorne P. Role of herbivores in sustainable agriculture in Sub-Saharan Africa. Animal, 2018; 12(s2), 199-209. https://doi.org/10.1017/S175173111800174X

35. Boutrais J. The Fulani and cattle breeds: crossbreeding and heritage strategies. Africa 2007; 77(1), 18-36. https://doi.org/10.3366/afr.2007.77.1.18

36. Krätli S. Cattle breeding, complexity and mobility in a structurally unpredictable environment: the WoDaaBe herders of Niger. Nomadic Peoples. 2008; 12(1), 11-41. https://doi.org/10.3167/np.2008.120102

37. Holloway L, Morris C, Gilna B, Gibbs D. Choosing and rejecting cattle and sheep: changing discourses and practices of (de) selection in pedigree livestock breeding. Agr. Hum. Values. 2011; 28(4), 533-547. https://doi.org/10.1007/s10460-010-9298-2

38. Kugonza DR, Nabasirye M, Hanotte O, Mpairwe D, Okeyo AM. Pastoralists' indigenous selection criteria and other breeding practices of the long-horned Ankole cattle in Uganda.

Trop. Anim. Health Prod. 2012; 44(3), 557-565. https://doi.org/10.1007/s11250-011-9935-9

39. Brown T. The N'Dama Dilemma: Ethnogenetics and Small Ruminant Breed Dynamics in the Tsetse Zone, the Gambia. Outlook Agri. 2015; 44(3), 173-178. https://doi.org/10.5367/oa.2015.0210

40. Mendelsohn R. The challenge of conserving indigenous domesticated animals. Ecol. Econ. 2003; 45, 501–510. https://doi.org/10.1016/S0921-8009(03)00100-9

41. Wollny CBA. The need to conserve farm animal genetic resources in Africa: should policy makers be concerned?. Ecol. Econ. 2003; 45(3), 341-351. https://doi.org/10.1016/S0921-8009(03)00089-2

42. FAO. Réalisation d'enquêtes et de suivi pour les ressources zoogénétiques. Directives FAO: Production et santé animales. 2012b; Numéro 7, FAO, Rome, Italy. [cited 2017 Feb 15] available from: http://www.fao.org/3/ba0055f/ba0055f00.pdf

43. Traoré A, Koudandé DO, Fernández I, Soudré A, Álvarez I, Diarra S, et al. Multivariate characterization of morphological traits in West African cattle sires. Arch. Anim. Breed. 2016; 59(3), 337-344. http://dx.doi.org/10.5194/aab-59-337-2016

44. Grema M, Traoré A, Issa M, Hamani M, Abdou M, Fernández I, et al. Morphological assessment of Niger Kuri cattle using multivariate methods. S. Afr. J. Anim. Sci. 2017b; 47(4), 505-515. http://dx.doi.org/10.4314/sajas.v47i4.9

45. Moussa M, Issa M, Traoré A, Grema M, Hamani M, Fernández I, et al. Morphological assessment of the Zebu Bororo (Wodaabé) cattle of Niger in the West African zebu framework. Arch. Anim. Breed. 2017; 60(4), 363-371. https://doi.org/10.5194/aab-60-363-2017

46. Dossa LH, Vanvanhossou FUS. The indigenous Somba cattle of the hilly Atacora region in North-West Benin: threats and opportunities for its sustainable use. Trop. Anim. Health Prod. 2016; 48(2), 349-359. https://doi.org/10.1007/s11250-015-0958-5
47. Ayantunde AA, Kango M, Hiernaux P, Udo HMJ, Tabo R. Herders' Perceptions on Ruminant Livestock Breeds and Breeding Management in Southwestern Niger. Hum. Ecol. 2007; 35, 139-149. https://doi.org/10.1007/s10745-006-9049-6
48. Tamou C, de Boer IJ, Ripoll-Bosch R, Oosting SJ. Understanding roles and functions of cattle breeds for pastoralists in Benin. Lives. Sci. 2018; 210, 129-136. https://doi.org/10.1016/j.livsci.2018.02.013
49. SAS (Statistical Analysis System Software), 2008. SAS Version 9.2, SAS Institute Inc, Cary, NC, USA.
50. Blench R. Traditional livestock breeds: Geographical distribution and dynamics in relation to the ecology of West Africa (Vol. 67). London, UK: Overseas Development Institute. 1999. [cited 2018 Dec 15] available from: https://www.odi.org/resources/docs/2766
51. Boutrais J. Zébus, mouches tsé-tsé et techniciens en Adamaoua camerounais. In Robineau C. (Ed. SC.), Les terrains du développement; approches pluri-disciplinaires des économies du Sud, Paris, Orstom, Didactiques, 1992 ; 235-252. [cited 2018 Dec 04] available from: http://horizon.documentation.ird.fr/exl-doc/pleins_textes/divers07/37475.pdf
52. Belli P, Turini J, Harouna A, Garba IA, Pistocchini E, Zecchini M. Critères de sélection des bovins laitiers par les éleveurs autour de Niamey au Niger. Rev. Élev. méd. vét. pays trop. 2008; 61(1), 51-56.
53. Eruola AO, Sholademi AA, Ufoegbune GC, Makinde AA, Iposu SO. Interactive effects of season, breed and grazing location on lactation yield in a tropical wet-and–dry climate of Nigeria. World J. Agric. Sci. 2013; 1(11), 318-322. http://wsrjournals.org/journal/wjas

54. Djedjebi TS. Pastoralistes et la ville au Bénin: Livelihoods en questionnement. Doctorate thesis. University of Leiden, The Netherlands ; 2009.

55. Hall SJ, Gnaho LK, Meghen C. Une enquête sur la race bovine Somba au Bénin. Rev. élev. méd. vét. pays trop. 1995; 48(1), 77-83.

56. Van Driel A. Sharing a Valley. The changing relations between agriculturalists and pastoralists in the Niger Valley of Benin. Research Report no 64 Leiden: African Studies Centre, Leiden University, Leiden, The Netherlands; 2002.

57. Aboagye GS, Tawah CL, Rege JEO. Shorthorn cattle of West and Central Africa. III. Physical, adaptative and special genetic characteristics. World Anim. Rev. 1994; 78: 22-32. [cited 2018 Nov 14] available from: www.fao.org/docrep/T1300T/t1300t0a.htm

58. DE-MAEP (Direction de l'Elevage). Rapport annuel 2003. MAEP, Cotonou Bénin ; 2004. [cited 2018 Jan 17] available from: http://www.fao.org/AG/againfo/programmes/en/genetics/documents/Interlaken/countryreports/Benin.pdf

59. DAGRIS (Domestic Animal Genetic Resources Information System). Breeds in Benin; 2018. [cited 2018 Jul 18] available from: http://dagris.info/countries/152/breeds

60. Moazami-Goudarzi K, Belemsaga DMA, Ceriotti G, Laloë D, Fagbohoun F, Kouagou N'T, et al. Caractérisation de la race bovine Somba à l'aide de marqueurs moléculaires. Rev. élev. méd. vét. pays trop. 2001; 54 (2), 129-138. https://agritrop.cirad.fr/489759/1/ID489759.pdf

61. Desta TT, Ayalew W, Hedge BP. Breed and trait preferences of Sheko cattle keeper in southern Ethiopia. Trop. Anim. Health Prod. 2011; 43, 851-856. https://doi.org/10.1007/s11250-010-9772-2

62. Traoré A, Álvarez I, Fernández I, Pérez-Pardal L, Kaboré A, Ouédraogo-Sanou GMS, et al. Ascertaining gene flow patterns in livestock populations of developing countries: a case

study in Burkina Faso goat. BMC Genet. 2012; 13(1), 35. https://doi.org/10.1186/1471-2156-13-35

63. Alphonsus C, Akpa GN, Barje PP, Finangwai HI, Adamu BD. Comparative evaluation of linear udder and body conformation traits of bunaji and Friesian x bunaji cows. World J. Life Sci. Med. 2012; 2 (4), 134 - 140. http://rrpjournals.org/wjlsmr/en_wjlsmr_vol_2_iss_4_pg_134_140.pdf
64. Pelican M. Friendship among pastoral fulbe in northwest Cameroon. Afr. Study Monogr. 2012; 33(3). 165-188. https://doi.org/10.14989/159427
65. Ogunsanmi AO, Ikede BO, Akpavie SO. Effects of management, season, vegetation zone and breed on the prevalence of bovine trypanosomiasis in South Western Nigeria. Isr. J. Vet. Med. 2000; 55(2), 1−7. https://doi.org/10.4236/oalib.1101521
66. Talabi AO, Otesile EB, Joshua RA, Oladosu LA. Clinical observations on three Nigerian Zebu cattle breeds following experimental *Trypanosoma congolense* infection. Bull. Anim. Hlth. Prod. Afr. 2012; 60 (2), 187−192.
67. Youssao AKI, Ahissou A, Toure Z. Introduction de la race bovine N'Dama à la Ferme Elevage de l'Okpara au Bénin. Quelques performances zootechniques. Anim. Genet. Resour. 2000 ; 27, 17-25. https://doi.org/10.1017/S1014233900001255
68. Porter V. A world dictionary of livestock breeds, types and varieties. 5th ed. CABI Publishing. Wallingford. 2002.
69. Porter V, Alderson L, Hall S, Sponenberg DP. Mason's World Encyclopedia of Livestock Breeds and Breeding. 1st ed. CABI Publishing. Wallingford; 2016.
70. Akoègninou A, Van der Burg WJ, Van Der Masen LJG. Flore analytique du Bénin. Wageningen: éditions Backhuys Publishers; 2006.

71. FAOSTAT, 2017. Country STAT-Benin/Production/ Répartition des effectifs d'animaux vivants par année, Niveau administratif 2. [cited 2018 Nov 30] available from: http://193.43.36.162/home.aspx?c=BEN&ta=053SPD135&tr=21

72. INSAE (Institut National de la Statistique et de l'Analyse Economique). RGPH4 : Que retenir des effectifs de population en 2013 ; 2015 [cited 2018 Nov 17] available from: https://www.insae-bj.org/images/docs/insae-statistiques/demographiques/population/Resultats%20definitifs%20RGPH4.pdf

73. Vapnek J, Chapman M S. Legislative and regulatory options for animal welfare. FAO Legislative Studies No. 104. 2010. [cited 2019 Apr 19] available from: http://www.fao.org/3/i1907e/i1907e01.pdf

Supporting information Captions

S1_Table. Least square means in cm (± standard error) of morphological variables for nine cattle breeds raised in Benin (pooled data for both cows and bulls).

S2_Table. Distribution (in %) of measured qualitative traits among nine cattle breeds raised in Benin.

S3_Table. Distribution (in %) of measured qualitative traits among nine cattle breeds raised in Benin (continued).

S4_Table. Canonical loadings of fifteen measured morphological traits from nine cattle breeds raised in Benin on the first two canonical variables.

S5_Table. Pairwise Squared Mahalanobis distances among nine cattle breeds raised in Benin (based on data measured from both cows and bulls).

Figure 1

Click here to access/download;Figure;Fig1.tif.tif

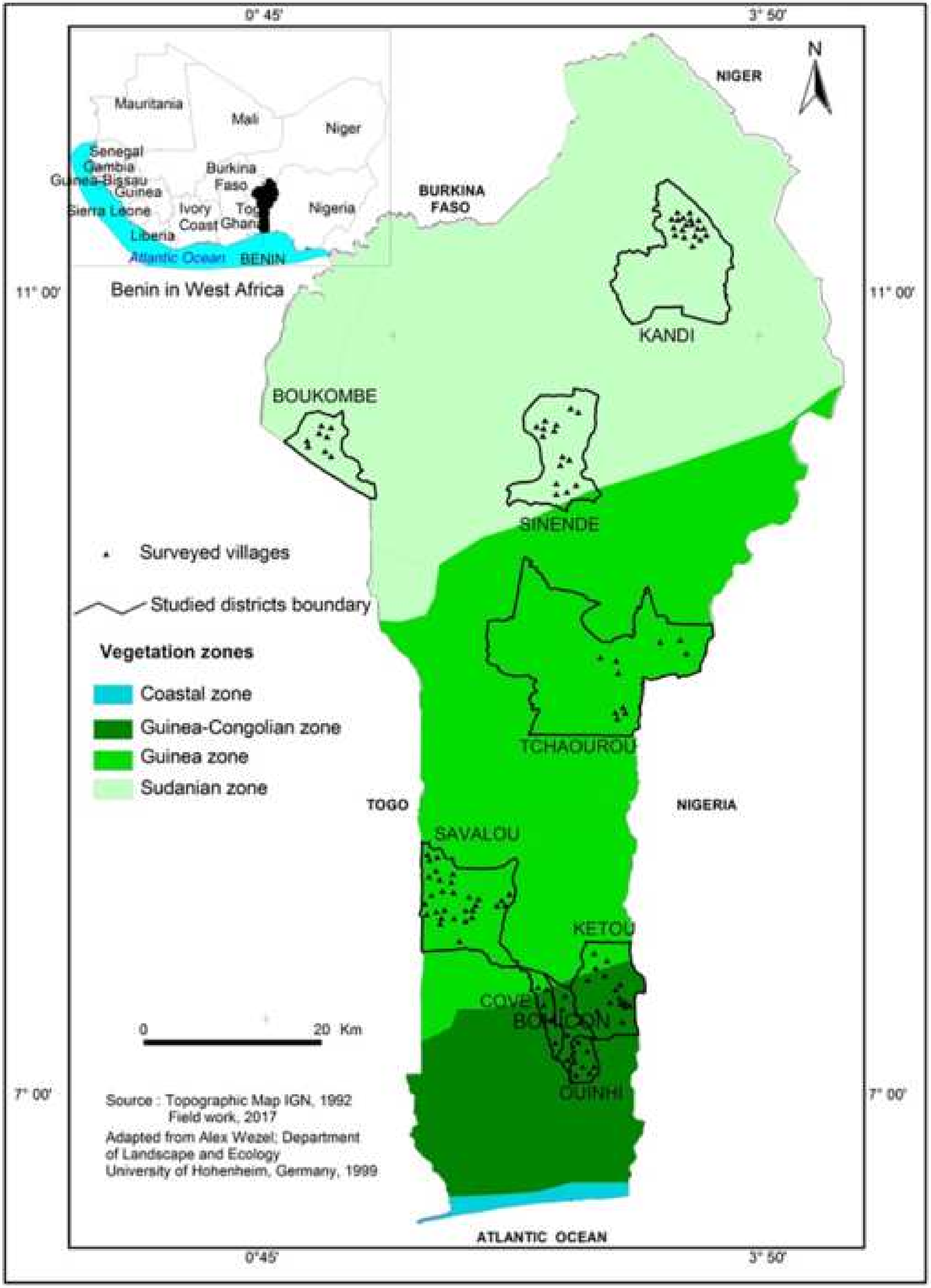

Figure 2

Click here to access/download;Figure;Fig2.tif.tif

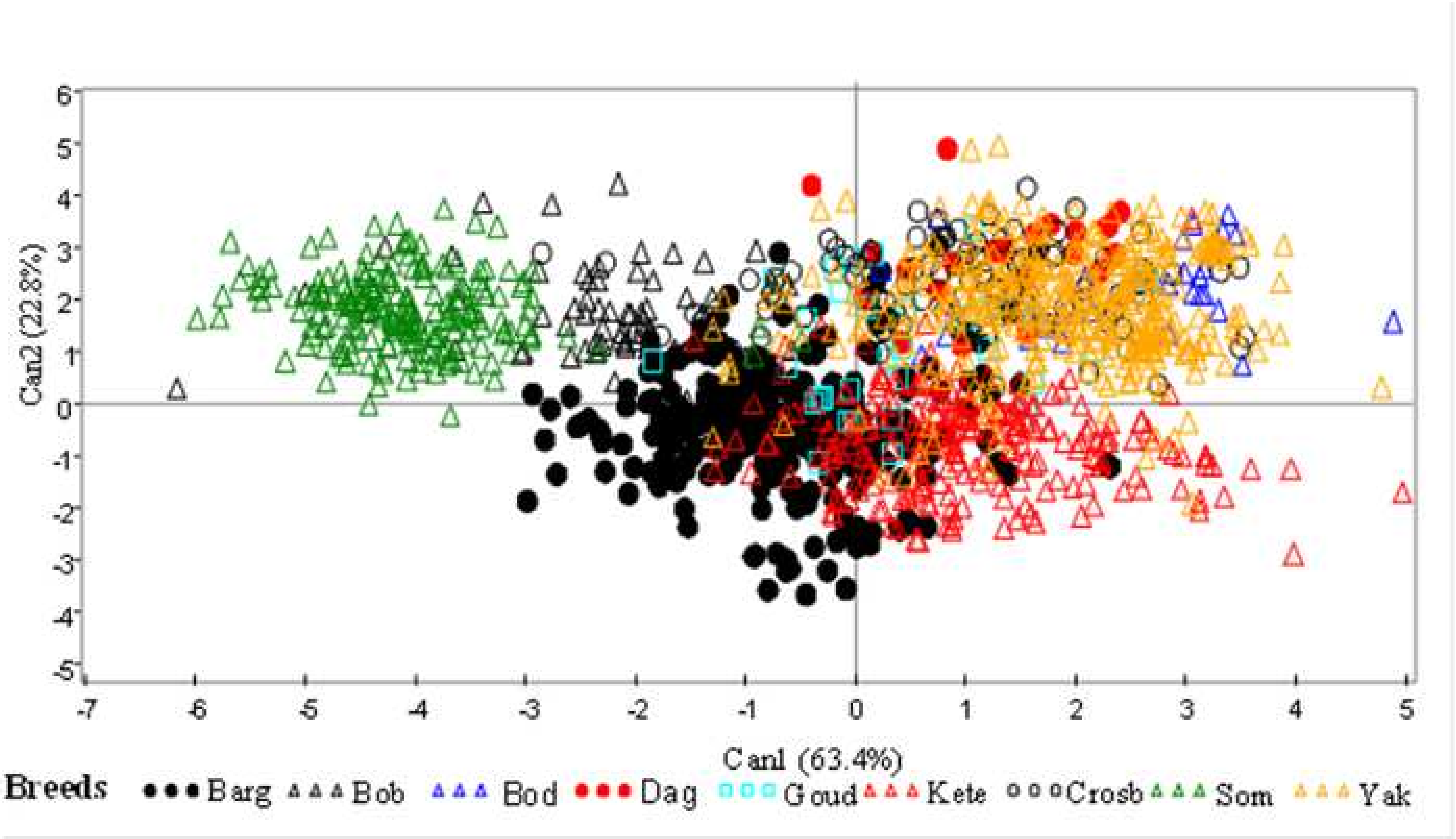

Figure 3

Click here to access/download;Figure;Fig3.tif.tif

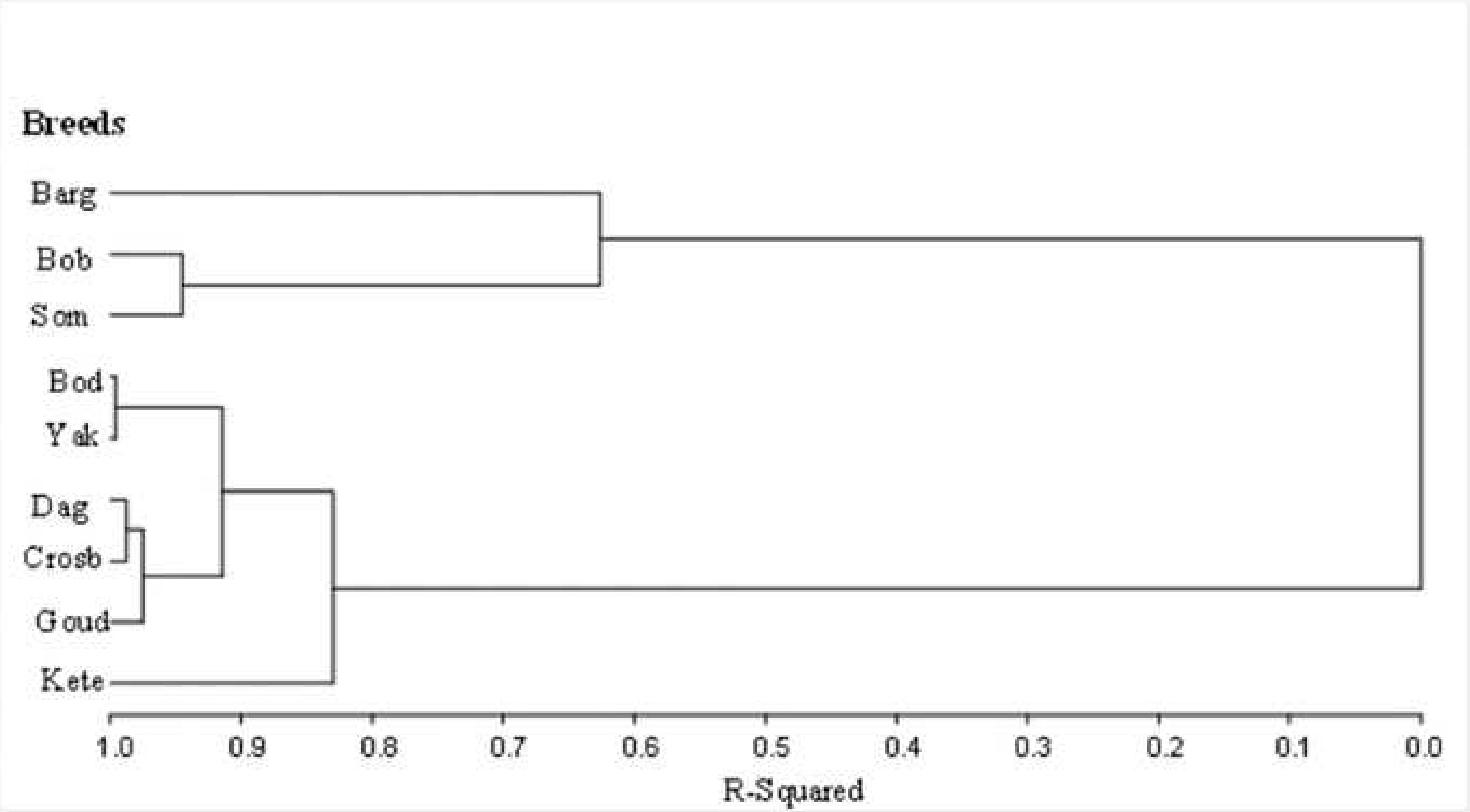

Figure 4

Click here to access/download;Figure;Fig4.tif.tif

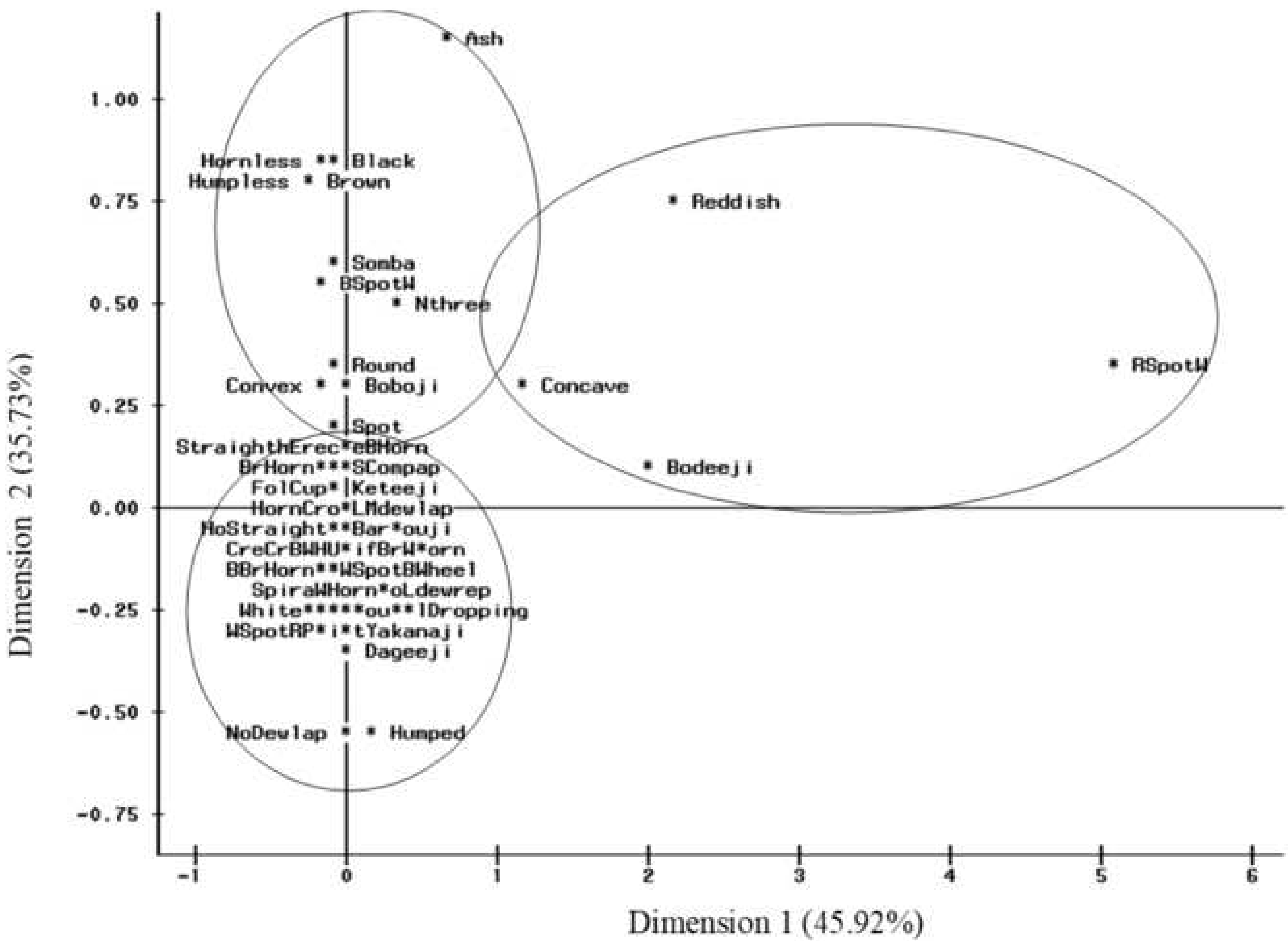

Supplemental table 1

Click here to access/download
Supporting Information
S1_table.pdf.pdf

Supplemental table 2

Click here to access/download
Supporting Information
S2_table.pdf.pdf

Supplemental table 3

Click here to access/download
Supporting Information
S3_table.pdf.pdf

Supplemental table 4

Click here to access/download
Supporting Information
S4_table.pdf.pdf

Supplemental table 5

Click here to access/download
Supporting Information
S5_table.pdf.pdf

Database

Click here to access/download
Supporting Information
Copie de Overall_data.xlsx
