# Supplementary material for: Confronting pastoralists’ knowledge of cattle breeds raised in the extensive production systems of Benin with multivariate analyses of morphological traits
Source: PLoS One. 2019 Sep 26;14(9):e0222756. doi: 10.1371/journal.pone.0222756 (PMC6762103; doi:10.1371/journal.pone.0222756)
Supplement: Attachment — Submitted filename: Responses to reviewers.docx [file pone.0222756.s016.docx]

**Responses to reviewers’ comments**

| Segment of the manuscript | **Comments** | **Responses** |
| --- | --- | --- |
| **Reviewer/Academic Editor (provided pdf file)** |  |  |
| Introduction | Recent analysis has shown there are no pure Bos Indicus in Africa - delete and just keep the term Zebu | We thank the editor for this comment. We Have delete “*Bos indicus*” as suggested. ***Line 52*** |
|  | Rephrase 'they are mostly characterised by various adaptive and performance traits' as it doesn't add much meaning. E.g. to 'adaptive traits they are characterised by inlcude......' | The sentence has been revised as follows:  *They are appreciated for their adaptive traits which include the resistance to diseases and drought, ability to walk long distances, and capacity to survive on poor pastures [2].* ***Line 54-56*** |
|  | I don't agree with high fertility - reproductive performance of African cattle is typically very low, often because of the poor management conditions / harsh environment. This should be removed. | This was reported in the review paper by Mwai et al 2015. But as suggested by the reviewer, we have removed “high fertility”. ***Line 54-56*** |
|  | Comment L72-This sentence “Indeed, the characterization of livestock breeds can be performed through well-designed phenotypic, genetic and molecular studies that include pertinent and well thought-out analysis and interpretation of quantitative data.” seems to contradict the previous where you suggest that only doing phenotypic, genotypic and molecular characterisation is not sufficient. .....I think it is a grammer problem (more than the point you are trying to make) | We thank the editor for this remark. While shortening the introduction as suggested by most reviewers, this sentence has been removed. |
|  | Comment on L80-Yes and also all the other phenotypic chacterisations that are beyond morphological e.g. calving interal, milk yield, disease resistance and so forth. | We thank the editor for this important detail. |
|  | Comment L98-Here I would suggest changing no studies to limited studies, or 'no studies of which we are aware' | We thank the editor for this comment. Changes have been made as follows:  *So far, however, no studies of which we are aware combine the two approaches in order to test their complementarity and validate pastoralists’ classification of cattle breeds.* ***Line 93-95*** |
|  | Comment L102-The length of the introduction should be reduced by at-least one-third.  Also here, or in the methodology, the concept of breed needs to be explained and the definition of breed you are using in this article given (see e.g. SoW AnGR_ | *We thank the editor for the comments.*  *We have reduced the length of introduction section by about one-third of the initial characters.*  *A discussion has also been provided in Material and methods section on the meaning of “breed” as suggested by the editor as follows:*  *Breed was defined according to [43] as: “either a sub-specific group of domestic livestock with definable and identifiable external characteristics that enable it to be separated by visual appraisal from other similarly defined groups within the same species, or a group for which geographical and/or cultural separation from phenotypically similar groups has led to acceptance of its separate identity”. Thus, cattle breed in this study is referred to cattle of similar physical features perceived by herders as being of the same genetic origin. [Line 137-142]* |
| Material and methods | This paragraph can be consdierably shortened - you don't need to mention the things your study did not do (adverse animal handling etc.) | Following the reviewer’s suggestion the paragraph has been shortened. *[Line 119-126]* |
|  | This paragraph can be said more briefly - all these details are not reqwuired (community sensitisation is standard practice before any research engagement)  Again remove unncecessary details e.g. the statement about arranging a suitable time  Add dates of the study | Following the reviewer’s suggestion some details have been removed. But, other important information as requested by the reviewers (dates of the study, the way of herders’ population sampling selection, the discussion about the meaning of “breed”) have been kept in the paragraph. |
|  | be consistent on use of 'or' vs '/' | We thank the editor for this observation. Changes have been made accordingly. |
|  |  |  |
| **Reviewer1** | **Comments** | **Responses** |
| General comments | The paper Confronting pastoralists' knowledge of cattle breeds raised in the extensive production systems of Benin with multivariate analyses of morphological traits is a good paper. | We thank the reviewer for his encouraging comments on the manuscript. |
| Material and methods | 1. In Table 1:) Suggest drop this information [Region; Agroecological  zone (a); Soil type (a); Rainy season] on the table. Write in Material  and Method (text body) | We have revised the Table1 and added the information in the text body as suggested by reviewer  [Lines 101-108] |
|  | 2. In Line 58: Version of SAS (2008) - is a paid program. Need use a  actual version or use a SAS Student; (49. SAS (Statistical Analysis  System Software), 2008. SAS Version 9.2, SAS Institute Inc, 512 Cary,  NC, USA.) | The citation has been revised accordingly  [Line 167-168] |
|  | 3.The authors can insert an image et one cattle bread (Supporting information); Need insert the general names of each breed if had. | Animal breed images have been added in supporting information as suggested. |
|  | 4.On the table 3: what's means Trait expression ;;) coat color  Variable? | Variable named “Coat color” referred to the “Body hair coat color” as suggested in a scientific reference paper [Food and Agriculture Organization. (2012). Phenotypic characterization of animal genetic resources.] |
|  |  |  |
| **Reviewers2** | **Comments** | **Responses** |
| General comment | This paper is valuable in that it helps to fill the large data-gap on African cattle. The paper is also well written and statistically sounds. | We thank the reviewer for his encouraging comment. |
| Introduction | (a) reduce the length of the introduction | We have reduced the length of introduction section by about one-third. |
| Material and methods | (b) add a disccussion on the meaning of 'breed' which is subjective, and how you define it in this work. | A discussion has been provided on the meaning of “breed” as suggested by reviewer as follow:  Breed was defined according to [43] as: “either a sub-specific group of domestic livestock with definable and identifiable external characteristics that enable it to be separated by visual appraisal from other similarly defined groups within the same species, or a group for which geographical and/or cultural separation from phenotypically similar groups has led to acceptance of its separate identity”. Thus, cattle breed in this study is referred to cattle of similar physical features identify by herders as being of the same genetic origin.  [lines 137-142] |
|  | If you do have tissue / blood / hair samples from the animals you measured, it would be extremely interesting to compare genomic results to those presented here. | We confirm that hair samples have been collected and cattle breeds presented in the manuscript are being compared on the genomic scale in a subsequent study. |
|  |  |  |
| **Reviewers 3** | **Comments** | **Responses** |
| General comment | Comments about the manuscript entitled "Confronting pastoralists'  knowledge of cattle breeds raised in the extensive production systems of  Benin with multivariate analyses of morphological traits".  Authors investigated on the pastoralists' knowledge about of the cattle breeds raised in Benin. Methods used were clearly explained, but some precisions are necessary. Obtained results must be encouraged.  In general, this manuscript can be accepted for publication. Further remarks are given below. | We thank the reviewer for his comments and observations; We have revised the manuscript accordingly. |
| Abstract | L21: change "characteristics-" to "characteristics." | We thank the reviewer for his observation. Changes have been made accordingly. [L33] |
|  | L34: Change ''keepers'' to ''herders'' and harmonize it in all the text | We thank the reviewer for his observation. Changes have been made accordingly throughout the document |
| Introduction | L56-58: Add reference sources | Reference has been added [L68] |
|  | L91-95: Change ''[42] further argues that in traditional livestock production systems, …. phenotypic traits.'' to ''Further, in traditional livestock production systems, …. phenotypic traits [42].'' | This sentence has been removed while reducing the introduction length as suggested by another reviewer. |
|  | L97-98: Move ''[47,48]'' at the end of the sentence | This sentence has also been removed in order to reduce the introduction length as suggested by another reviewer. |
|  | L99: Change ''with the aim'' to ''in order to'' | This sentence has been revised accordingly. [L94] |
|  | L100-102: Rephrase to show clearly the study aim like that is in the  abstract | The study aim has been rephrased as suggested by reviewer as follows:  *This study aimed to document and validate herders' knowledge of differences among cattle breeds raised in Benin with quantitative data. [line 95-96]* |
| Material and methods | How were interviewed herders sample population fixed? The study may  justify that | The sample population was fixed as follows:  *Sampling herds were chosen among those hold by herders who have at least 10 years of experience in cattle herding and supposed as resident herders (herders that reside in the municipality for 05 years at least).*  We have now provided this information in the text by revising the sentence on Lines 132-134 as follows “*Individual interviews were then conducted from June 2015 to June 2016 with 803 resident cattle herders randomly selected from those who had at least 10 years of experience in cattle herding and had spent at least five (05) years in the locality*”. |
|  | Table 1: Change ''(a)'' to [70]; ''(b)'' to [72] and ''(c)'' to [71]  Table 1: Change ''Estimated total cattle population'' to ''Estimating of  the total cattle population'' | Changes have been made in Table 1 as suggested. |
|  | L1: Harmonize expression ''cattle farmer'' or ''cattle herder'' in the  all of the text | We thank the reviewer for this observation. We have made changes throughout the document as suggested. |
|  | L6-7: CARDER was the departmental institution. So Communal Sector of the  Agricultural Development (SCDA) was the local institution in the  municipalities. | Thanks again for the remark. Change has been made as suggested. [L122-124] |
|  | L11: Delete ''reported by'' | Change has been made as suggested. |
|  | L17: Rephrase ''identify the villages'' | The expression has been removed as suggested by another reviewer. |
|  | L59-60: Why this exclusion? Were these 6 animals included in the 1401  individual cattle? | We thank the reviewer for this observation. We have removed the six animals given the too small size of sampled individual measured in each of these breeds (five individual cattle of *Djelliji* breed and only one male of *Dageeji* breed), which could further compromise statistical comparison between breed.  No, the 6 excluded animals were not included in the 1401 individual cattle. |
| Results |  |  |
|  | L88-89: What were the language of these breed names? | The language of the breed name was “*Fulfulde* language”. This has been mentioned in results [***Line 200]*** and in discussion section [***Line 320]*** |
|  | L97-99: What about of Keteeji Bargouji ? are they taurine or zebu  breeds? | Keteeji and Bargouji are crossbreed animals between taurine and zebu breed. They may be referred to Borgou cattle but with a difference in zebu blood level. This difference has been discussed in the discussion section [***Lines 396-418***] |
|  | L105: Table 3: 8 breeds were described. What about of ''Keteeji'‘? | We thank the reviewer for this remark. Using these key morphological traits, herders did not sharply distinguish Bargouji from Keteeji, except for the larger in size than Bargouji. This has been mentioned in result section ***Lines 223-230*** and discussed in discussion section ***Line 396-418*** |
|  | L105: Table 3: Goudali or Gudali? Harmonize it  ok | We have retained “*Goudali*” and harmonize throughout the text. |
| Discussion |  |  |
|  | L207: Write ''Fongbe language'' | Change has been made as suggested. ***Line 324*** |
|  | L219-221: Rephrase: ''For instance, a recent study conducted in ???  reported only five cattle breeds (Keteeji, Jaliji/Djelliji, Bodeeji,  Tchiwali/Yakanaji and Gudali) [48] out of the total of ten recorded in  the current study'' | The sentence has been revised and study area has been added as follow:  For instance, a recent study conducted in northern Benin [53]  reported only five cattle breeds (Keteeji, Jaliji/Djelliji, Bodeeji,  Tchiwali/Yakanaji and Gudali) out of the total of ten recorded in the current study. [***Lines 336-338]*** |
|  | L221: Change ''their study'' to ''the last authors study'' | Change has been made as suggested. ***Line 338*** |
|  | L234: Rephrase ''by [16,60]'' to ''through others studies [16,60]" | Change has been made as suggested. ***Line 352*** |
|  | L252: Rephrase ''as previously reported by [17,62]'' to ''as previously  reported [17,62]''  Ovoid ''by […]'' and harmonize it in the all of the text. | Change has been made as suggested. ***Line 371***  And “by” has also been avoided accordingly in all the text. |
